# Joint Venture of Metal Cluster and Amphiphilic Cationic Minidendron Resulting in Near Infrared Emissive Lamellar Ionic Liquid Crystals

**DOI:** 10.1002/chem.202103446

**Published:** 2021-11-29

**Authors:** Max Ebert, Irene Carrasco, Noée Dumait, Wolfgang Frey, Angelika Baro, Anna Zens, Matthias Lehmann, Gregory Taupier, Stephane Cordier, Emmanuel Jacques, Yann Molard, Sabine Laschat

**Affiliations:** ^1^ Institut für Organische Chemie Universität Stuttgart Pfaffenwaldring 55 70569 Stuttgart Germany; ^2^ Université de Rennes 1 CNRS ISCR–UMR 6226 ScanMAT-UMS 2001 IETR–UMR 6164 35000 Rennes France; ^3^ Institut für Organische Chemie Universität Würzburg Am Hubland 97074 Würzburg Germany; ^4^ Center for Nanosystems Chemistry and Bavarian Polymer Institute Theodor-Boveri-Weg 4 97074 Würzburg Germany

**Keywords:** cluster compounds, ionic liquid crystals, luminescence, organic-inorganic hybrid composites, X-ray diffraction

## Abstract

Inorganic red‐NIR emissive materials are particularly relevant in many fields like optoelectronic, bioimaging or solar cells. Benefiting from their emission in devices implies their integration in easy‐to‐handle materials like liquid crystals, whose long‐range ordering and self‐healing abilities could be exploited and influence emission. Herein, we present red‐NIR emissive hybrid materials obtained with phosphorescent octahedral molybdenum cluster anions electrostatically associated with amphiphilic guanidinium minidendrons. Polarized optical microscopy and X‐ray analysis show that while the minidendron chloride salts self‐organize into columnar phases, their association with the dianionic metal cluster leads to layered phases. Steady‐state and time‐resolved emission investigations demonstrate the influence of the minidendron alkyl chain length on the phosphorescence of the metal cluster core.

## Graphical abstract

**Guanidinium minidendrons bearing terminal aliphatic chains** show a columnar liquid‐crystalline behaviour once associated with chlorine anions, whereas they turned into layered phases when associated with phosphorescent octahedral molybdenum cluster dianions. The emission properties of the inorganic dyes are largely influenced by the minidendron chain length in these first‐of‐their‐kind clustomesogens.

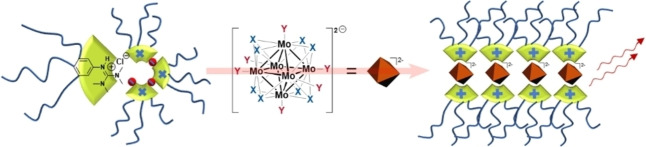

## Introduction

**Scheme 1:**
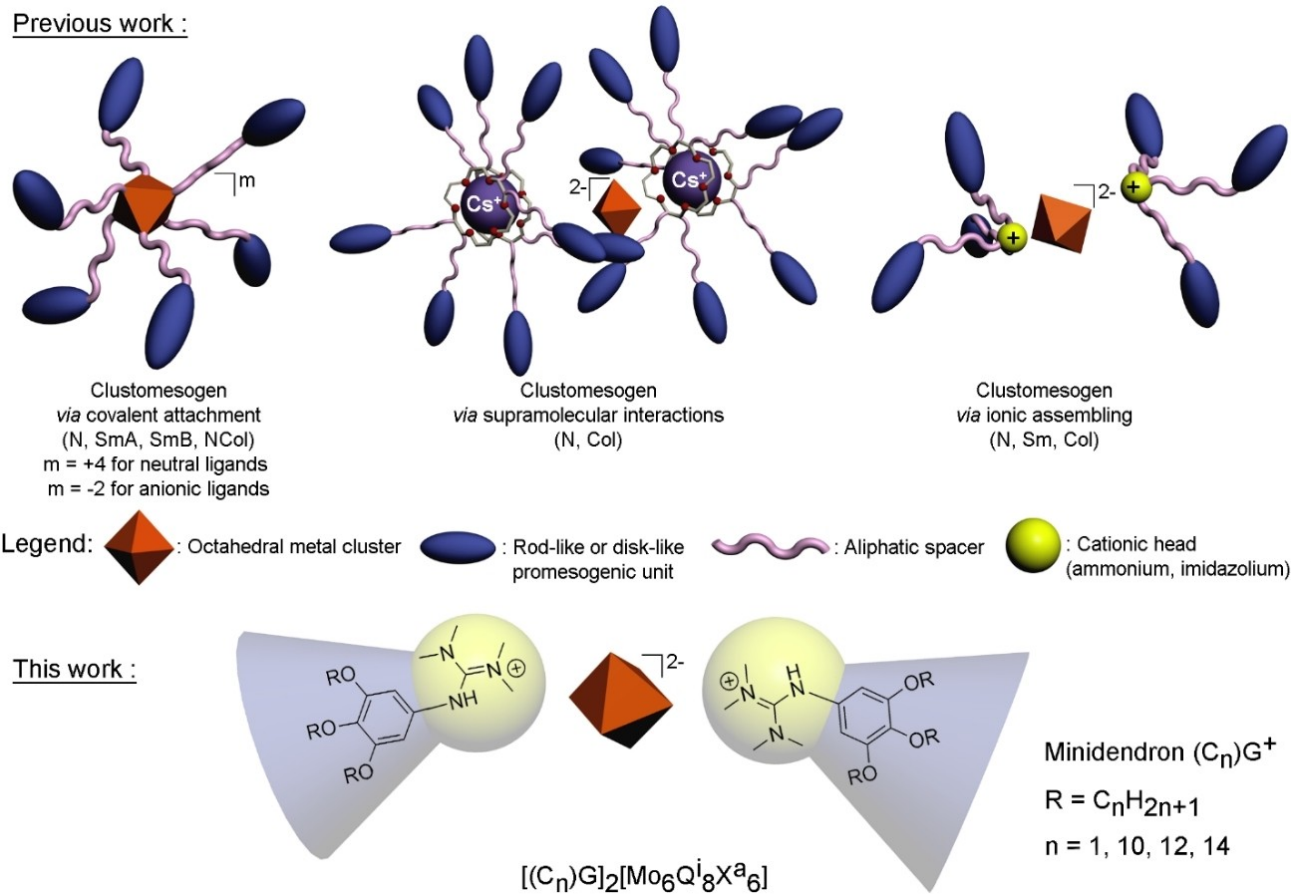
Schematic presentation of clustomesogens obtained previously by associating octahedral clusters with organic ligands terminated by rod‐like or disk‐like promesogenic units and the targeted mesomorphic hybrid compounds.

Materials emitting light in the near infrared (NIR) spectral region possess attractive properties such as low autofluorescence interference, NIR to white light upconversion, deep tissue penetration and minimal photodamage to biological samples. These features make them highly attractive for applications ranging from telecommunication, organic light emitting diodes (OLEDs), solar cells to sensors, bioimaging, medical diagnosis and food testing. Besides organic dyes, metal complexes such as rare earth luminophores, metal clusters, nanoparticles, hybrid perovskite nanocrystal and quantum dots have been used as NIR emitters.^[1]^ Adding liquid crystal properties to emissive materials is particularly relevant as the nanostructuration of molecules in the different mesophases might lead to polarized emission or to a better mobility of charge carriers.^[2]^ Among all available NIR emitters, octahedral metal cluster compounds of the general formula A_x_M_6_Q^i^_8_X^a^_6_ (A=alkali cation, M=Mo, W and x=2 ; M=Re and x=4, Q^i^=face‐capping ligand (i stands for inner and Q=halogen or chalcogen), X^a^=terminal ligand (a stands for apical, X=halogen, hydroxo or cyano) are particularly promising red‐NIR emitters by one‐or two‐photon absorption with high and tuneable quantum yields, large Stokes shift and lifetimes exceeding microseconds.^[3]^ A major drawback of metal cluster solid state compounds is their ceramic‐like behaviour resulting in poor processability. To overcome these difficulties, highly soluble intermediates should be first obtained by cationic metathesis and/or apical ligand exchanges. Afterwards, cluster compounds can be integrated in hybrid nanomaterials via (a) the covalent attachment of organic ligands to the metal cluster core, (b) supramolecular interactions through formation of host‐guest complexes, and (c) ionic self‐assembly of the cluster anion with functional cations (Scheme 1).^[4]^ When the ligands used for these three approaches are either liquid‐crystalline themselves or promote mesophase formation, the resulting hybrid materials self‐assemble into ionic liquid‐crystalline (ILC) phases.^[5]^ This self‐assembly of the hybrid ILCs leads to highly useful properties such as long‐range orientational order and self‐healing of defects together with convenient processing in common organic solvents. This is advantageous for applications such as OLEDs, which require a good alignment and convenient solution processing for good device performance.^[6]^ Up to now clustomesogen were designed by associating the bulky inorganic core with organic synthons containing rod‐like (cyanobiphenyloxy units)[^2c^, ^7^] or disk‐like (triphenylene units)^[8b]^ promesogenic moieties whose mobility was ensured by an aliphatic spacer. Such associations gave rise to columnar, smectic, or nematic phases depending on the geometry of the promesogenic units and/or their density around the bulky inorganic core.[^3^, ^8^] Hence, clustomesogens containing amphiphilic cations have never been described so far, though they could be obtained at lower synthetic cost compared to previously described ones. In fact, examples of polyionic hybrid systems showing mesomorphism and containing a bulky inorganic polyanion and polycatenar cations are scarce. They are mainly based on the association of highly charged polyoxometalate with commercially available organic surfactants bearing one or two long alkyl tails.^[9]^ Therefore, using amphiphilic cations to generate ILC clustomesogen is highly challenging as i) the alkyl chains transverse cross section is low compared to the one of rod‐like cyanobiphenyl units or disk‐like triphenylene units and thus the ratio of organic‐inorganic volume fractions should not favour mesomorphism,^[5]^ ii) the 2‐ anionic cluster core charge does not allow a high ratio of organic‐inorganic volume fractions without a tedious organic molecular engineering work involving highly dendronized organic lipophilic cations, and iii) strong electrostatic interactions between the cationic head and the bulky octahedral metal cluster polyanion should in principle modify strongly the supramolecular association of those cations compared to monoatomic anions. Here we describe the first examples of clustomesogens made of inorganic nanocluster dianions associated with promesogenic wedge‐shaped cationic amphiphilic minidendrons via ionic self‐assembly.

## Results and Discussion

### Synthesis of Clustomesogens

**Scheme 2:**
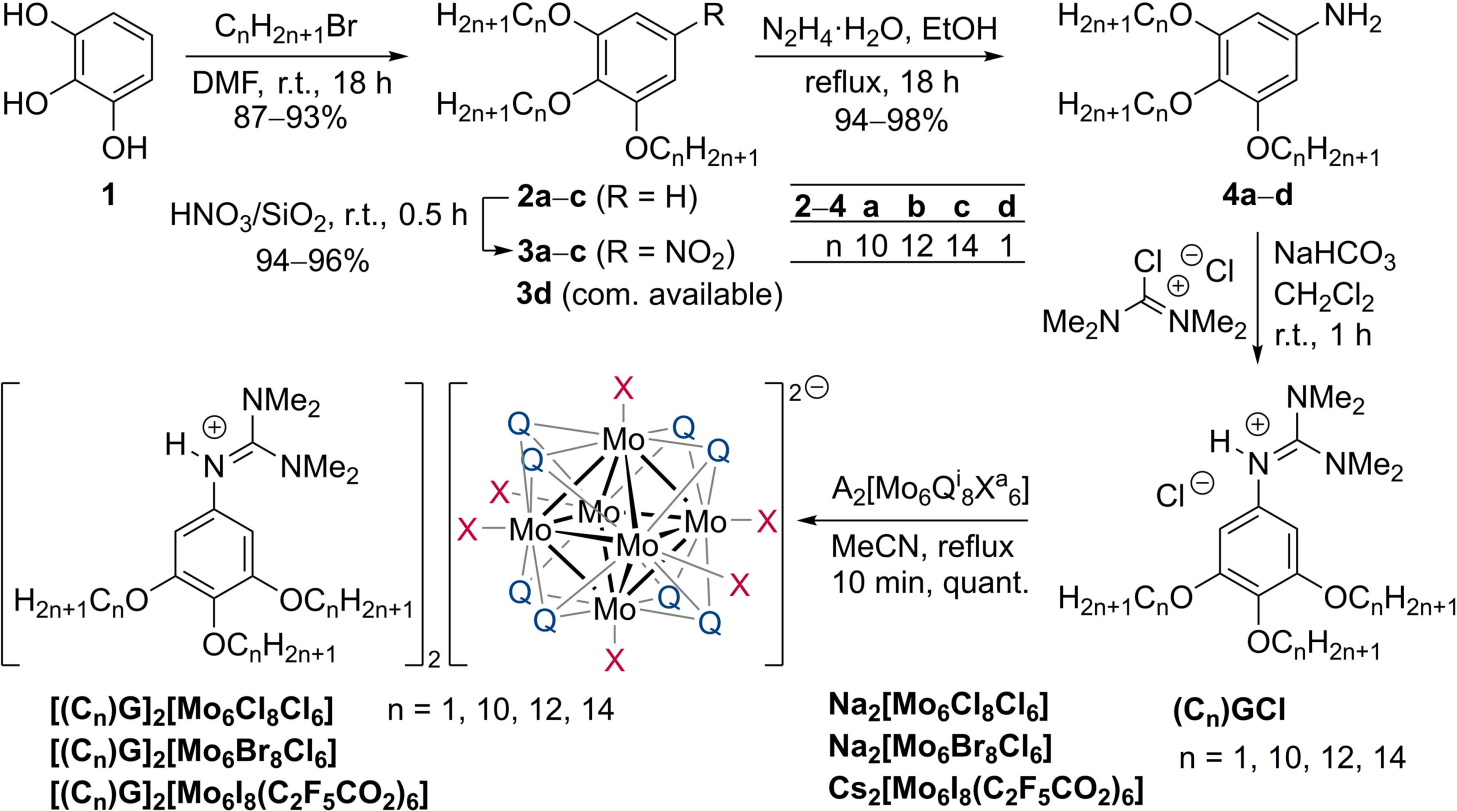
Overview over the synthesis of the studied compounds **[(C_n_)G]_2_[Mo_6_Q^i^**_**8**_ **X^a^**_**6**_ **]**.

The synthesis of clustomesogens commenced with Williamson etherification of pyrogallol, followed by nitration, reduction, and subsequent treatment of anilines with tetramethylformamidinium chloride^[10]^ to give the amidinium chlorides **(C_n_)GCl** (n=1, 10, 12, 14) (see Scheme 2). Three phosphorescent cluster compounds were chosen as precursors for this study, namely Na_2_[Mo_6_Cl^i^_8_Cl^a^_6_], Na_2_[Mo_6_Br^i^_8_Cl^a^_6_] and Cs_2_[Mo_6_I^i^_8_(C_2_F_5_CO_2_)^a^_6_]. While the two first ones could give us some hints about the influence of inner ligands on the hybrid's mesomorphic behaviour, the third one is one of the most sensitive to oxygen quenching. Therefore, as the oxygen permeability of a material is related to its structuration at the nanometre and micrometre scale, [Mo_6_I^i^_8_(C_2_F_5_CO_2_)^a^_6_]^2−^ could act as a probe of the self‐organization process. The desired clustomesogens **[(C_n_)G]_2_[Mo_6_Q^i^**_**8**_ **X^a^**_**6**_ **]** were obtained after a metathesis reaction with the corresponding guanidinium chloride **(C_n_)GCl** as yellow‐orange solids in quantitative yield.

**Figure 1:**
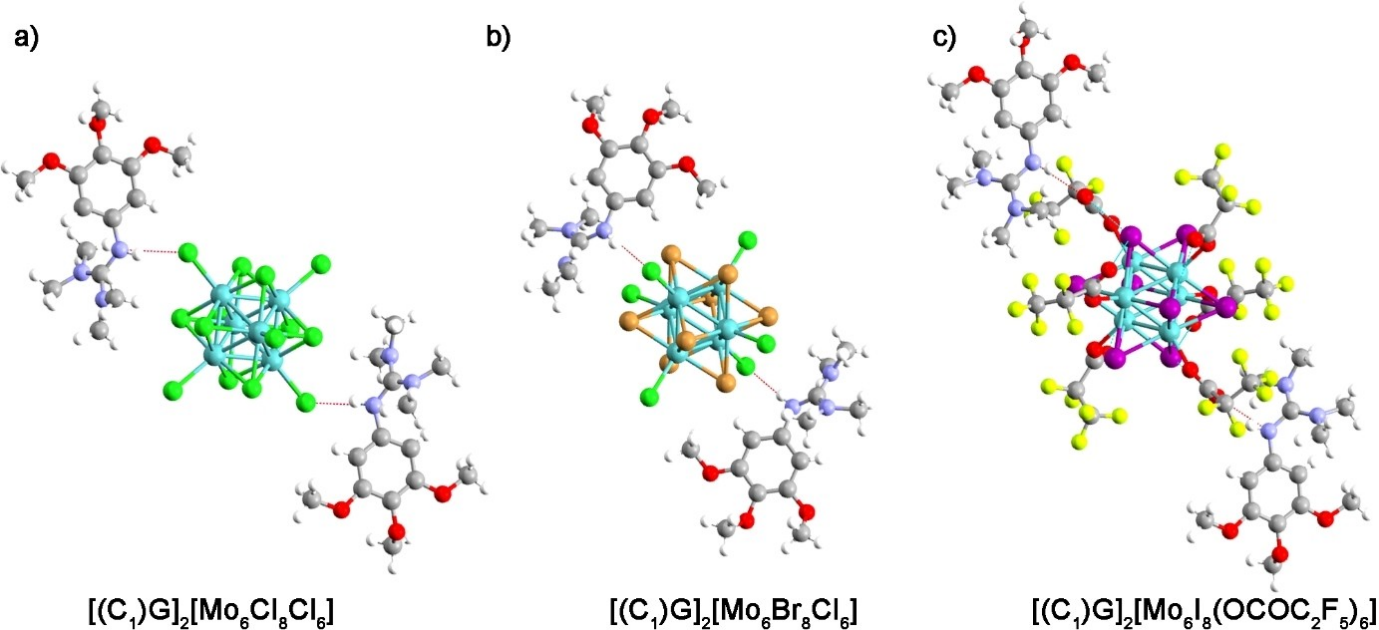
Evidencing from single crystal X‐ray diffraction analysis, H‐Bonds are formed between **(C_1_)G** ^+^ counter cations and the apical ligands of the anionic cluster (a) [Mo_6_Cl_8_Cl_6_]^2−^, b) [Mo_6_Br_8_Cl_6_]^2−^ and c) [Mo_6_I_8_(C_2_F_5_CO_2_)_6_]^2−^.

Fortunately, for all guanidinium salts bearing methoxy groups, i.e. **(C_1_)GCl**, **[(C_1_)G]_2_[Mo_6_Cl_8_Cl_6_], [(C_1_)G]_2_[Mo_6_Br_8_Cl_6_]** and **[(C_1_)G]_2_[Mo_6_I_8_(C_2_F_5_CO_2_)_6_]**, single crystal X‐ray structure analyses could be performed (Figure 1, see Supporting Information for a full description of structures). Intermolecular hydrogen bonds are observed in all cases between the N−H donor of the guanidinium cation and its counter‐anion. Such interaction occurs with an apical chloride ligand as acceptor for [Mo_6_Cl_8_Cl_6_]^2−^ or [Mo_6_Br_8_Cl_6_]^2−^ and with a carbonyl oxygen of a pentafluoro‐propionate moiety for [Mo_6_I_8_(C_2_F_5_CO_2_)_6_]^2−^. In this last case, oxygen atoms from the carbonyl function interact also with iodine inner ligands which restricts the molecular motion of the pentafluoro propionate moieties. Such H‐Bond may play an important role in the structuration of hybrids in the mesophase when methoxy groups are replaced by long alkyl chains. Such derivatives show LC behaviour which will be discussed in next section.

### Mesomorphic Properties of Clustomesogens

Mesomorphic properties were studied by polarizing optical microscopy (POM), differential scanning calorimetry (DSC) and wide and small angle X‐ray scattering (WAXS, SAXS). All guanidinium salts showed enantiotropic liquid‐crystalline phases (Table 1, for details of the DSC curves see Figure S3–S14 in the Supporting Information). For guanidinium chlorides **(C_n_)GCl** both melting and clearing transitions showed no obvious dependence on chain lengths. For example, guanidinium chloride **(C_14_)GCl** with C_14_ side chains showed in the DSC melting and clearing transitions at 35 °C and 86 °C respectively upon heating and only a small hysteresis in the cooling cycle (Figure S5). Upon replacement of chloride counterion by the cluster anions clearing points displayed only small changes for clustomesogens carrying [Mo_6_Cl_8_Cl_6_]^2−^ or [Mo_6_Br_8_Cl_6_]^2−^. In contrast, clustomesogens with [Mo_6_I_8_(C_2_F_5_CO_2_)_6_]^2−^ were already liquid‐crystalline at subambient temperature and we observed a significant decrease of both melting and clearing points as compared to the clustomesogens with the other anions. Clearing temperatures increased slightly with increasing chain length. For example, **[(C_10_)G]_2_[Mo_6_I_8_(OCOC_2_F_5_)_6_]** was liquid‐crystalline at room temperature and cleared into the isotropic phase at 59 °C upon heating under POM and a similar temperature was observed upon cooling. It should be noted that the DSC did not show any peaks for this derivative. In comparison, **[(C_14_)G]_2_[Mo_6_I_8_(C_2_F_5_CO_2_)_6_]** displayed a melting transition at 11 °C in the DSC upon heating, while the clearing transition at 74 °C was only visible under the POM. During cooling the transitions appeared at 74 °C (via POM) and 2 °C (via DSC) respectively.

**Table 1:** Phase transitions, corresponding enthalpies (calculated by DSC from the 2^nd^ heating and cooling cycle) and X‐ray diffraction data of the guanidinium chlorides and the clustomesogens.^[a][b]^

Compound	*T*_m_/°C (Δ*H*/kJ mol^−1^) (Cr to LC) ^[c]^	Mesophase	*T*_c_/°C (Δ*H*/kJ mol^−1^) (LC to I) ^[c]^	*d*/Å (expt./calcd.) ^[d]^	Miller indices
**(C_10_)GCl**		Col_h_	94 (−1.0) 97 (0.9)	*T*=80 °C 29.04 17.01 (16.77) 3.9 *a*=33.53	(10) (11) halo
**(C_12_)GCl**	12 (−9.2)	Col_h_	103 (−0.7)	*T*=85 °C 32.44 18.64 (18.72) 16.19 (16.22) 4.6 *a*=37.46	(10) (11) (20) halo
**(C_14_)GCl**	35 (−37.9) 25 (35.4)	Col_h_	86 (2.9) 86 (1.3)	*T*=72 °C 33.11 19.14 (19.11) 16.59 (16.55) 4.5 *a*=38.23	(10) (11) (20) halo
**[(C_10_)G]_2_[Mo_6_Cl_8_Cl_6_]**		SmA	65 (−2.2) 66 (1.7)	*T*=56 °C 33.70 16.84 (16.85) 9.91 4.3	(001) (002) cluster halo
**[(C_12_)G]_2_[Mo_6_Cl_8_Cl_6_]**	42 (*T*_g_) 32 (*T*_g_)	SmA	90 (−2.8) 86 (2.8)	*T*=60 °C 36.34 18.11 (18.17) 9.93 4.5	(001) (002) cluster halo
**[(C_14_)G]_2_[Mo_6_Cl_8_Cl_6_]**	44 (−39.1) 41 (23.1)	SmA	90 (−1.4) 90 (6.2)	*T*=50 °C 38.21 19.34 (19.10) 9.35 4.5	(001) (002) cluster halo
**[(C_10_)G]_2_[Mo_6_Br_8_Cl_6_]**		SmA	100 (−2.5) 100 (3.4)	*T*=76 °C 34.14 17.00 (17.07) 10.38 4.2	(001) (002) cluster halo
**[(C_12_)G]_2_[Mo_6_Br_8_Cl_6_]**	48 (*T*_g_) 38 (*T*_g_)	SmA	98 (−1.3) 99 (1.4)	*T*=76 °C 40.08 20.39 (20.04) 10.28 4.2	(001) (002) cluster halo
**[(C_14_)G]_2_[Mo_6_Br_8_Cl_6_]**	40 (−45.4) 38 (40.0)	SmA	92 (POM) 90 (POM)	*T*=80 °C 40.15 20.92 (20.07) 10.64 4.5	(001) (002) cluster halo
**[(C_10_)G]_2_[Mo_6_I_8_(C_2_F_5_CO_2_)_6_]**		SmA	59 (POM) 59 (POM)	*T*=56 °C 29.67 17.12 (14.83) 13.98 3.8	(001) (002) cluster halo
**[(C_12_)G]_2_[Mo_6_I_8_(C_2_F_5_CO_2_)_6_]**		SmA	62 (POM) 62 (POM)	*T*=56 °C 34.74 13.78 3.9	(001) cluster halo
**[(C_14_)G]_2_[Mo_6_I_8_(C_2_F_5_CO_2_)_6_]**	11 (−39.3) 2 (28.3)	SmA	74 (POM) 74 (POM)	*T*=40 °C 31.27 15.53 (15.64) 13.66 4.2	(001) (002) cluster halo

[a] Phase transitions and enthalpies were determined by DSC from the onset temperatures. Heating/cooling rate: 10 K/min. [b] The following phases were observed: Cr crystalline, G glassy phase, SmA smectic A, Col_h_ hexagonal columnar, I isotropic. [c] Melting and clearing temperature (*T*_m_, *T*_c_) were determined from 2^nd^ heating (top) and 2^nd^ cooling (bottom) in the DSC. If no clearing point could be observed in the DSC curve, transition temperatures were determined via POM. In some cases, glass transitions (*T*_g_) were detected instead of melting points. [d] For columnar phases, lattice parameter *a* is given.

Under the POM, fan‐shaped textures were observed for guanidinium chlorides **(C_n_)GCl** (n=10, 12, 14) (Supporting Information, Figure S15) suggesting columnar mesophases, while the clustomesogens **[(C_n_)G]_2_[Mo_6_Cl_8_Cl_6_]**, **[(C_n_)G]_2_[Mo_6_Br_8_Cl_6_]** and **[(C_n_)G]_2_[Mo_6_I_8_(C_2_F_5_CO_2_)_6_]** displayed only uncharacteristic textures (Supporting Information, Figures S16–S18). In case of **[(C_10_)G]_2_[Mo_6_I_8_(C_2_F_5_CO_2_)_6_]** homeotropic alignment was found, indicating SmA phases.

The X‐ray diffractogram of **(C_10_)GCl** presents two distinct reflections in the small angle section, which were assigned as (10) and (11) reflections of a Col_h_ phase with *pmmm* symmetry and lattice parameter *a*=33.53 Å (Supporting Information, Figure S18). In the wide‐angle section, a broad halo around 4.5 Å was visible, which is due to the interactions of the molten alkyl side chains. Similar results were observed for the higher homologues **(C_12_)GCl**, **(C_14_)GCl**, which showed an additional (20) reflex in the small angle region and the ratio 1 : 1/√3 : 1/2 of the distances corresponding to the (10), (11) and (20) reflections characteristic for the Col_h_ phase.

**Figure 2:**
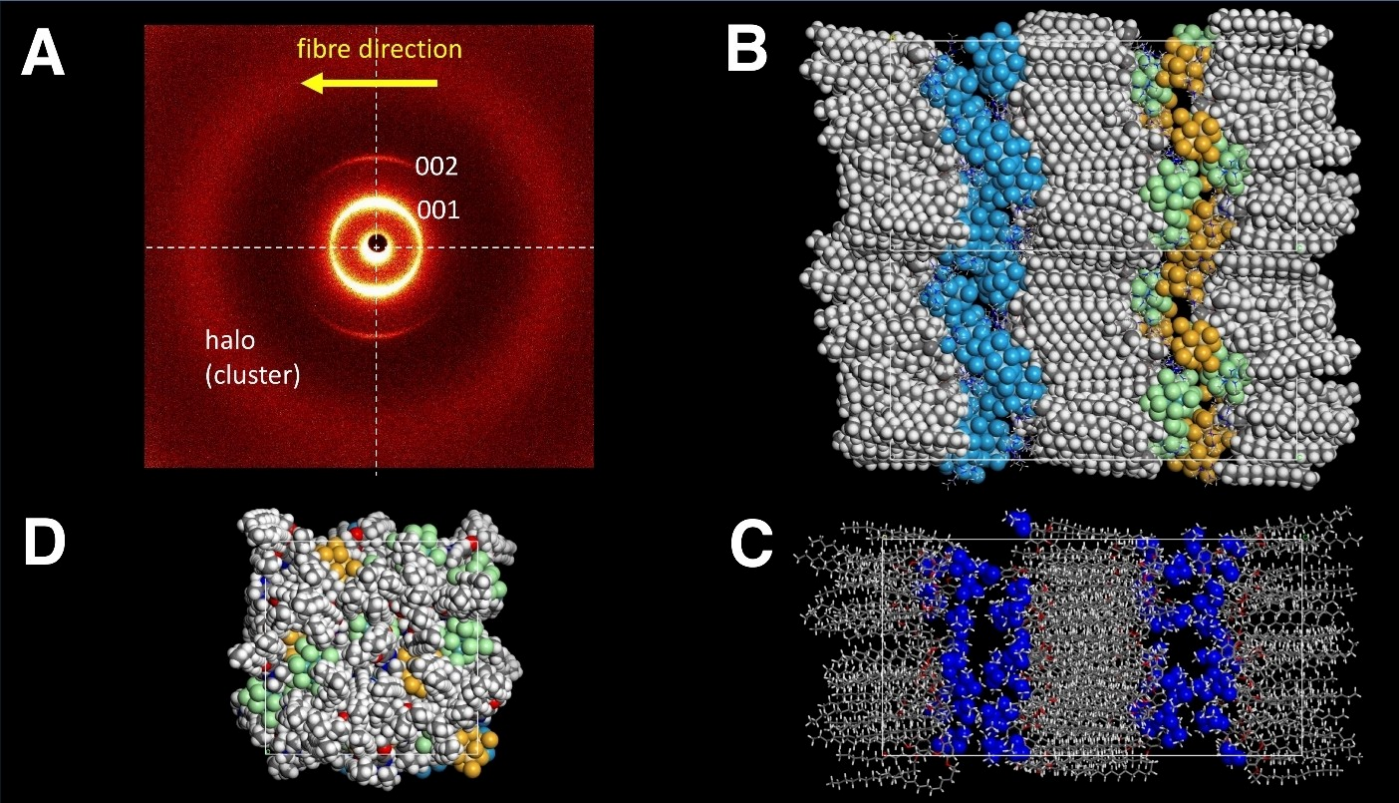
X‐ray pattern of an aligned fibre of **[(C_14_)G]_2_[Mo_6_Cl_8_Cl_6_]** and proposed molecular packing in the undulated smectic A phase for clustomesogens. **A**: SAXS pattern with reflections for the 1D periodic lamellar structure and the halo for the average distance of clusters. **B**: Side view parallel to the layers. Aliphatic chains and the coloured clusters (blue, orange, green) are shown as space‐filling models, while guanidium ions and aromatic units are represented with a simple stick model. **C**: Side view of a stick model in which all nitrogen atoms of the guanidinium ions are highlighted in a space‐filling representation (blue), showing the disorder within the layer. **D**: Top view on a layer looking at the aliphatic chains pointing upwards. This is only half of the chains. The visibility of the below lying clusters demonstrates the complete interdigitation of the chains.

Clustomesogen **[(C_14_)G]_2_[Mo_6_Cl_8_Cl_6_]** displayed two distinct (001) and (002) reflections in the small angle section indicative of the SmA phase (Figure 2 and Supporting Information, Figure S22). In addition, a broad reflection around 9.35 Å due to the average distance of the liquid‐like cluster was visible in the SAXS and a broad halo around 4.5 Å in the wide‐angle section. The layers can be aligned by extrusion of a fibre from the liquid crystal phase. Although the reflections for the layer spacing were fairly well aligned the diffuse cluster signal revealed no preferential direction. This indicates a rather high disorder in the ionic nano space of the layered phase. Related XRD data were obtained for the other clustomesogens.

To rationalize the liquid‐crystalline self‐assembly of the clustomesogens, we generated a packing model for **[(C_14_)G]_2_[Mo_6_Cl_8_Cl_6_]** with the program suite Materials Studio, in which we assemble 4×4 mesogens in a section of a layer and position two layers in the volume of *a*×*b*×*c*=(75.6×38.9×38.9)Å^3^ (Figure 2, for details see Supporting Information). Chart A highlights the side view of two such volumes after geometry optimization. Ionic nano phases are completely nanosegregated from aliphatic areas and the layers seem to be slightly undulated. The clusters are differently coloured in one layer to distinguish individual clusters. Here the high disorder of the clusters in the ionic nanophase is evident. This is further confirmed with chart C, in which the nitrogen atoms of the guanidinium ions are displayed in a space‐filling representation. This disorder and undulations visualize the liquid nature of the ionic nanophase and consequently, rationalize the diffuse scattering with a distance typical for two neighbouring clusters (9.4 Å) in the X‐ray experiment.[^9c^, ^11^] Coulomb interactions result in a complete mixing of positive and negative charges in the polar layer. In contrast, the reflections corresponding to the layer spacing indicate an alignment, also explained by the model showing one‐dimensional positional order of the layers. Eventually, chart D shows the top view on a layer. Only the aliphatic chains directing upwards to the observer are visualized. The cluster's visibility below the aliphatic layer clearly demonstrates that the layers are completely interdigitated, in order to fill the aliphatic space. This is a consequence of the incommensurate size between clusters and minidendrons. The large size of the cluster anion as compared to spherical chloride anion in compounds **(C_n_)GCl** does not allow the typical “pizza‐slice” packing of cationic minidendrons with small spherical counterions resulting in columnar self‐assembly.

In previous studies, only an ammonium cation bearing three decyloxycyanobiphenyl chains (Kat)^+^ was associated with all cluster anions used therein, leading to the formation of nematic clustomesogens.[^2c^, ^6b^] By comparing the liquid‐crystalline behaviour of these previously described hybrids and the one of the present studies, we can assess first that the nature of apical and inner ligands has a stronger impact on the mesophase stabilization when no promesogenic units are integrated in the hybrid. Indeed, the observed LC to I transition temperatures for (Kat)_2_Mo_6_Cl_8_Cl_6_ and (Kat)_2_Mo_6_Br_8_Cl_6_ were in the same range (97–99 °C)^[6b]^ while here a difference of 30 K is observed for C_10_G containing hybrids. This effect is somewhat decreased with increasing alkyl chain lengths for [Mo_6_Cl_8_Cl_6_]^2−^ and [Mo_6_I_8_(OCOC_2_F_5_)_6_]^2−^ clusters. The different behaviour of the [Mo_6_Br_8_Cl_6_]^2−^ cluster could result from the different electrostatic properties. [Mo_6_Br_8_Cl_6_]^2−^ is more polarized than [Mo_6_Cl_8_Cl_6_]^2−^. Thus, stronger interactions (electrostatic, hydrogen bonds) with the organic cation can be expected for [Mo_6_Br_8_Cl_6_]^2−^ than for [Mo_6_Cl_8_Cl_6_]^2−^. Such differences in interaction strength are probably minimized by increasing the terminal alkyl chain length. Moreover, intercluster interactions should be stronger within a cluster's plane in the smectic phase for [Mo_6_Br_8_Cl_6_]^2−^ than for [Mo_6_Cl_8_Cl_6_]^2−^, which could also explain this difference in clearing temperature. As previously observed, clearing temperatures are much lower when [Mo_6_I_8_(OCOC_2_F_5_)_6_]^2−^ is used instead of the two other ions.

Therefore, the mesophase stability does not only depend on weak interactions between the interdigitated organic aliphatic chains but relies also greatly on the electrostatic interactions within the polar layers. The choice of the anion is not only crucial to tailor the emission properties of the hybrid but also influences significantly the mesophase stability.

### Linear Optical Properties of Clustomesogens

**Figure 3:**
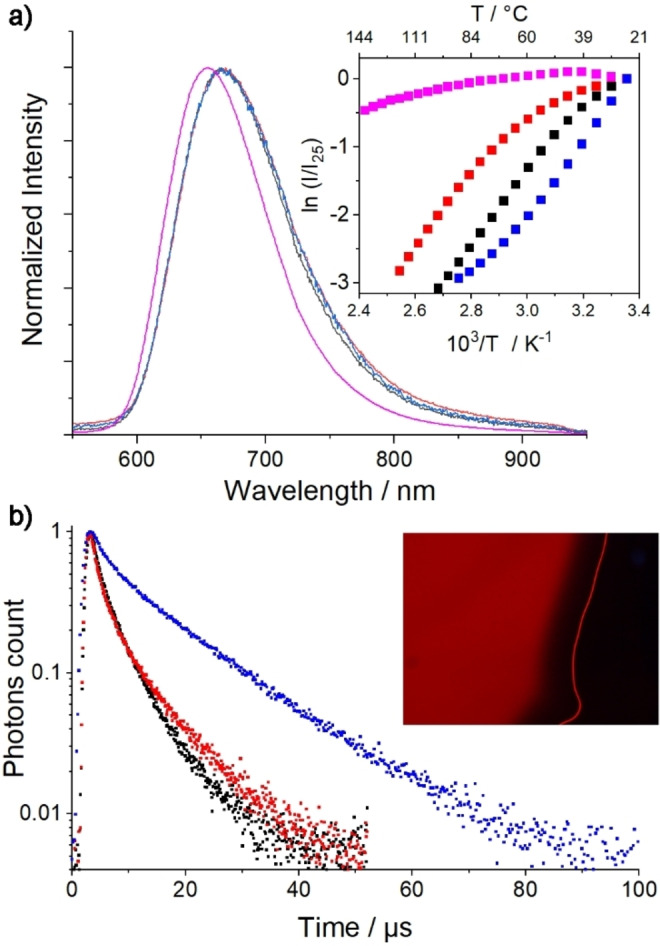
a) Emission spectra of Cs_2_Mo_6_I_8_(OCOC_2_F_5_)_6_ (magenta) and the respective hybrids with **C_10_G** (black), **C_12_G** (red), **C_14_G** (blue) counter cations; inset: follow up of emission vs. temperature; b) emission decay profiles recorded at 23 °C for **[C_n_G]_2_[Mo_6_I_8_(OCOC_2_F_5_)_6_]**: n=10 (black), 12 (red) and 14 (blue); inset: picture of **[C_12_G]_2_[Mo_6_I_8_(OCOC_2_F_5_)_6_]** taken under UV‐A irradiation at 40 °C.

Clustomesogen emission properties were first investigated by steady state experiments. Excitation vs. emission maps (see Supporting Information, Figure S29–S32) show that the optimal excitation wavelength, to observe the broad and structureless emission signal of metal clusters in the red‐NIR, ranges from 350 nm up to 425 nm. Associating the cluster anion with the organic cations does not modify the envelop of their emission spectrum (see Supporting Information, Figure S33). Only a small red shift of about 13 nm of the emission spectrum maximum is observed for compounds containing the [Mo_6_I_8_(OCOC_2_F_5_)_6_]^2−^ cluster anion (Figure 3a). For hybrids containing the two other cluster anions, no significant changes in the emission maximum position were observed. However, associating the organic minidendrons with cluster anions does influence the ability of cluster to emit as revealed by the absolute quantum yield values calculated for each compound (Table 2) that drop down drastically compared to pristine powders. In fact, it is well known that metal cluster anion emission is efficiently quenched by molecular oxygen.^[11]^ Hence, the ability of the cluster triplet excited state to emit a photon when coming back to its ground state instead of transferring its energy by collisional quenching to the ground triplet state of molecular oxygen relies on the oxygen permeability of the hybrids that depends strongly on the material nanostructuration. Obviously, it depends also on the atmosphere the sample is placed in: For instance, saturating the atmosphere of **[(C_10_)G)_2_][Mo_6_I_8_(OCOC_2_F_5_)_6_]** with N_2_ induces a large increase of its AQY from 1.4 % to 25.7 % while the powdered Cs salt precursor AQY value in air is around 35 %. Remarkably, AQY values calculated in N_2_ saturated atmosphere for hybrids do not compete with the AQY calculated for the pristine powders. This might be because saturating the atmosphere of the integrating sphere by blowing N_2_ does not remove completely the molecular oxygen trapped within the material.

**Table 2:** Photophysical data of starting cluster compounds and related hybrids.

	λ_max_	Φ in Air/N_2_	Kinetic parameters		τ_av_/μs
			τ_1/_μs (%)	τ_2/_μs (%)	
Na_2_Mo_6_Br_8_Cl_6_	726	23.8/‐	35 (0.44)	104.9 (0.56)	90.5
[(C_10_)G]_2_[Mo_6_Br_8_Cl_6_]	726	2.4/5.5	16.1 (0.82)	33.1 (0.18)	21.4
[(C_12_)G]_2_[Mo_6_Br_8_Cl_6_]	726	4.7/11	18.9 (0.66)	51.8 (0.34)	38.2
[(C_14_)G]_2_ [Mo_6_Br_8_Cl_6_]	726	6.2/17	21.3 (0.36)	54.0 (0.64)	48.2
					
Na_2_Mo_6_Cl_14_	728	43.4/‐	53.3 (0.08)	173 (0.92)	169.8
[(C_10_)G]_2_[Mo_6_Cl_8_Cl_6_]	732	4.7/11.2	21.4 (0.74)	51.7 (0.26)	35.3
[(C_12_)G]_2_[Mo_6_Cl_8_Cl_6_]	732	4.0/16.4	24.5 (0.83)	75.5 (0.17)	44.2
[(C_14_)G]_2_[Mo_6_Cl_8_Cl_6_]	745	7.0/11.4	33.4 (0.55)	81.5 (0.45)	65.6
					
Cs_2_Mo_6_I_8_(OCOC_2_F_5_)_6_	654	35/‐	40.8 (0.32)	135 (0.68)	123
[(C_10_)G]_2_[Mo_6_I_8_(OCOC_2_F_5_)_6_]	667	1.4/25.7	1.54 (0.62)	5.65 (0.38)	4.4
[(C_12_)G]_2_[Mo_6_I_8_(OCOC_2_F_5_)_6_]	667	1.6/23.7	1.79 (0.70)	8.2 (0.30)	6.0
[(C_14_)G]_2_[Mo_6_I_8_(OCOC_2_F_5_)_6_]	667	2.6/18.3	2.4 (0.49)	14.4 (0.51)	12.8

*I*(*t*)=Σa_i_exp(‐*t*/τ_i_); Average lifetime is calculated as τ_av_=(Σa_i_τ_i_^2^)/(Σa_i_τ_i_).

Temperature‐dependent emission measurements were realized on cooling from the isotropic state for the most emissive samples: i.e., hybrids with [Mo_6_I_8_(OCOC_2_F_5_)_6_]^2−^ and are compared to the pristine powder behaviour (inset Figure 3a). Only a little increase of the emission signal is observed upon cooling for the cluster caesium salt, while for all corresponding hybrids the increase is about more than one order of magnitude between the isotropic state and 25 °C. This is ascribed to a lowering of the hybrid oxygen permeability induced by the nanostructuration within the mesophase (see Supporting Information, Figures S34–S37 for emission spectra evolution with *T*).

Phosphorescence lifetime emission decays were investigated at 23 °C in the solid, LC or glassy states for hybrids and precursors (see Supporting Information, Figures S38–S50). In all cases, the recorded emission decay profiles were fitted to a two‐exponential decay and the goodness‐of‐fit judged by the χ^2^ values (0.99–1.02) and the residual plot distribution. The average lifetime values (τ_av_) calculated for the pristine powder and related hybrids give a better view of the hybrid's O_2_ sensitivity, in particular for compounds containing the [Mo_6_I_8_(OCOC_2_F_5_)_6_]^2−^ anion that, as stated previously, is one of the most sensitive cluster anions toward the presence of O_2_ (see Supporting Information, Figures S51, S52 for further details). As a Cs salt, its τ_av_ value reaches 123 μs while in the LC hybrids, this value drops down to 4.4 μs for n=10 and increases up to 12.8 μs when n=14 (Figure 3b)). Such decrease is in line with calculated AQY values. The same trend is observed for hybrids containing the [Mo_6_Cl_8_Cl_6_]^2−^ and [Mo_6_Br_8_Cl_6_]^2−^ anions. According to these measurements, it seems that cluster anions are better protected from collisional quenching with O_2_ when the alkyl tail size increases. This observation could be correlated to the lattice parameter evolution in the LC phase that is not significantly modified when passing from n=12 to n=14 which implies a denser packing of C_14_ alkyl chains.

## Conclusion

In conclusion, we present herein the first clustomesogens obtained by electrostatic association of cluster anions with amphiphilic minidendron cations. All hybrid compounds self‐assemble in smectic A mesophases in contrast to the columnar chloride salts of guanidinium derivatives. Single crystal diffraction studies of methoxy derivatives evidence hydrogen bonds between the guanidinium proton and the apical ligands of cluster anions. In addition to Coulomb interactions, such interactions might be of importance in the self‐organization process of clustomesogens, in particular in the stabilization of the mesophase. Emission studies realized in the steady state show that associating the cluster anion with the organic dendrons has little influence on the emission maximum intensity position. However, temperature‐dependent emission studies emphasize that the emission intensity drops down dramatically in hybrids compared to the pristine powder. Such phenomenon is interpreted as an increase of hybrid O_2_ permeability with temperature that is correlated to the hybrid self‐assembling abilities. Time correlated emission experiments are in line with quantum yield calculations and evidence for the first time the influence of the alkyl chain length on the ability of O_2_ to quench the cluster anion emission. In all cases, collisional quenching is better prevented for longer alkyl chains.

## Experimental Section

Full details of synthetic procedures and characterization data, NMR spectra, X‐ray crystal structure analyses, differential scanning calorimetry (DSC) data, polarizing optical microscopy (POM) data, X‐ray diffraction (XRD) data, photoluminescence data as well as the proposed packing models can be found in the Supporting Information.

Deposition Numbers CCDC 2093021 (for **(C**_**1**_ **)GuaHCl**), 2093022 (for **[(C**_**1**_ **)GuaH]**_**2**_ **[Mo**_**6**_ **I**_**8**_ **(C**_**2**_ **F**_**5**_ **CO**_**2**_ **)**_**6**_ **]**), 2093023 (for **[(C**_**1**_ **)GuaH]**_**2**_ **[Mo**_**6**_ **Br**_**8**_ **Cl**_**6**_ **]**), 2093024 (for **[(C**_**1**_ **)GuaH]**_**2**_ **[Mo**_**6**_ **Cl**_**8**_ **Cl**_**6**_ **]** ⋅ 4 CH_2_Cl_2_) contain the supplementary crystallographic data for this paper. These data are provided free of charge by the joint Cambridge Crystallographic Data Centre and Fachinformationszentrum Karlsruhe Access Structures service.

## Author Contributions

The manuscript was written through contributions of all authors, who have given approval to the final version of the manuscript. M.E. designed, synthesized, and characterized all guanidinium compounds and clustomesogens, and performed POM, DSC, and XRD analysis, I.C. performed luminescence measurements, and proofread the manuscript. N.D. synthesized and characterized inorganic cluster compounds. W. F. performed the X‐ray single‐crystal structure analyses. A.B. prepared the schemes and proofread the manuscript. A.Z. checked experimental data, prepared figures, and proofread the manuscript. M.L. provided his expertise on XRD of LCs, performed the Materials Studio calculations, wrote, and proofread the manuscript. G.T. performed emission/lifetime/quantum yield measurements. S.C. performed single crystal X‐ray analyses and proofread the manuscript. E.J. provided his expertise on the photophysical measurements. Y.M. supervised, coordinated the research, performed emission, lifetime, and quantum yield measurements, wrote, and proofread the manuscript. S.L. coordinated the research, wrote, and proofread the manuscript.

## Conflict of interest

The authors declare no conflict of interest.

## Supporting information

As a service to our authors and readers, this journal provides supporting information supplied by the authors. Such materials are peer reviewed and may be re‐organized for online delivery, but are not copy‐edited or typeset. Technical support issues arising from supporting information (other than missing files) should be addressed to the authors.

Supporting Information
