# Supplementary material for: Joint Venture of Metal Cluster and Amphiphilic Cationic Minidendron Resulting in Near Infrared Emissive Lamellar Ionic Liquid Crystals
Source: Chemistry. 2021 Nov 29;28(3):e202103446. doi: 10.1002/chem.202103446 (PMC9300031; doi:10.1002/chem.202103446)
Supplement: Supplementary file 1 — Supporting Information [file CHEM-28-0-s001.pdf]

Chemistry–A European Journal

Supporting Information

Joint Venture of Metal Cluster and Amphiphilic Cationic Minidendron Resulting in Near Infrared Emissive Lamellar Ionic Liquid Crystals

Max Ebert, Irene Carrasco, Noée Dumait, Wolfgang Frey, Angelika Baro, Anna Zens, Matthias Lehmann,* Gregory Taupier, Stephane Cordier, Emmanuel Jacques, Yann Molard,* and Sabine Laschat*

Table of Contents

1) General Methods.. S2

2) Experimental Procedures…... S3

3) X-Ray Crystal Structure Analyses.. S10

4) Differential Scanning Calorimetry (DSC) Data.. S17

5) Polarizing Optical Microscopy (POM) Data.. S29

6) X-Ray Diffraction (XRD) Data.. S31

7) Photoluminescence Data.. S41

8) Proposed Packing Models.. S52

9) NMR Data... S54

10) References.. S63

1) General Methods

NMR spectra were recorded on 300, 400, 500 or 700 MHz spectrometers at room temperature. Infrared spectra were recorded on a Fourier transform infrared (FT-IR) spectrometer with Platinum ATR system at room temperature. Mass spectra (MS) and high-resolution mass spectra (HRMS) were recorded using the ESI-TOF technique. For POM analysis an *Olympus* BX50 microscope (heat unit: *Linkam* BX50) was used. For DSC measurements, the samples were placed in *Mettler Toledo* aluminum pans (40 µL). Recording of the respective thermograms was performed on a *Mettler Toledo* DSC 822e calorimeter. X-ray diffraction in the mesophase was performed using a *Bruker* Nanostar C ($Cu_{K\alpha}$: $\lambda = 1.5406$ Å) with an equipped HI-STAR detector. The limit from the instrument is 2Theta ≈ 25°. Single crystal analysis was conducted on a *Bruker* kappa APEXII Duo diffractometer. Data collection: APEX2 Software Suite; cell refinement: SAINT (both Bruker 2008). The structures were solved by using the program SHELXS 97 (Sheldrick 2008) and refined by using the program SHELXL 97 (Sheldrick 2008). Molecular graphics: XP in SHELXTL-Plus (Sheldrick 2008). Thin-layer chromatography was performed on silica gel 60 F_{254} precoated aluminium plates. Column chromatography was carried out using silica gel (grain size of 40–63 µm) with solvents distilled prior to use. Commercially available reagents were used as purchased unless otherwise stated. Solvents were distilled prior to use or purified and dried by standard procedures. Reactions under Schlenk conditions were performed in dried glassware under inert gas atmosphere.

Excitation vs emission maps were performed with a Horiba Duetta spectrophotometer. Temperature-dependent emission experiments were performed with a Nikon 80i polarized microscope equipped with a Linkam LTS420 hot stage, a Nikon Intensilight C-HGFI (UV 1 filter, 350 nm < λ_{exc} < 380 nm) irradiation source, a Nikon DS-FI2 digital camera and an ocean optics QE65000 photodetector connected by optical fiber. The absolute quantum yields in the solid state were measured with a C9920–03 Hamamatsu system made of a 150 W xenon lamp, a monochromator, an integrating sphere and a red-NIR sensitive PMA-12 detector. Lifetime measurements were realized using a picosecond laser diode (Jobin Yvon deltadiode, 375 nm) and a Hamamatsu C10910-25 streak camera mounted with a slow single sweep unit. Signals were integrated on a 30 nm bandwidth. Fits were obtained using origin software and the goodness of fit judge by the reduced $\chi 2$ value and residual plot shape.

2) Experimental Procedures

General Procedure for the Synthesis of 1,2,3-Trisalkoxybenzenes (2) (GP 1)

Pyrogallol (2.00 g, 15.9 mmol. 1 eq.), NaOH (2.54 g, 63.4 mmol, 4 eq.) and the respective 1-bromoalkane (52.3 mmol, 3.3 eq.) were added to degassed DMF (150 ml) under inert atmosphere. The mixture was stirred at r.t. for 18 h. The mixture was poured on ice (100 mL) and the precipitate was filtered off (for chain lengths ≥12) or the mixture was extracted with hexanes (3 × 60 mL) (for C_{10} chain). The resulting crude product was recrystallized from acetone (longer chains) or purified by flash chromatography (hexanes, SiO_2).

1,2,3-Tris(decyloxy)benzene (2a)

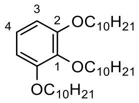

According to GP1; yield: 7.55 g (13.8 mmol, 87%), colorless liquid. ^{1}H-NMR (400 MHz, $CDCl_3$) δ = 6.90 (1H, t, *J* = 8.3 Hz, 4-H), 6.54 (2H, d, *J* = 8.3 Hz, 3-H), 4.00–3.94 (6H, m, OCH_2), 1.88–1.67 (6H, m, OCH_2CH_2), 1.53–1.21 (42H, m), 0.89 (9H, t, *J* = 6.7 Hz, CH_3) ppm; ^{13}C-NMR (101 MHz, $CDCl_3$): δ = 153.5 (C-2), 138.5 (C-1), 123.1 (C-4), 106.9 (C-3), 73.4 (OCH_2), 69.1 (OCH_2), 32.0, 31.9, 30.4, 29.8, 29.70, 29.67, 29.64, 29.61, 29.47, 29.45, 29.42, 29.37, 26.2, 26.1, 22.71, 22.70 (CH_2), 14.1 (CH_3) ppm; Spectroscopic data were in accordance with ref. [1].

1,2,3-Tris(dodecyloxy)benzene (2b)

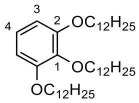

According to GP1; yield: 9.31 g (14.8 mmol, 93%), colorless solid. ^{1}H-NMR (400 MHz, $CDCl_3$) δ = 6.92 (1H, t, *J* = 8.3 Hz, 4-H), 6.56 (2H, d, *J* = 8.3 Hz, 3-H), 4.04–3.93 (6H, m, OCH_2), 1.88–1.73 (6H, m, OCH_2CH_2), 1.55–1.22 (54H, m, CH_2), 0.91 (9H, t, *J* = 6.7 Hz, CH_3) ppm;.^{13}C-NMR (101 MHz, $CDCl_3$): δ = 153.5 (C-2), 138.5 (C-1), 123.1 (C-4), 106.9 (C-3), 73.4 (OCH_2), 69.1 (OCH_2), 32.0, 31.9, 30.4, 29.78, 29.76, 29.72, 29.67, 29.66, 29.63, 29.56, 29.48, 29.45, 29.41, 29.38, 29.35, 26.2, 26.1, 22.7 (CH_2), 14.1 (CH_3) ppm;. Spectroscopic data were in accordance with ref. [1].

1,2,3-Tris(tetradecyloxy)benzene (2c)

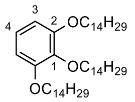

According to GP1; yield: 10.3 g (14.4 mmol, 91%), colorless solid. ^{1}H-NMR (400 MHz, $CDCl_3$) δ = 6.90 (1H, t, *J* = 8.3 Hz, 4-H), 6.54 (2H, d, *J* = 8.3 Hz, 3-H), 4.00–3.91 (6H, m,

OCH_2), 1.84–1.70 (6H, m, OCH_2CH_2), 1.52–1.19 (66H, m, CH_2), 0.88 (9H, t, J = 6.7 Hz, CH_3) ppm; ^{13}C-NMR (101 MHz, $CDCl_3$): δ = 153.5 (C-2), 138.5 (C-1), 123.1 (C-4), 106.9 (C-3), 73.4 (OCH_2), 69.1 (OCH_2), 31.9, 30.4, 29.77, 29.76, 29.72, 29.68, 29.66, 29.47, 29.44, 29.39, 29.38, 26.2, 26.1, 22.7 (CH_2), 14.1 (CH_3) ppm; Spectroscopic data were in accordance with ref. [1].

General Procedure for the Synthesis of 3,4,5-Trisalkoxy-1-nitrobenzenes (3) (GP2)

The respective benzene **2** (2.80 mmol) was dissolved in CH_2Cl_2 (40 mL) and HNO_3 on SiO_2 (17 g) was added. The mixture was stirred at room temperature for 30 min. After filtration through Celite® and removal of the solvent, the pure product was obtained.

HNO_3 on SiO_2 was prepared according to literature.[2]

5-Nitro-1,2,3-tri(decyloxy)benzene (3a)

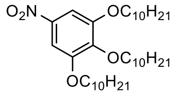

According to GP2; yield: 1.56 g (2.63 mmol, 94%), yellow solid. ^{1}H-NMR (400 MHz, $CDCl_3$): δ = 7.47 (2H, s, ArH), 4.10–3.97 (6H, m, OCH_2), 1.88–1.69 (6H,m, OCH_2CH_2), 1.53–1.22 (42H, m, CH_2), 0.88 (9H, t, J = 6.6 Hz, CH_3) ppm;.^{13}C-NMR (101 MHz, $CDCl_3$): δ = 152.7 (*m*-C), 143.9 (*i*-C), 143.2 (*p*-C), 102.2 (*o*-C), 73.8, 69.5 (OCH_2), 31.94, 31.91, 30.3, 29.71, 29.66, 29.60, 29.57, 29.51, 29.38, 29.34, 29.1, 26.02, 25.99, 22.70, 22.68 (CH_2), 14.1 (CH_3) ppm. Spectroscopic data were in accordance with ref. [1].

5-Nitro-1,2,3-tri(dodecyloxy)benzene (3b)

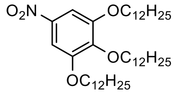

According to GP2; yield: 1.84 g (2.72 mmol, 97%), yellow solid. ^{1}H-NMR (400 MHz, $CDCl_3$) δ = 7.47 (2H, s, Ar-H), 4.10–3.95 (6H, m, OCH_2), 1.86–1.67 (6H, m, OCH_2CH_2), 1.52–1.15 (66H, m, CH_2), 0.88 (9H, t, J = 6.6 Hz, CH_3) ppm; ^{13}C-NMR (101 MHz, $CDCl_3$): δ = 152.7 (*m*-C), 143.9 (*i*-C), 143.2 (*p*-C), 102.2 (*o*-C), 73.8, 69.5 (OCH_2), 31.9, 30.3, 29.75, 29.72, 29.69, 29.67, 29.62, 29.52, 29.44, 29.37, 29.35, 29.1, 26.03, 25.99, 22.7 (CH_2), 14.1 (CH_3) ppm;. Spectroscopic data were in accordance with ref. [1].

5-Nitro-1,2,3-tri(tetradecyloxy)benzene (3c)

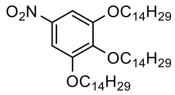

According to GP2; yield: 2.04 g (2.68 mmol, 96%), yellow solid. ^{1}H-NMR (400 MHz, $CDCl_3$) δ = 7.47 (2H, s, Ar-H), 4.10–3.95 (6H, m, OCH_2), 1.86–1.67 (6H, m, OCH_2CH_2), 1.52–1.15

(66H, m, CH_2), 0.88 (9H, t, J = 6.6 Hz, CH_3) ppm; ^{13}C-NMR (101 MHz, $CDCl_3$): δ = 152.7 (*m*-C), 143.9 (*i*-C), 143.2 (*p*-C), 102.2 (*o*-C), 73.8, 69.5 (OCH_2), 31.9, 30.3, 29.75, 29.72, 29.69, 29.67, 29.62, 29.52, 29.44, 29.37, 29.35, 29.1, 26.03, 25.99, 22.7 (CH_2), 14.1 (CH_3) ppm; Spectroscopic data were in accordance with ref. [1].

5-Nitro-1,2,3-trimethoxybenzene (3d)

Acetic acid (40 mL) and concentrated nitric acid (20 mL) were mixed and cooled to 0 °C. 3,4,5-Trimethoxybenzoic acid (10.0 g, 47.1 mmol) was added in small portions under stirring. After complete addition, the mixture was allowed to warm to room temperature overnight. After pouring onto ice (200 mL), the resulting light-yellow precipitate was filtered off and washed with water and dried to give 3d (6.64 g, 31.1 mmol, 66%). ^{1}H-NMR (400 MHz, $CDCl_3$) δ = 7.51 (2H, s, ArH), 3.96–3.92 (9H, m, OCH_3) ppm; ^{13}C-NMR (101 MHz, $CDCl_3$): δ = 152.9 (*m*-C), 143.8 (*p*-C), 143.4 (*i*-C), 101.4 (*o*-C), 61.2 (*p*-OCH_3), 56.5 (*m*-OCH_3) ppm; Spectroscopic data were in accordance with ref. [3].

General Procedure for the Synthesis of 3,4,5-Trialkoxyanilines (4) (GP3)

The respective nitrobenzene **3** (2.50 mmol, 1 eq.) was placed in a flask with EtOH (80 mL), hydrazine hydrate (0.8 mL, 751 mg, 15.0 mmol, 1 eq.) and charcoal (1.50 g). The mixture was stirred under inert atmosphere at reflux for 18 h. After cooling down to room temperature, the mixture was filtered through Celite® and washed with CH_2Cl_2. After removal of the solvent, the crude product was washed with MeOH to obtain the pure product.

3,4,5-Tris(decyloxy)aniline (4a)

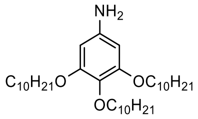

According to GP3; yield: 1.32 g (2.35 mmol, 94%), off-white solid. ^{1}H-NMR (400 MHz, $CDCl_3$): δ = 5.91 (2H, s, ArH), 3.90 (4H, t, J = 6.5 Hz, *m*-OCH_2), 3.84 (2H, t, J = 6.5 Hz, *p*-OCH_2), 2.17 (2H, br s, NH_2), 1.82–1.66 (6H, m, OCH_2CH_2), 1.50–1.20 (42H, m, CH_2), 0.88 (9H, t, J = 6.7 Hz, CH_3) ppm; ^{13}C-NMR (101 MHz, $CDCl_3$): δ = 153.7 (*m*-C), 142.3 (*i*-C), 131.2 (*p*-C), 94.6 (*o*-C), 73.6 (*p*-OCH_2), 69.0 (*m*-OCH_2), 31.95, 31.92, 31.90, 30.3, 29.76, 29.69, 29.66, 29.62, 29.60, 29.56, 29.44, 29.41, 29.36, 29.32, 26.2, 26.1, 22.71, 22.69 (CH_2), 14.1 (CH_3) ppm. Spectroscopic data were in accordance with ref. [1].

3,4,5-Tris(dodecyloxy)aniline (4b)

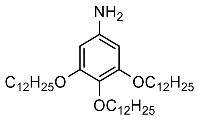

According to GP3; yield: 1.55 g, 2.40 mmol, 96%, off-white solid.
^{1}H-NMR (400 MHz, $CDCl_3$): δ = 5.91 (2H, s, ArH), 3.98–3.80 (6H, m, OCH_2), 3.39 (2H, br s, NH_2), 1.85–1.65 (6H, m, OCH_2CH_2), 1.53–1.17 (54H, m, CH_2), 0.88 (9H, t, J = 6.7 Hz, CH_3) ppm; ^{13}C-NMR (101 MHz, $CDCl_3$): δ = 153.7 (*m*-C), 142.2 (*i*-C), 131.2 (*p*-C), 94.6 (*o*-C), 73.6 (*p*-OCH_2), 69.0 (*m*-OCH_2), 31.96, 31.94, 30.3, 29.78, 29.76, 29.72, 29.67, 29.66, 29.45, 29.41, 29.38, 26.2, 26.1, 22.7 (CH_2), 14.1 (CH_3) ppm. Spectroscopic data were in accordance with ref. [1].

3,4,5-Tris(tetradecyloxy)aniline (4c)

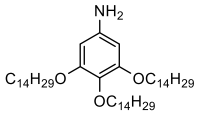

According to GP3, yield: 1.79 g (2.45 mmol, 98%), off-white solid. ^{1}H-NMR (400 MHz, $CDCl_3$): δ = 5.91 (2H, s, ArH), 3.98–3.80 (6H, m, OCH_2), 3.39 (2H, br s, NH_2), 1.85–1.65 (6H, m, OCH_2CH_2), 1.53–1.17 (54H, m, CH_2), 0.88 (9H, t, J = 6.7 Hz, CH_3) ppm; ^{13}C-NMR (101 MHz, $CDCl_3$): δ = 153.7 (*m*-C), 142.2 (*i*-C), 131.2 (*p*-C), 94.6 (*o*-C), 73.6 (*p*-OCH_2), 69.0 (*m*-OCH_2), 31.96, 31.94, 30.3, 29.78, 29.76, 29.72, 29.67, 29.66, 29.45, 29.41, 29.38, 26.2, 26.1, 22.7 (CH_2), 14.1 (CH_3) ppm. Spectroscopic data were in accordance with ref. [1].

3,4,5-Trimethoxyaniline (4d)

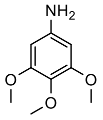

According to GP3; yield: 443 mg, 2.42 mmol, 97%, light yellow solid. ^{1}H-NMR (400 MHz, $CDCl_3$): δ = 5.94 (2H, s, ArH), 3.81 (6H, s, *m*-OCH_3), 3.76 (3H, s, *p*-OCH_3), 3.55 (2H, br s, NH_2) ppm; ^{13}C-NMR (101 MHz, $CDCl_3$): δ = 153.9 (*m*-C), 142.8 (*i*-C), 130.9 (*p*-C), 92.8 (*o*-C), 61.1 (*p*-OCH_3), 55.9 (*m*-OCH_3) ppm. Spectroscopic data were in accordance with ref. [4].

General Procedure for the Synthesis of 1,1,3,3-Tetramethyl-2-(3,4,5-(trialkoxy)phenyl)-guanidinium chlorides (C_n)GCl (GP 4)

The respective aniline **4** (2.00 mmol, 1 eq.) was added to $NaHCO_3$ (1.68 g, 20.0 mmol, 10 eq.) and dry CH_2Cl_2 (40 mL) under inert atmosphere. Tetramethyl chloroformamidinium chloride in abs. CH_2Cl_2 (1 M, 2.2 mL, 2.20 mmol) was added slowly under stirring at r.t. After complete

addition, the mixture was stirred for 1 h at room temperature. The solid was filtered off and washed with CH_2Cl_2. The filtrate was purified by flash column chromatography on HCl-treated silica. The filtrate was placed on the column and washed with EtOAc until the solvent remained colorless. Then the eluent was changed to CH_2Cl_2 / MeOH (10 : 1, *v* / *v*). This fraction was collected separately and concentrated under reduced pressure to obtain the pure product.

1,1,3,3-Tetramethyl-2-(3,4,5-(trisdecyloxy)phenyl)guanidinium chloride (C_{10})GCl

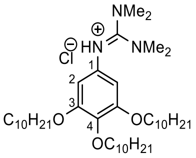

According to GP4; yield: 67%. ^{1}H-NMR (500 MHz, $CDCl_3$): δ = 11.88 (1H, s, NH), 6.36 (2H, s, Ar-H), 3.95–3.87 (6H, m, OCH_2), 2.94 (12H, br s, NMe_2), 1.80–1.67 (6H, m, OCH_2CH_2), 1.48–1.18 (42H, m, CH_2), 0.88 (9H, t, *J* = 6.9 Hz, CH_3) ppm;^{13}C-NMR (126 MHz, $CDCl_3$): δ = 158.7 (*C*(NMe_2)$_2$), 153.9 (C-3), 135.9 (C-4), 133.2 (C-1), 100.3 (C-2), 73.5, 69.5 (OCH_2), 40.6 (br, NMe_2), 32.0, 31.9, 30.3, 29.8, 29.70, 29.65,29.62, 29.60, 29.5, 29.42, 29.39, 29.37 (CH_2), 14.1 (CH_3) ppm; FT-IR: $\tilde{\nu}$ = 3391 (br, w), 2921 (vs), 2853 (s), 1626 (w), 1601 (w), 1559 (w), 1504 (w), 1466 (w), 1430 (w), 1402 (w), 1309 (w), 1232 (w), 1169 (w), 1114 (s), 1068 (w), 1011 (w), 906 (w), 835 (w), 722 (w) cm^{-1}; Anal. calc. for $C_{41}H_{78}ClN_3O_3$ (696.54): C 70.70, H 11.29, N 6.03; calc. for [M·1.45 H_2O]: C 68.14, H 11.28, N 5.81; found: C 68.26, H 11.17, N 5.94.; HRMS (ESI): *m*/*z* calcd. for [M–Cl$^-$]: 660.6038 ($C_{41}H_{78}N_3O_3^+$), found: 660.6037.

1,1,3,3-Tetramethyl-2-(3,4,5-(trisdodecyloxy)phenyl)guanidinium chloride (C_{12})GCl

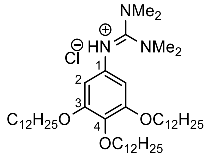

According to GP4; yield: 73% ;^{1}H-NMR (400 MHz, $CDCl_3$): δ = 11.98 (1H, s, NH), 6.39 (2H, s, Ar-H), 4.04–3.84 (6H, m, OCH_2), 3.01 (12H, br s, NMe_2), 1.83–1.66 (6H, m, OCH_2CH_2), 1.52–1.18 (66H, m, CH_2), 0.88 (9H, t, *J* = 6.7 Hz, CH_3) ppm; ^{13}C-NMR (101 MHz, $CDCl_3$): δ = 158.7 (*C*(NMe_2)$_2$), 153.9 (C-3), 136.1 (C-4), 133.2 (C-1), 100.7 (C-2), 73.5, 69.8 (OCH_2), 41.3 (br, NMe_2), 31.9, 30.3, 29.75, 29.72, 29.69, 29.66, 29.62, 29.47, 29.43, 29.37, 26.16, 26.14, 22.7 (CH_2), 14.1 (CH_3) ppm; FT-IR: $\tilde{\nu}$ = 3391 (br, w), 2955 (w), 2915 (vs), 2849 (vs), 2181 (w), 1625 (s), 1599 (s), 1559 (w), 1504 (w), 1467 (s), 1429 (s), 1402 (w), 1309 (w), 1232 (s), 1115 (s), 1068 (w), 1010 (w), 928 (w), 908 (w), 833 (w), 722 (s), 640 (w) cm^{-1}. Anal. calc. for $C_{47}H_{90}ClN_3O_3$ (780.71): C 72.31, H 11.62, N 5.38; found: C 72.08, H 11.73, N 5.44.; HRMS (ESI): *m*/*z* calcd. for [M–Cl$^-$]: 744.6977 ($C_{47}H_{90}N_3O_3^+$), found: 744.6957.

1,1,3,3-Tetramethyl-2-(3,4,5-(tristetradecyloxy)phenyl)guanidinium chloride (C_{14})GCl

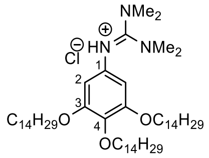

According to GP4; yield: 77%. ^{1}H-NMR (400 MHz, $CDCl_3$): δ = 11.98 (1H, s, NH), 6.39 (2H, s, Ar-H), 4.04–3.84 (6H, m, OCH_2), 3.01 (12H, br s, NMe_2), 1.83–1.66 (6H, m, OCH_2CH_2), 1.52–1.18 (66H, m, CH_2), 0.88 (9H, t, *J* = 6.7 Hz, CH_3) ppm; ^{13}C-NMR (101 MHz, $CDCl_3$): δ = 158.7 (*C*(NMe_2)$_2$), 153.9 (C-3), 136.1 (C-4), 133.2 (C-1), 100.7 (C-2), 73.5, 69.8 (OCH_2), 41.3 (br, NMe_2), 31.9, 30.3, 29.75, 29.72, 29.69, 29.66, 29.62, 29.47, 29.43, 29.37, 26.16, 26.14, 22.7 (CH_2), 14.1 (CH_3) ppm; FT-IR: $\tilde{\nu}$ = 3391 (br, w), 2955 (w), 2915 (vs), 2849 (vs), 2181 (w), 1625 (s), 1599 (s), 1559 (w), 1504 (w), 1467 (s), 1429 (s), 1402 (w), 1309 (w), 1232 (s), 1115 (s), 1068 (w), 1010 (w), 928 (w), 908 (w), 833 (w), 722 (s), 640 (w) cm^{-1};.Anal. calc. for $C_{53}H_{102}ClN_3O_3$ (864.87): C 73.60, H 11.89, N 4.86; found: C 73.40, H 11.81, N 4.91.HRMS (ESI): *m*/*z* calcd. for [M–Cl^-]: 828.7916 ($C_{53}H_{102}N_3O_3^+$), found: 828.7919.

1,1,3,3-Tetramethyl-2-(3,4,5-(trimethoxy)phenyl)guanidinium chloride (C_1)GCl

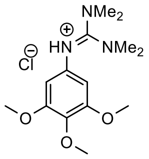

According to GP4; 93%; Melting point: 170 °C (dec.);^{1}H-NMR (300 MHz, $CDCl_3$): δ = 11.65 (1H, s, NH), 6.46 (2H, s, Ar-H), 3.85 (6H, s, *m*-OCH_3), 3.81 (3H, s, *p*-OCH_3), 3.05 (12H, br s, NMe_2) ppm; ^{13}C-NMR (75 MHz, $CDCl_3$): δ = 158.5 (*C*(NMe_2)$_2$), 153.9 (*m*-ArC), 135.6 (*p*-ArC), 133.8 (*i*-ArC), 98.8 (*o*-ArC), 60.9 (*p*-OCH_3), 56.4 (*m*-OCH_3), 40.9, 39.1 (NMe_2).FT-IR: $\tilde{\nu}$ = 2938 (w), 2841 (w), 2253 (w), 2191 (w), 1601 (w), 1560 (w), 1505 (w), 1463 (w), 1417 (w), 1399 (w), 1309 (w), 1233 (w), 1166 (w), 1124 (s), 991 (w), 922 (w), 906 (w), 833 (w), 783 (w), 721 (s), 640 (w) cm^{-1}; HRMS (ESI): *m*/*z* calcd. for [M–Cl^-]: 282.1812 ($C_{14}H_{24}N_3O_3^+$), found: 282.1815.

Synthesis of $Na_2[Mo_6Cl^i_8Cl^a_6]$

$Na_2[Mo_6Cl^i_8Cl^a_6]$ was synthesized using a similar methods as that reported for the synthesis of $Cs_2Mo_6Cl_{14}$.[5] First, large single crystals of $(H_3O)_2Mo_6Cl_{14}\cdot 7\ H_2O$ were prepared following the method reported by Koknat et al.[6] Then, of the latter crystals (2 g; 1.6 mmol) and of NaCl (270 mg; 4.6 mmol, AlfaAesar 99%) were dissolved separately in 40 mL and 27 mL of ethanol (Carlo Erba, 96%) respectively and heated until ebullition. Afterwards, the two solutions were mixed and let under magnetic stirring until dryness. A solution of $Na_2[Mo_6Cl^i_8Cl^a_6]$ was obtained by extraction with dry acetone. Powder of $Na_2[Mo_6Cl^i_8Cl^a_6]$ was then recovered after evaporation of acetone with a rotavapor. It was then stored under dry air.

Yield 95%. EDS analysis: calcd. Na 9%; Mo 27.3%; Cl 63.7%. Exp Na 10%; Mo 28%; Cl 62%.

Synthesis of $Na_2[Mo_6Br^i_8Cl^a_6]$

NaCl (200 mg, 3.4 mmol, AlfaAesar 99%) was poured in a solution of 20 mL of EtOH (Carlo Erba, 96%) and 20 mL aqueous HCl (Sigma-Aldrich, 37%) brought to reflux. Then Mo_6Br_{12} (2 g; 1.3 mmol) was added to the solution until dissolution.[7]

The solution was decanted and filtrated. Afterwards, the solvent was slowly evaporated to precipitate $Na_2[Mo_6Br^i_8Cl^a_6]$.

This precipitate was recovered and dried.

Yield 60%. EDS analysis: calcd. Na 9%; Mo 27.3%; Br 36.4%; Cl 27.3%. Exp Na 9%; Mo 30%; Br 36%; Cl 25%

Synthesis of $Cs_2[Mo_6I^i_8(OCOC_2F_5)^a_6]$

$Cs_2[Mo_6I^i_8(OCOC_2F_5)^a_6]$ was synthesized as previously described with conform analytical data from ref.[9].

Synthesis of the guanidinium/cluster salts $[(C_n)G]_2[Mo_6Q^i_8X^a_6]$

The respective guanidinium chloride **(C_n)GCl** (40 µmol, 2 eq.) was stirred with the respective cluster salt (21 µmol) in MeCN (6 mL) at reflux for 10 min. Both starting compounds are soluble in MeCN. Due to the poor solubility of NaCl and CsCl in MeCN, after a few moments the formation of a white precipitate could be observed. The solids were filtered off and washed with MeCN (4 mL). The filtrate was concentrated under vacuum. The residue was dissolved in CH_2Cl_2 (5 mL) and filtered. After evaporation of the solvent, the pure product was obtained.

3) X-Ray Crystal Structure Analyses

For the guanidinium salts bearing methoxy groups, *i.e.* **(C_1)GCl**, **$[(C_1)G]_2[Mo_6Cl_8Cl_6]$**, **$[(C_1)G]_2[Mo_6Br_8Cl_6]$** and **$[(C_1)G]_2[Mo_6I_8(C_2F_5CO_2)_6]$**, single crystal X-ray structure analyses could be performed. The crystallographic information files have been deposited to the CCDC. The following numbers CCDC 2093021, CCDC 2093022, CCDC 2093023 and CCDC 2093024 were attributed to the structures of **(C_1)GuaHCl**, **$[(C_1)GuaH]_2[Mo_6I_8(C_2F_5CO_2)_6]$**, **$[(C_1)GuaH]_2[Mo_6Br_8Cl_6]$** and **$[(C_1)GuaH]_2[Mo_6Cl_8Cl_6]$**$\cdot$4 CH_2Cl_2, respectively.

The guanidinium chloride **(C_1)GCl** (CCDC 2093021) crystallized with disordered water molecules in a stoichiometric relation of 4 : 3 in the centrosymmetric space group *C*2/*c* (Figure S1).

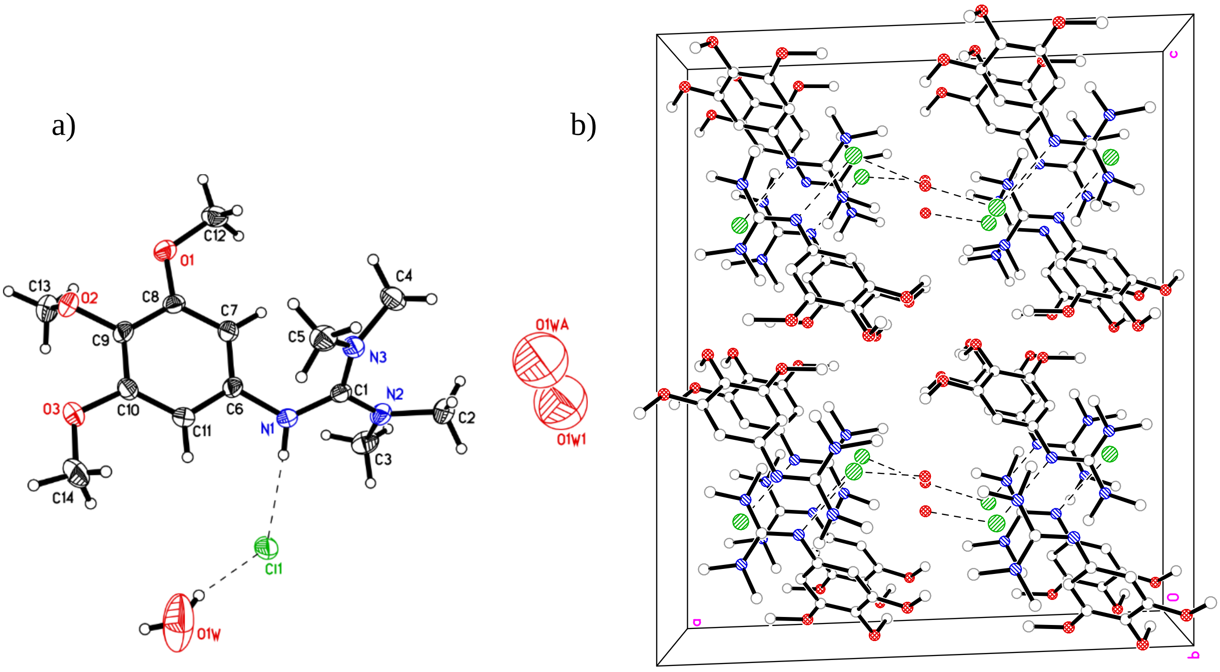

Figure S1: Structures of **(C_1)GCl** in the solid state (a)) and its molecular packing (b)).

The unit cell of the guanidinium chloride **(C_1)GCl** is based on 8 **$(C_1)G^+$** cations, 8 chlorine anions and 6 water molecules. Considering the 8 G^+ and 8 Cl^- anions, the formula is **(C_1)GCl**$\cdot$0.75 H_2O and $Z = 8$. **(C_1)GCl**$\cdot$0.75 H_2O will be denoted in the manuscript **(C_1)GCl** for sake of clarity. It crystallizes in the centrosymmetric space group *C*2/*c* (Figure S1). The oxygen atom of water molecules randomly occupies a 4e and an 8f Wyckoff positions. The N1-H1 function of the guanidinium head is rotated out of plane with respect to the phenyl moiety characterized by the torsion angle H1-N1-C6-C11 of 37(1) degrees. In the meantime, the N1-H1 group acts as donor of a strong intermolecular hydrogen bond where the chloride ion acts as acceptor. The Cl1…. N1 distance is 3.123(2) Å and the H1…Cl1 distance is 2.25(2) Å with

a relevant angle N1-H1…Cl1 of 174(2) °. The methoxy groups on 3- and 5-position of the 3,4,5-methoxy-phenyl moiety have a nearly *equatorial* orientation. In contrast, the 4-methoxy group shows a nearly *axial* orientation. The packing diagram of the cell-plot also shows the stabilization of the structure by a network of hydrogen bonds between the chloride ions and the disordered water positions (Figure S1b).

[(C$_n$)G]$_2$[Mo$_6$Cl$_8$Cl$_6$]·4 CH_2Cl_2 ((CCDC 2093024), denoted **[(C$_n$)G]$_2$[Mo$_6$Cl$_8$Cl$_6$]** in the following for the sake of clarity), **[(C$_n$)G]$_2$[Mo$_6$Br$_8$Cl$_6$]** and **[(C$_1$)G]$_2$[Mo$_6$I$_8$(C$_2$F$_5$CO$_2$)$_6$]** (CCDC 2093022) crystallize all in the *P*-1 space group. The structural analysis confirms an anion/cation ratio of ½. This ratio is fixed by the 2– charge of the cluster unit that is counterbalanced by the charge of two counter cations **[(C$_1$)G]$^+$**. As a result, and as shown in Figure S2, in the solid state, cluster anions lie between two organic cations. **[(C$_n$)G]$_2$[Mo$_6$Cl$_8$Cl$_6$]**·4 CH_2Cl_2, **[(C$_n$)G]$_2$[Mo$_6$Br$_8$Cl$_6$]** and **[(C$_1$)G]$_2$[Mo$_6$I$_8$(C$_2$F$_5$CO$_2$)$_6$]** are all built up from cluster units with the general formula $[Mo_6X_8L_6]^{2-}$ (X = Cl, Br and I ; X = Cl, (C_2F_5COO)). The Mo_6X_8 cluster core is based on a Mo_6 metallic cluster which is face-capped by eight inner ligands. The coordination sphere of each metal is completed by additional L ligands leading to the $[Mo_6X_8L_6]^{2-}$ cluster unit. The analysis of distances and angles are in agreement with those found in previously reported structures built up from this kind of cluster unit ($[Mo_6Cl_8Cl_6]^{2-}$: ref. [5]; $[Mo_6Br_8Cl_6]^{2-}$: ref. [8]; $[Mo_6I_8(C_2F_5CO_2)_6]^{2-}$: ref. [9]).

In the cluster hybrid **[(C$_1$)G]$_2$[Mo$_6$I$_8$(C$_2$F$_5$CO$_2$)$_6$]** (CCDC 2093022), one guanidinium N-H donor forms an intermolecular hydrogen bond to the carbonyl oxygen of a pentafluoro-propionate moiety. The N10…O6 distance is 2.89(1) Å and the H10…O6 distance is 2.11(5) Å. The relevant angle N10-H10...O6 is 157(10) deg. The intramolecular properties of the guanidinium cation are similar as described in the structure of **(C$_1$)GCl**. In the cell plot it is evident, that the anion positions were on the corners of the unit cell. In contrast the cations were organized around the inversion. The residual electron density of the structure is rather high (6.2 e/Å^3), presumably due to the crystal quality, resulting in larger standard deviations of the geometric parameters. The presence of solvent molecules randomly distributed within the structure cannot be fully excluded and could also explain high residual electron density peaks.

a)

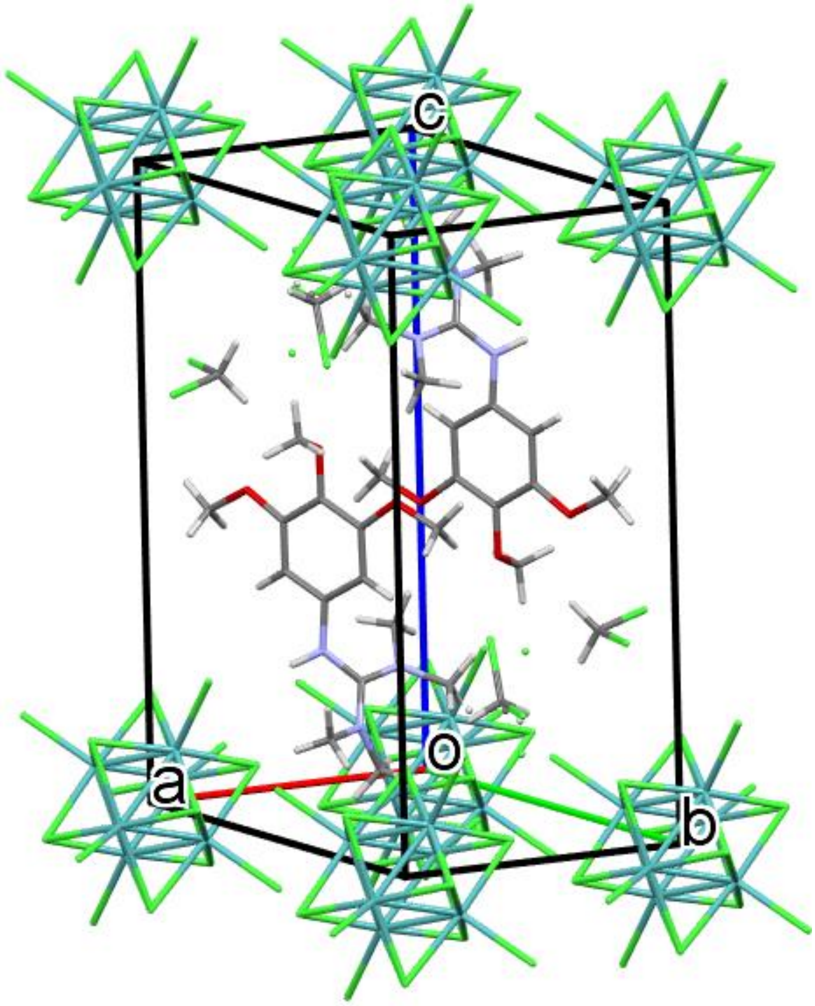

b)

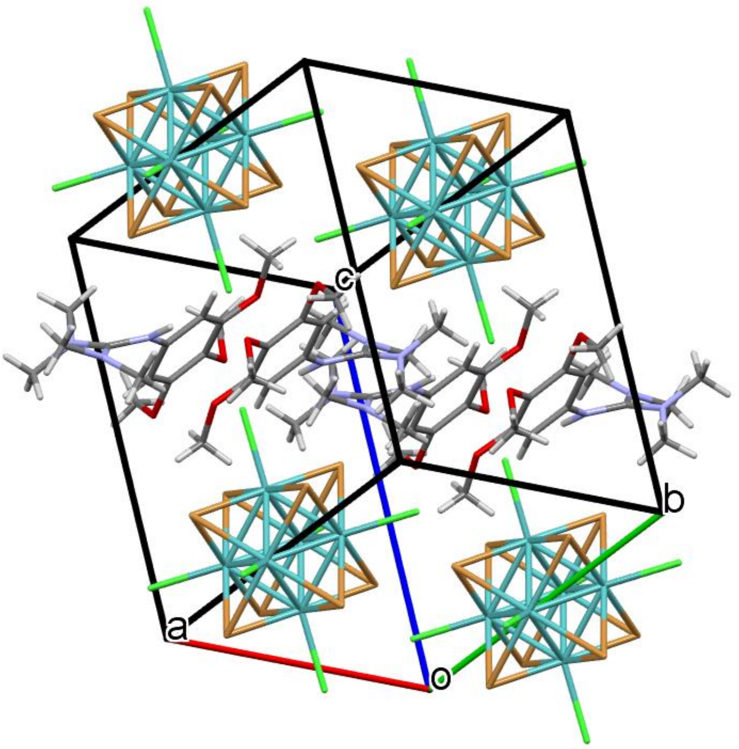

c)

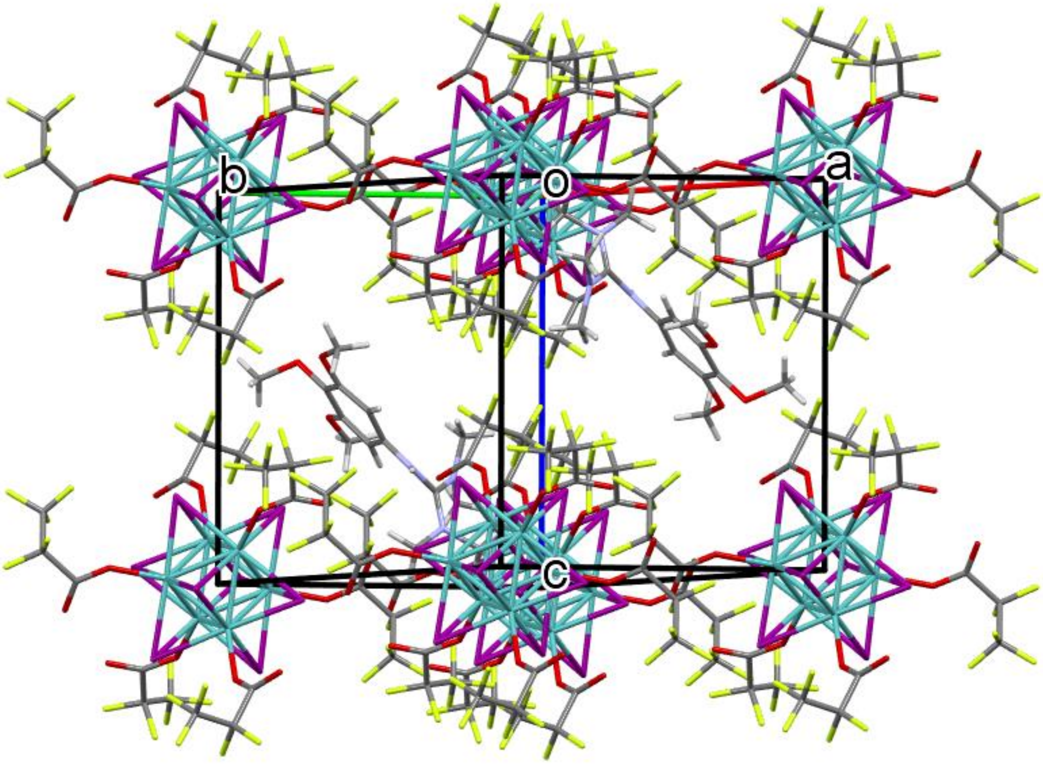

Figure S2: Representations of the structures of a) **$[(C_1)G]_2[Mo_6Cl_8Cl_6]$**$\cdot$4 CH_2Cl_2, b) **$[(C_1)G]_2[Mo_6Br_8Cl_6]$** and c) **$[(C_1)G]_2[Mo_6I_8(C_2F_5CO_2)_6]$**.

Table S1. Crystal data for **$(C_1)GCl$** as well as the cluster hybrids **$[(C_1)G]_2[Mo_6Cl_8Cl_6]\cdot 4\ CH_2Cl_2$, $[(C_1)G]_2[Mo_6Br_8Cl_6]$** and **$[(C_1)G]_2[Mo_6I_8(C_2F_5CO_2)_6]$**

	**$(C_1)GCl$**<br>$Cl(C_{14}H_{24}N_3O_3)$ 0.5 (H_2O)<br>CCDC 2093021	**$[(C_1)G]_2[Mo_6Br_8Cl_6]$**<br>$Br_8Cl_6Mo_6$, $2(C_{14}H_{24}N_3O_3)$<br>CCDC 2093023	**$[(C_1)G]_2[Mo_6I_8(C_2F_5CO_2)_6]$**<br>$Br_8(C_2F_5COO)_6Mo_6$, $2(C_{14}H_{24}N_3O_3)$<br>CCDC 2093022
empirical formula	$C_{14}H_{24.5}ClN_3O_{3.75}$	$C_{24}H_{48}Br_8Cl_6Mo_6N_6O_6$	$C_{46}H_{48}F_{30}I_8Mo_6N_6O_{18}$
formula weight	330.31	1992.34	3133.74
temperature (K)	135(2)	135(2)	135(2)
wavelength (Å)	1.54178	0.71073	0.71073
crystal system	monoclinic	triclinic	triclinic
space group	C2/c	P-1	P-1
unit cell dimensions			
a =	19.2290(9) Å	10.3451(4) Å	12.5110(5) Å
b =	8.0710(5) Å	12.0705(5) Å	12.8037(5) Å
c =	22.5888(11) Å	12.2756(5) Å	13.3006(6) Å
α =	90°	67.418(2)°	95.204(2)°
β =	92.162(3)°	66.151(2)°	98.646(2)°
γ =	90°	85.299(2)°	107.468(2)°
volume ($Å^3$)	3503.2(3)	1289.60(9)	1988.29(14)
Z	8	1	1
calcd. density (g/cm^3)	1.253	2.565	4.157
absorption coefficient (mm^{-1})	2.095	7.966	4.157
F(000)	1412	940	1456
crystal size (mm^3)	0.093×0.076×0.035	0.310×0.227×0.100	0.293×0.232×0.214
θ range for data collection (°)	3.917 to 65.599	1.834 to 33.140	1.565 to 26.998
index ranges	-22≤h≤22, -9≤k≤9, -24≤l≤25	-15≤h≤15, -18≤k≤18, -17≤l≤18	-15≤h≤15, -16≤k≤16, -16≤l≤16
reflections collected	13308	43682	47107
independent reflections	2964 [R(int) = 0.0387]	9763 [R(int) = 0.0259]	8445 [R(int) = 0.0239]

completeness to θ =	65.599, 97.6%	25.242, 99.3%	25.242, 97.6%
absorption correction	semi-empirical from equivalents	numerical	numerical
min./max. transmission	0.7528 / 0.6717	0.5181 / 0.1905	0.5578 / 0.4085
refinement method	full-matrix least-square on F^2		
data/restraints/parameter	2964 / 12 / 218	9763 / 0 / 283	8445 / 7 / 524
goodness-of-fit on F^2	1.016	1.030	1.038
final R indices [I > 2σ(I)]	R1=0.0364, wR2=0.0919	R1=0.0246, wR2=0.0611	R1=0.0571, wR2=0.1261
R indices (all data)	R1=0.0470, wR2= 0.0967	R1=0.0347, wR2=0.0635	R1=0.0659, wR2=0.1313
extinction coefficient	n/a	0.00313(12)	n/a
largest diff. peak hole (e $Å^{-3}$)	0.299 and -0.264	1.666 and -0.676	6.197 and -2.524

	**$[(C_1)G]_2[Mo_6Cl_8Cl_6]\cdot 4\ CH_2Cl_2$**<br>$Cl_{14}Mo_6$, $2(C_{14}H_{24}N_3O_3)$, $4(CH_2Cl_2)$<br>CCDC 2093024
empirical formula	$C_{32}H_{56}Cl_{22}Mo_6N_6O_6$
formula weight	1976.36
temperature (K)	150(2)
wavelength (Å)	0.710730
crystal system	triclinic
space group	*P*-1
unit cell dimensions	
a =	9.1196(10) Å
b =	10.2247(12) Å
c =	17.4472(17) Å
α =	97.888(4)°
β =	91.128(4)°
γ =	97.084(4)°
volume ($Å^3$)	1598.1(3)
Z	1
calcd. density (g/cm^3)	2.054
absorption coefficient (mm^{-1})	2.104
F(000)	964
crystal size (mm^3)	0.23×0.17×0.08
θ range for data collection (°)	2.027 to 27.52

index ranges	-11≤h≤11,<br>-13≤k≤13,<br>-22≤l≤22
reflections collected	34552
independent reflections	7305 [R(int) = 0.0585]
completeness to θ =	27.527, 99.1%
absorption correction	MULTI-SCAN
min./max. transmission	0.657 / 0.845
refinement method	
data/restraints/parameter	7305 / 3 / 330
goodness-of-fit on F^2	1.043
final R indices [I > 2σ(I)]	R1=0.0643, wR2=0.1567
R indices (all data)	R1=0.0834, wR2= 0.1725
extinction coefficient	n/a
largest diff. peak hole (e $Å^{-3}$)	2.435 and -2.503

4) Differential Scanning Calorimetry (DSC) Data

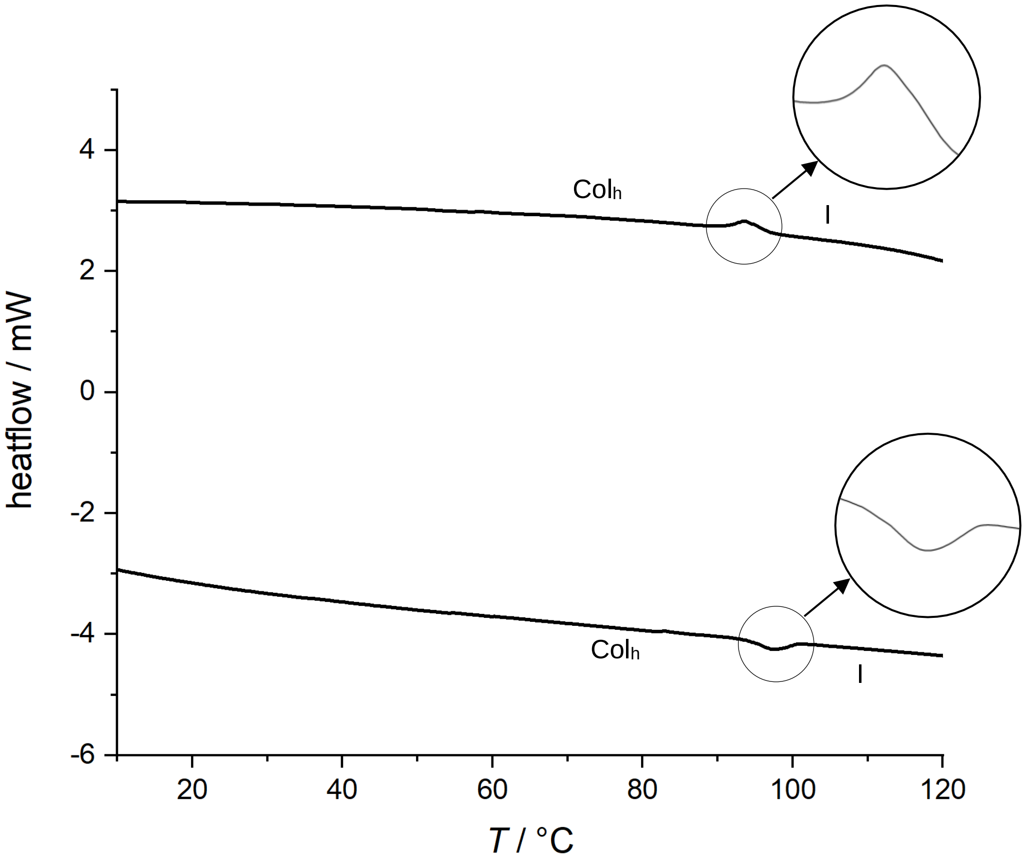

Figure S3: DSC curve of **(C10)GCl** (2nd heating/cooling cycle, 10 K/min). Inset: Expansion of the observed phase transitions.

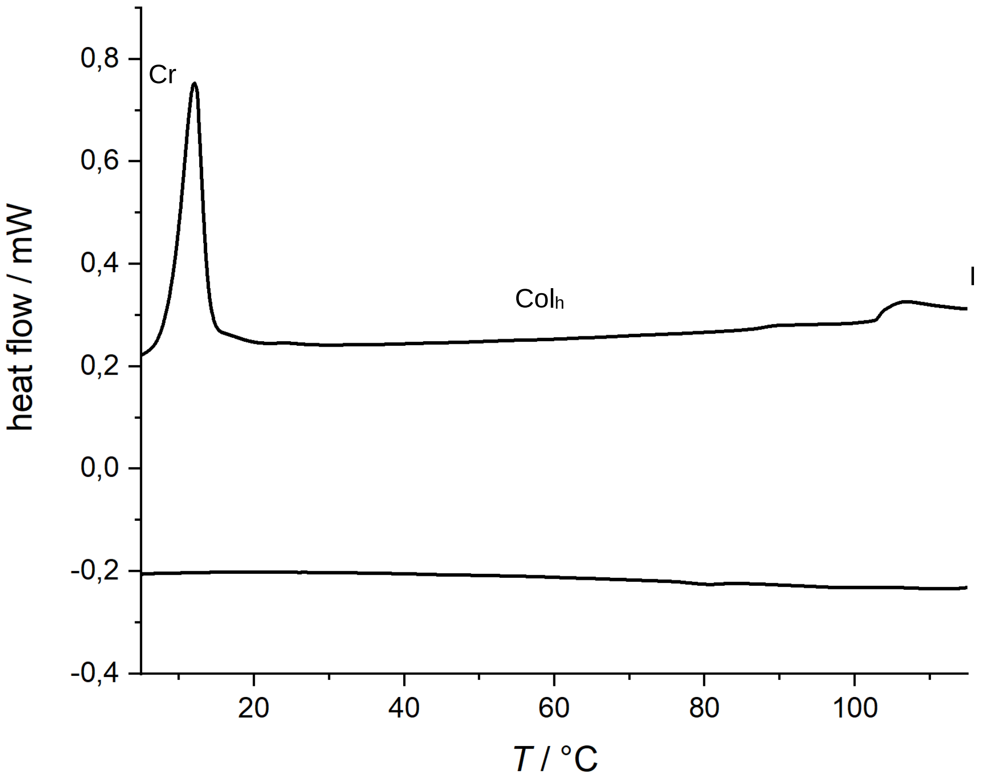

Figure S4: DSC curve of **(C_{12})GCl** (2nd heating/cooling cycle, 10 K/min).

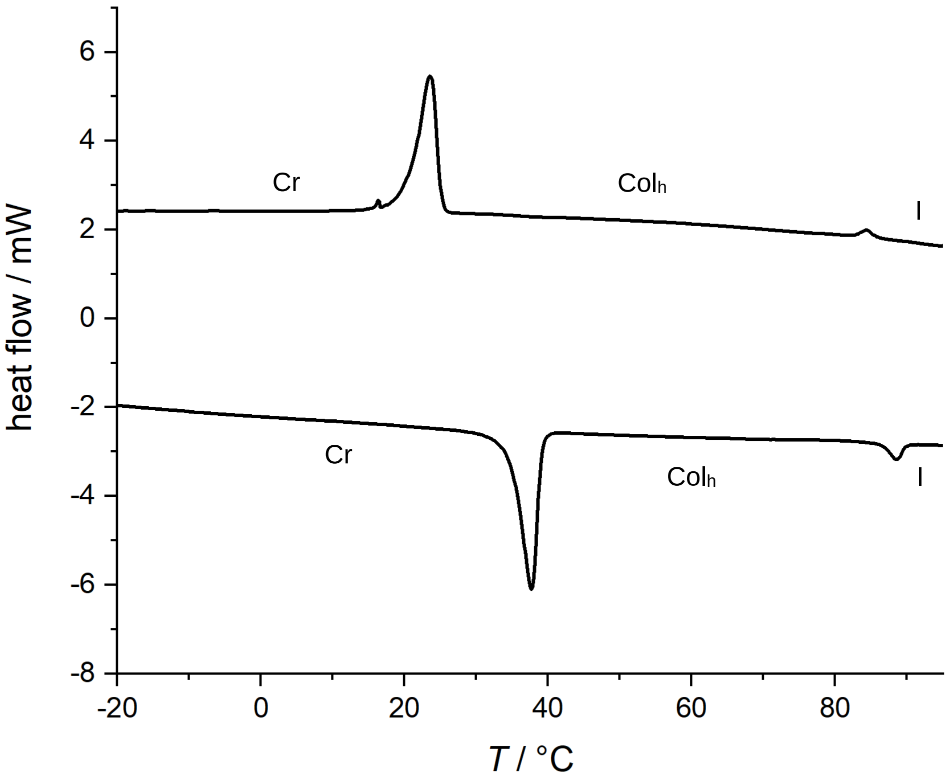

Figure S5: DSC curve of **(C_{14})GCl** (2nd heating/cooling cycle, 10 K/min).

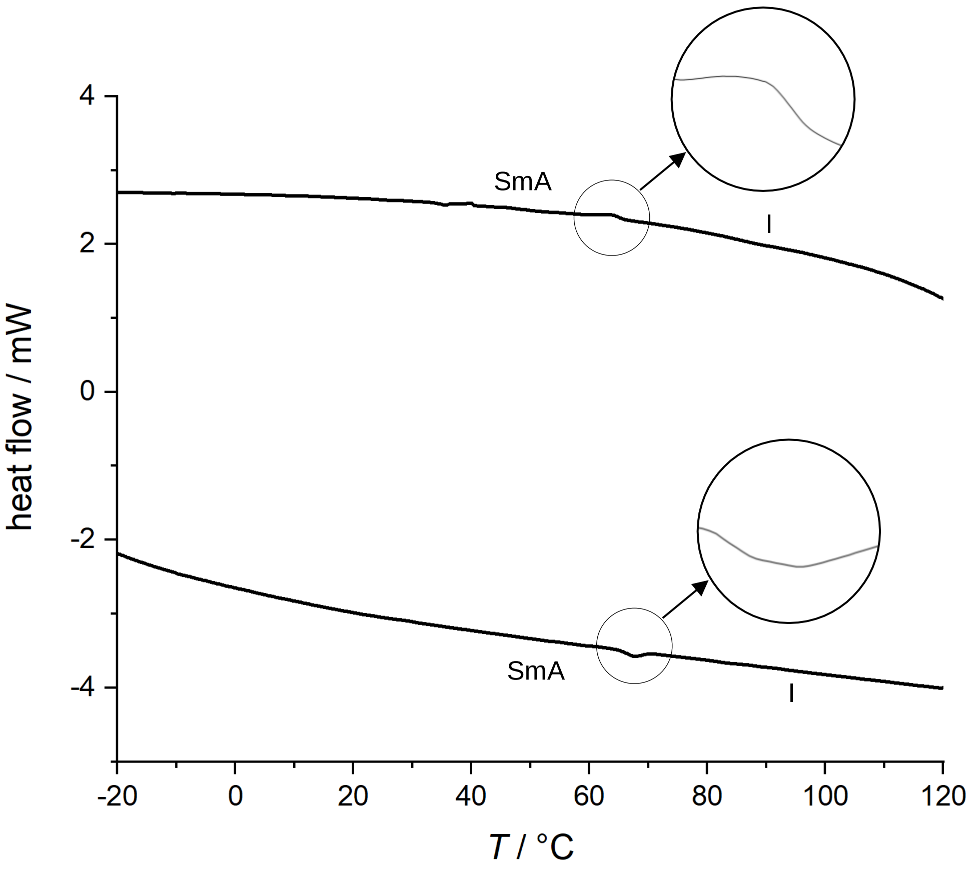

Figure S6: DSC curve of **$[(C_{10})G]_2[Mo_6Cl_8Cl_6]$** (2nd heating/cooling cycle, 10 K/min). Inset: Expansion of the observed phase transitions.

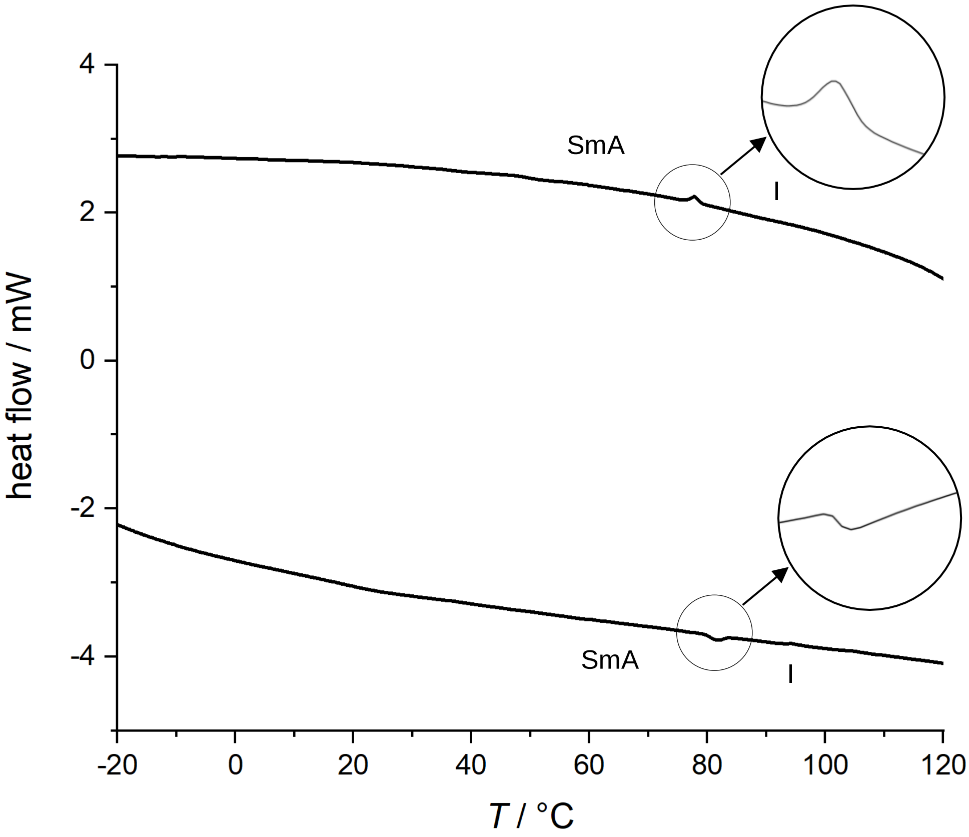

Figure S7: DSC curve of **$[(C_{12})G]_2[Mo_6Cl_8Cl_6]$** (2nd heating/cooling cycle, 10 K/min). Inset: Expansion of the observed phase transitions.

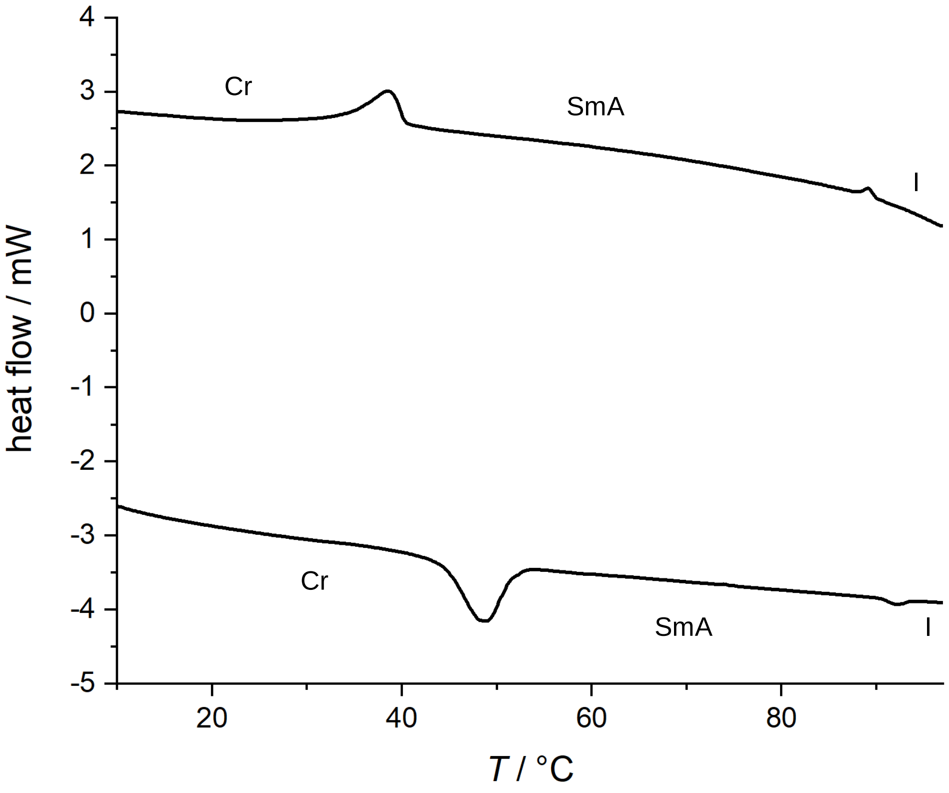

Figure S8: DSC curve of **$[(C_{14})G]_2[Mo_6Cl_8Cl_6]$** ($2^{nd}$ heating/cooling cycle, 10 K/min).

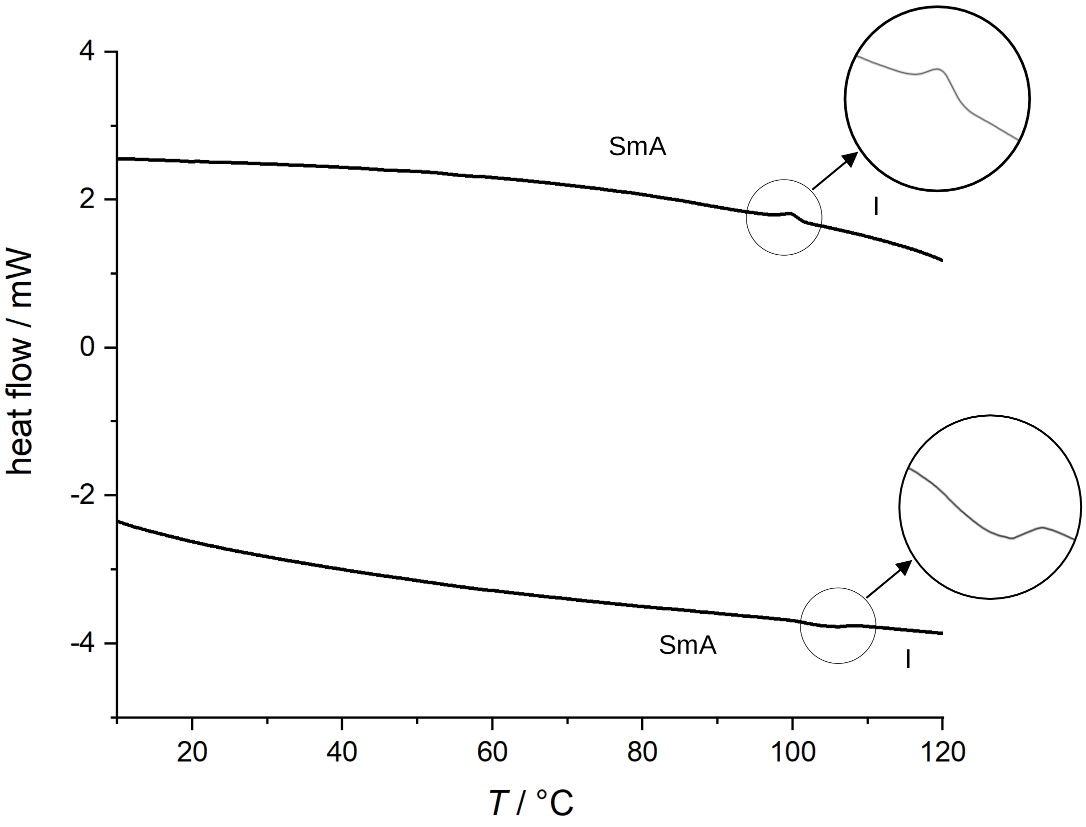

Figure S9: DSC curve of **$[(C_{10})G]_2[Mo_6Br_8Cl_6]$** ($2^{nd}$ heating/cooling cycle, 10 K/min). Inset: Expansion of the observed phase transitions.

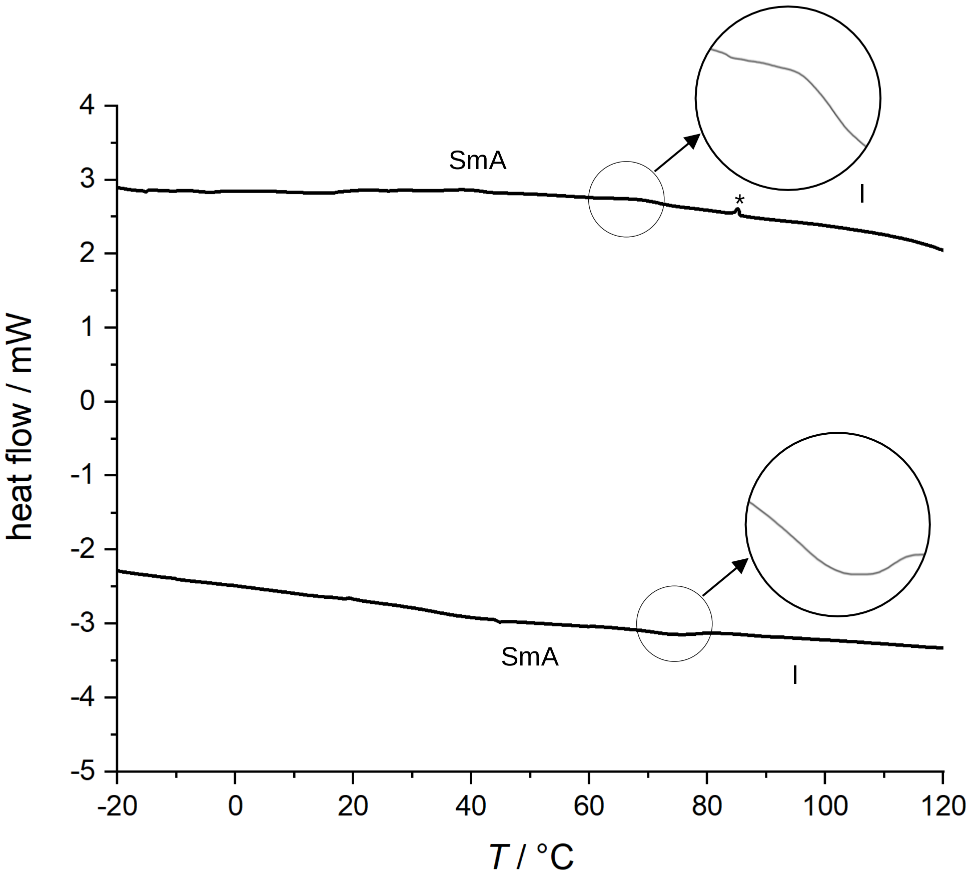

Figure S10: DSC curve of **$[(C_{12})G]_2[Mo_6Br_8Cl_6]$** ($2^{nd}$ heating/cooling cycle, 10 K/min). Inset: Expansion of the observed phase transitions. The peak marked with an asterisk is a fragment due to instabilities of the nitrogen stream.

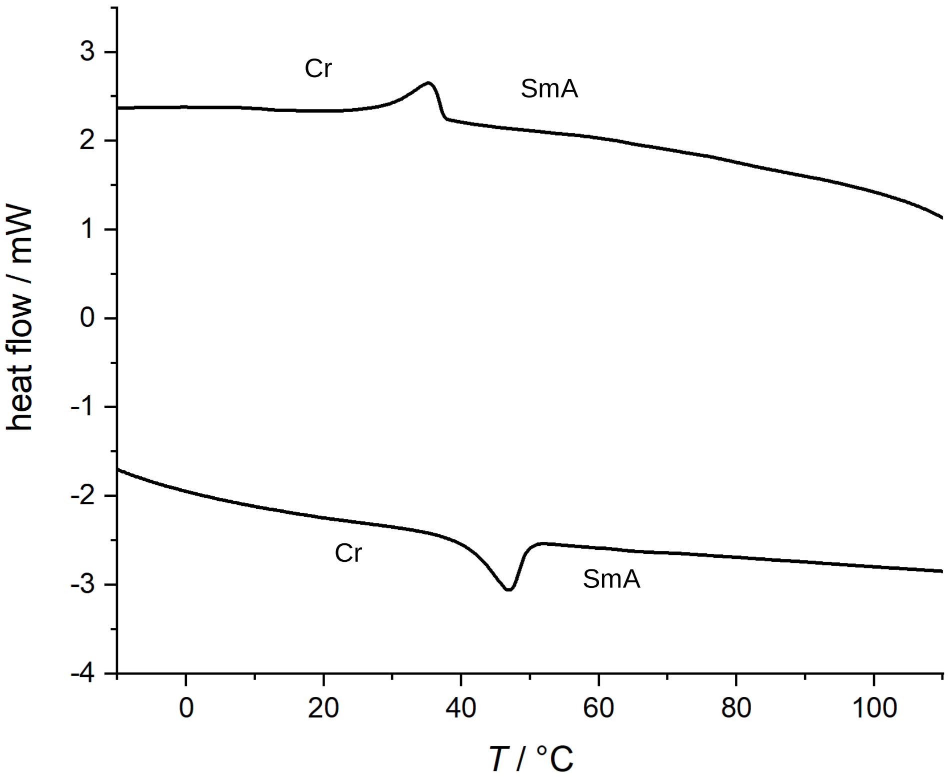

Figure S11: DSC curve of **$[(C_{14})G]_2[Mo_6Br_8Cl_6]$** ($2^{nd}$ heating/cooling cycle, 10 K/min).

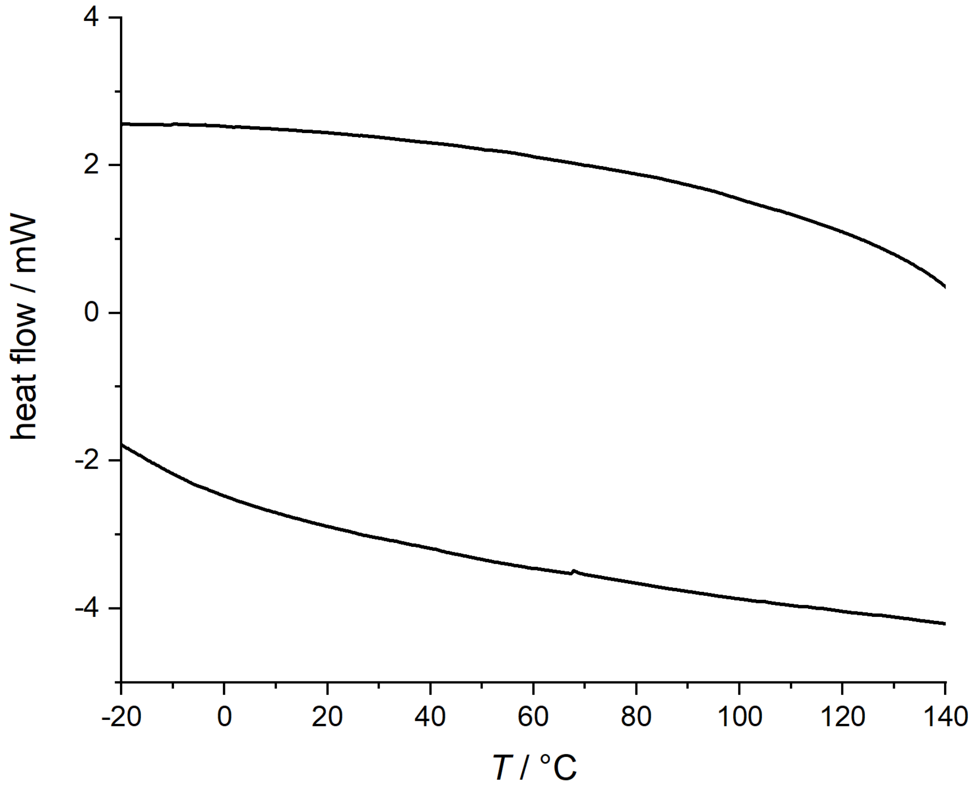

Figure S12: DSC curve of **$[(C_{10})G]_2[Mo_6I_8(C_2F_5CO_2)_6]$** (2nd heating/cooling cycle, 10 K/min).

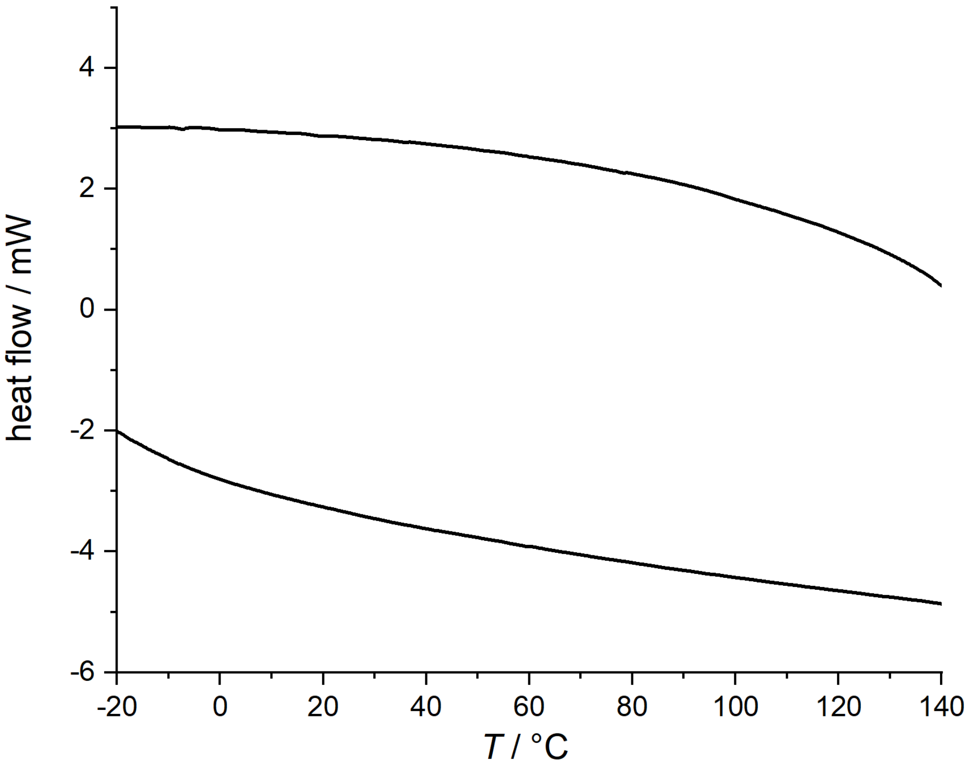

Figure S13: DSC curve of **$[(C_{12})G]_2[Mo_6I_8(C_2F_5CO_2)_6]$** ($2^{nd}$ heating/cooling cycle, 10 K/min).

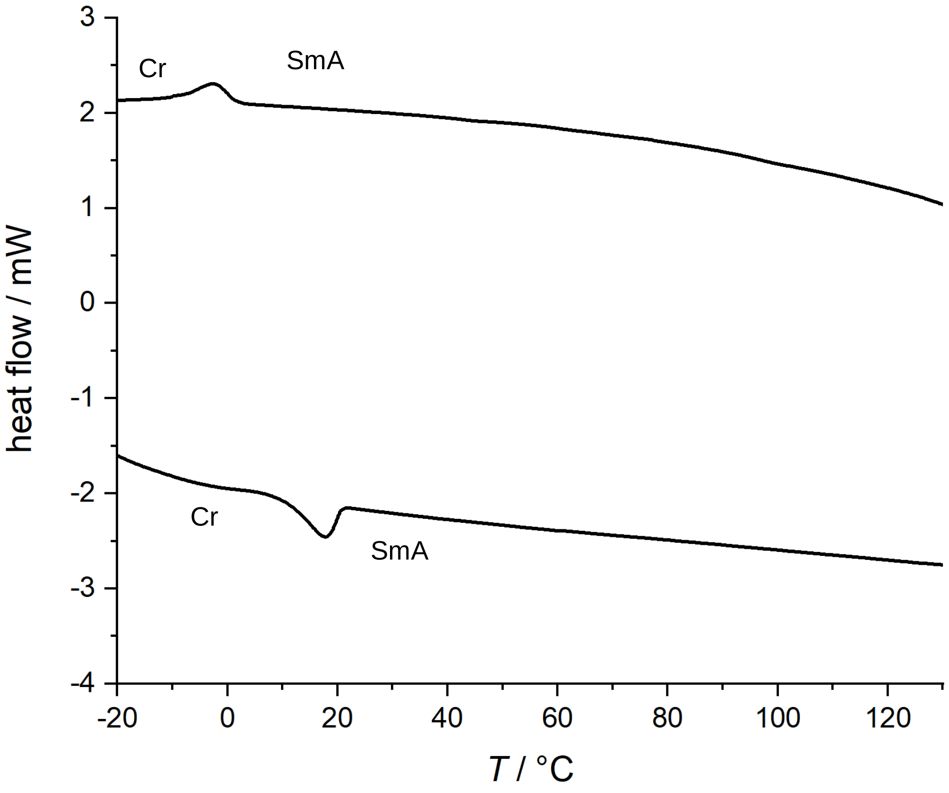

Figure S14: DSC curve of **$[(C_{14})G]_2[Mo_6I_8(C_2F_5CO_2)_6]$** (2nd heating/cooling cycle, 10 K/min).

5) Polarizing Optical Microscopy (POM) data

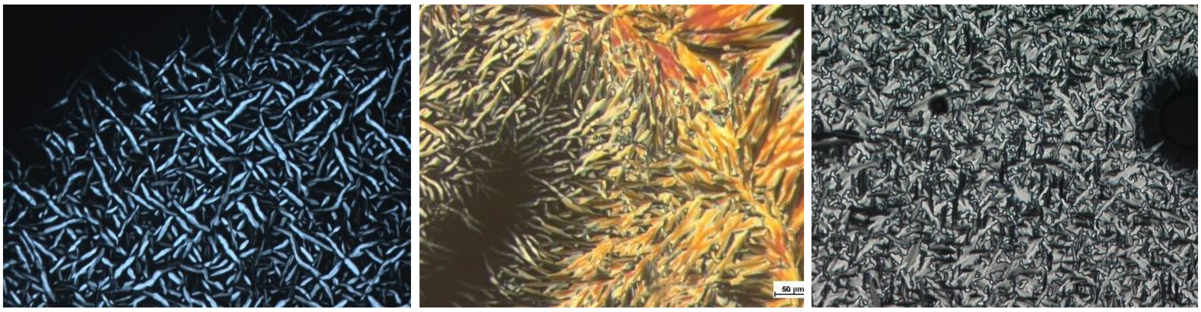

Figure S15: POM images of **$(C_{10})GCl$** (left, 85 °C, 200× magnification), **$(C_{12})GCl$** (middle, 116 °C, 100× magnification) and **$(C_{14})GCl$** (right, 86 °C, 200× magnification).

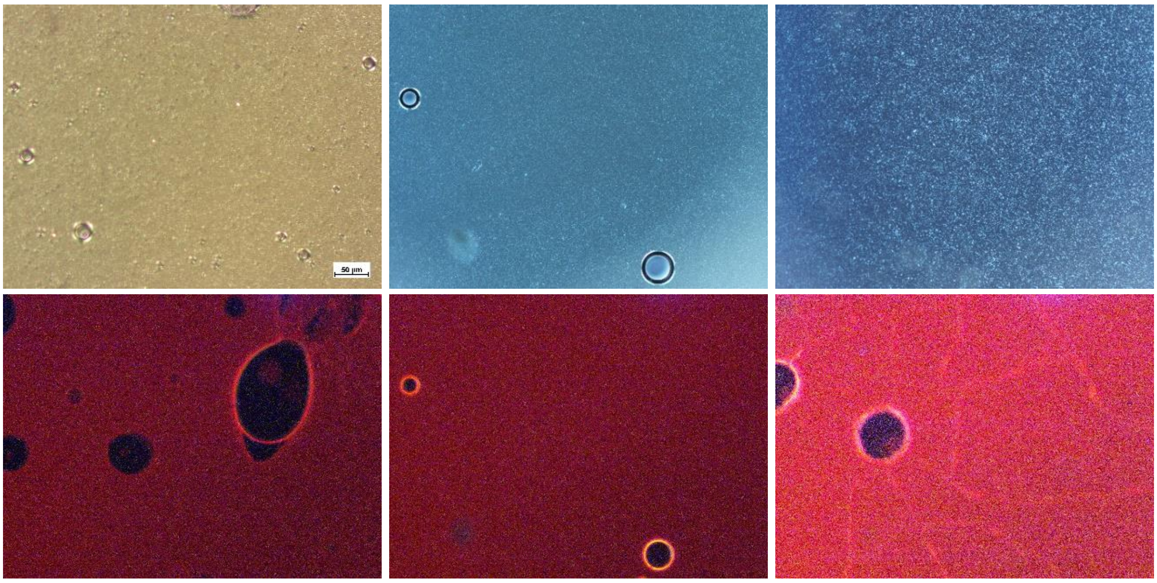

Figure S16: POM images of **$[(C_{10})G]_2[Mo_6Cl_8Cl_6]$** (left, 53 °C, 10× magnification), **$[(C_{12})G]_2[Mo_6Cl_8Cl_6]$** (middle, 80 °C, 100× magnification) and **$[(C_{14})G]_2[Mo_6Cl_8Cl_6]$** (right, 80 °C, 100× magnification) under white light (top row) und under UV irradiation (bottom row).

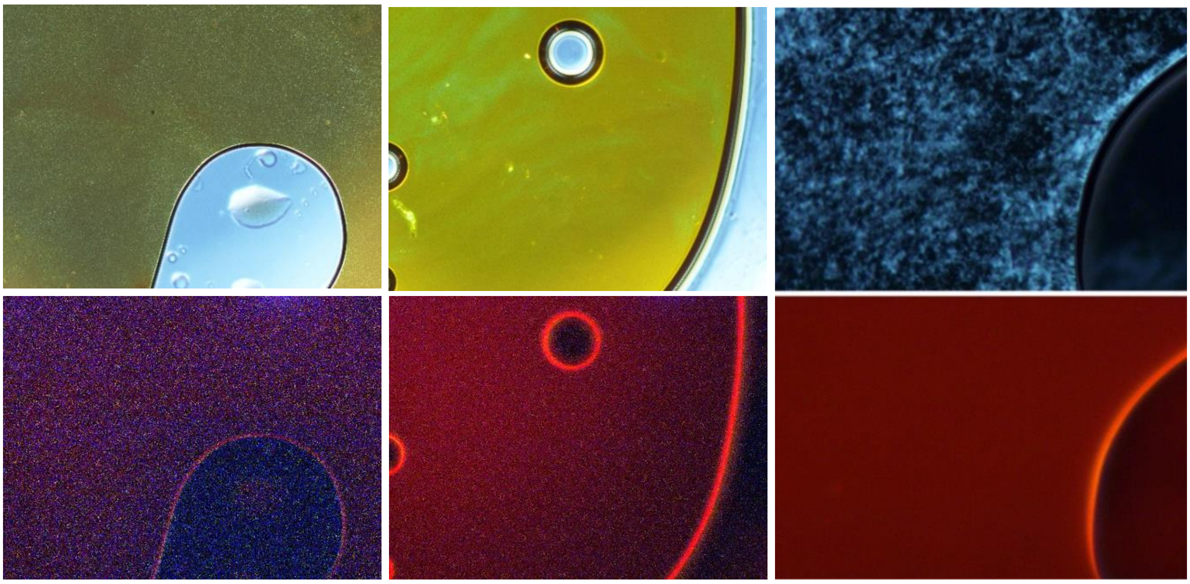

Figure S17: POM images of $\mathbf{[(C_{10})G]_2[Mo_6Br_8Cl_6]}$ (left, 90 °C, 100× magnification), $\mathbf{[(C_{12})G]_2[Mo_6Br_8Cl_6]}$ (middle, 80 °C, 100× magnification) and $\mathbf{[(C_{14})G]_2[Mo_6Br_8Cl_6]}$ (right, 66°C, 100× magnification) under white light (top row) und under UV irradiation (bottom row).

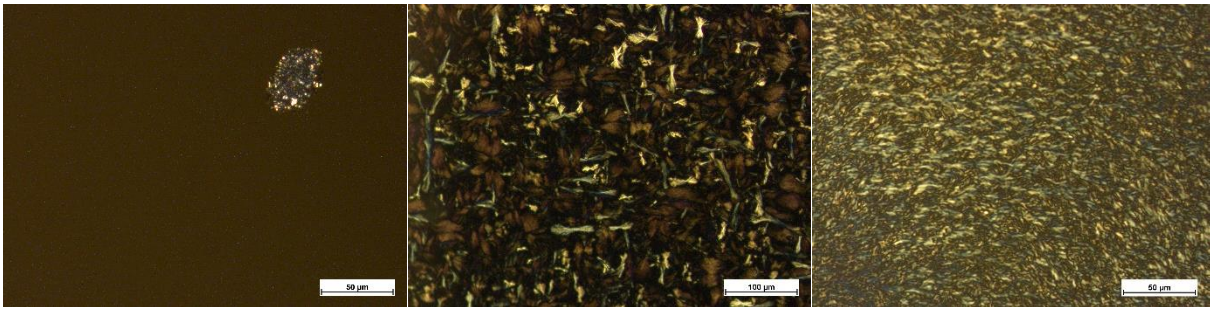

Figure S18: POM images of $\mathbf{[(C_{10})G]_2[Mo_6I_8(C_2F_5CO_2)_6]}$ (left, 48 °C, 200× magnification), $\mathbf{[(C_{12})G]_2[Mo_6I_8(C_2F_5CO_2)_6]}$ (middle, 58 °C, 100× magnification) and $\mathbf{[(C_{14})G]_2[Mo_6I_8(C_2F_5CO_2)_6]}$ (right, 55 °C, 200× magnification) under white light.

6) X-Ray Diffraction (XRD) Data

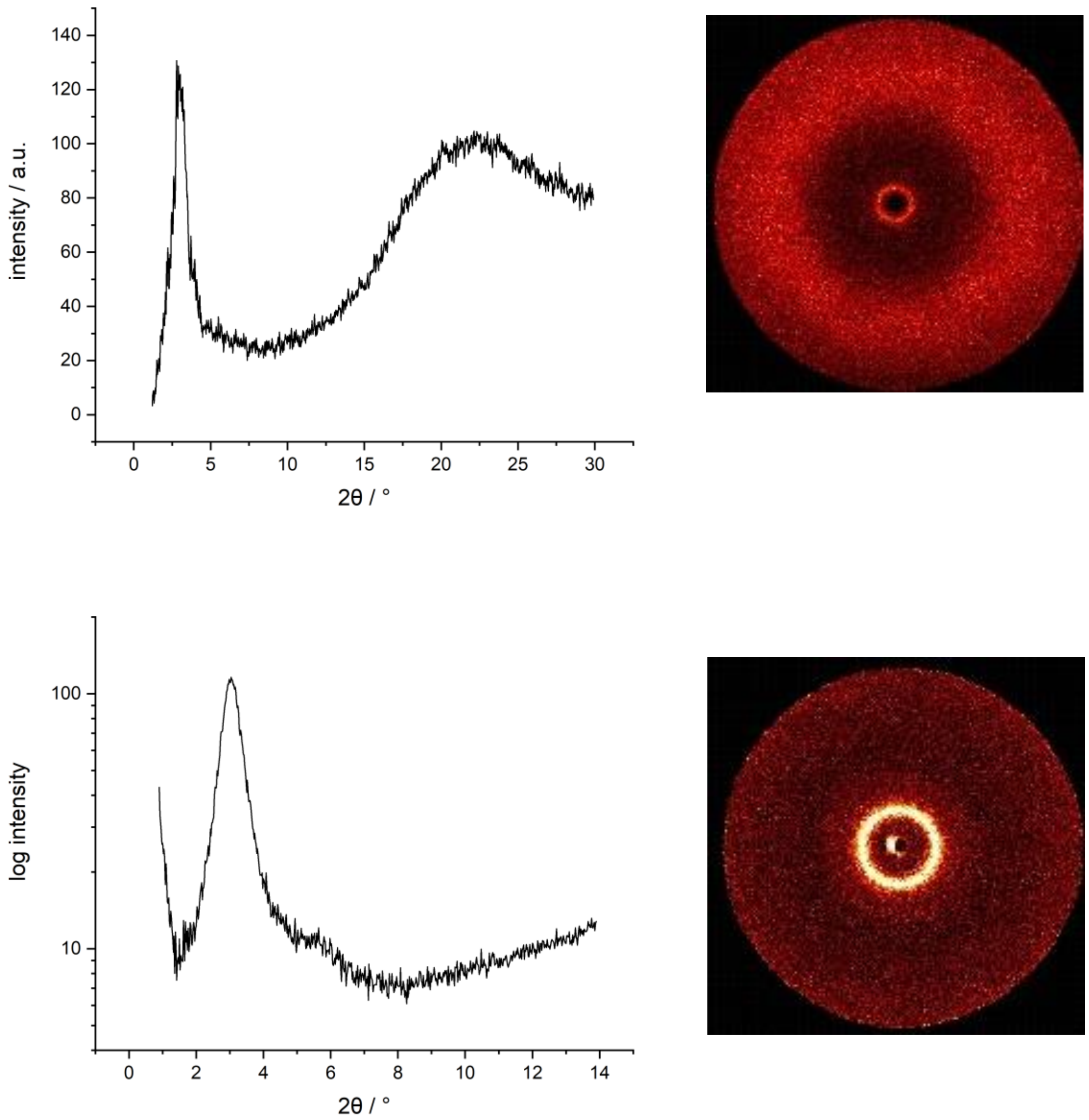

Figure S19: Wide angle (top) and small angle (bottom) X-ray diffractogram of **(C_{10})GCl** at 80 °C and 2D diffraction patterns.

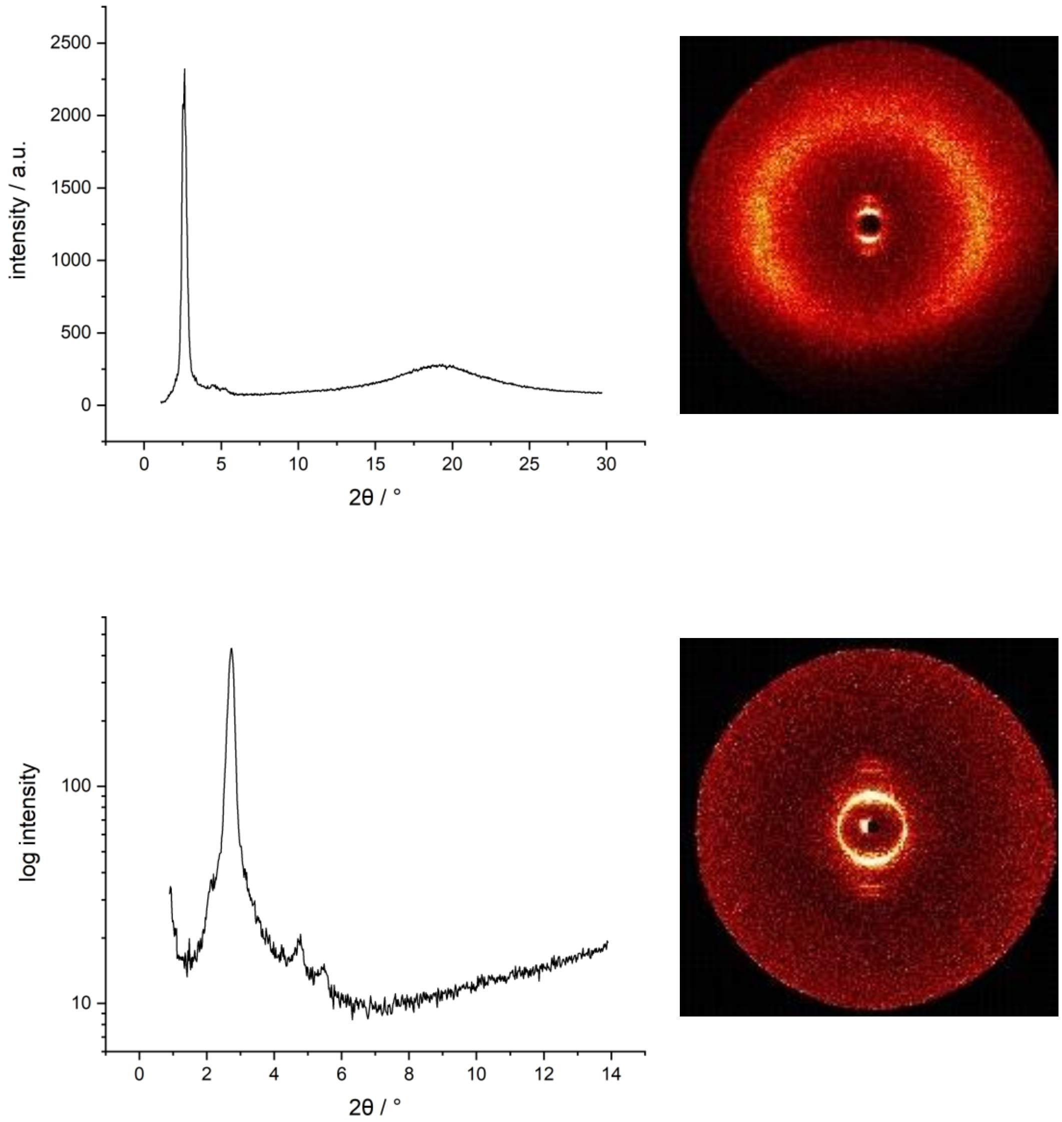

Figure S20: Wide angle (top) and small angle (bottom) X-ray diffractogram of **(C_{12})GCl** at 85 °C and 2D diffraction patterns.

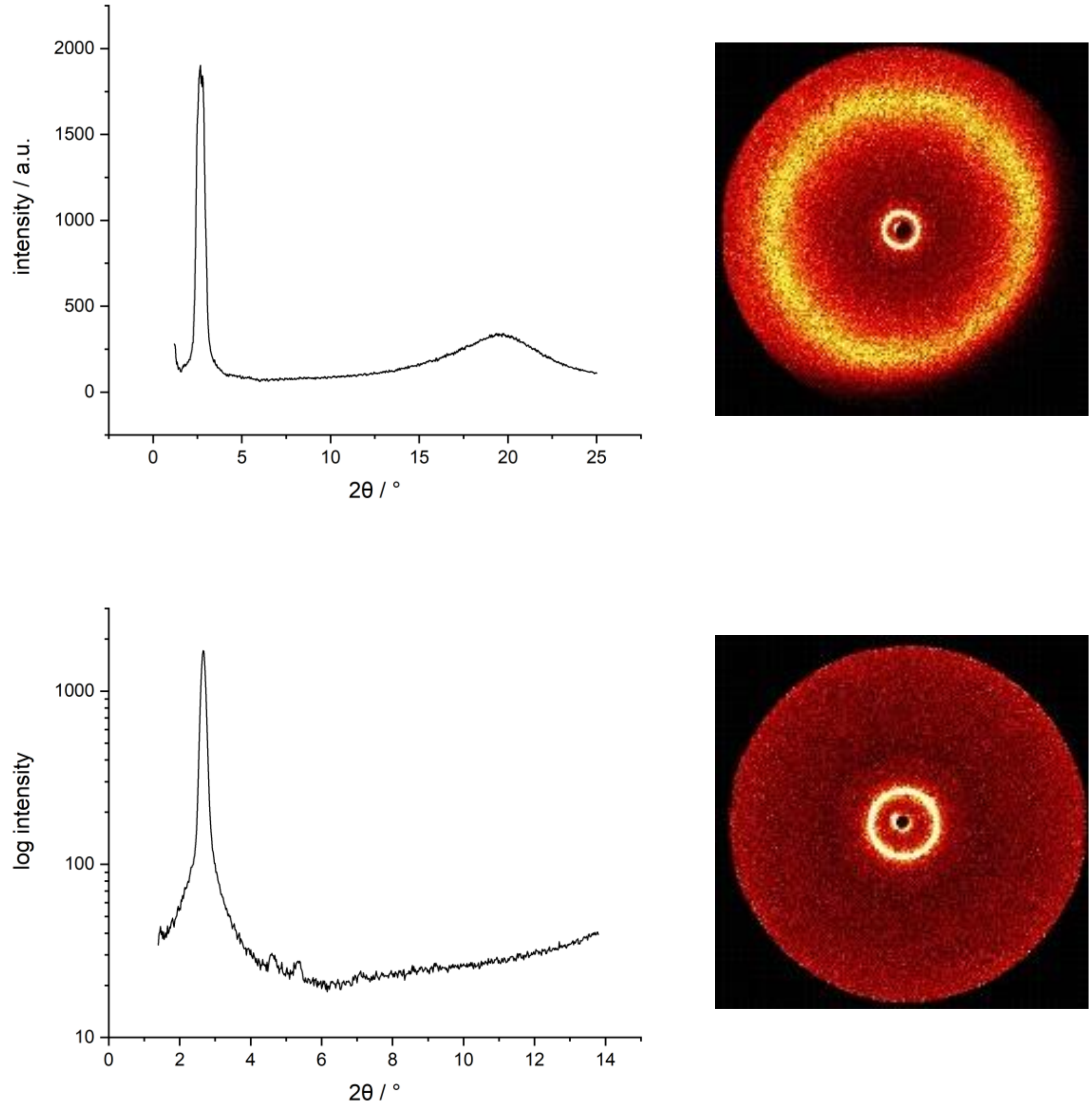

Figure S21: Wide angle (top) and small angle (bottom) X-ray diffractogram of **(C_{14})GCl** at 72 °C and 2D diffraction patterns.

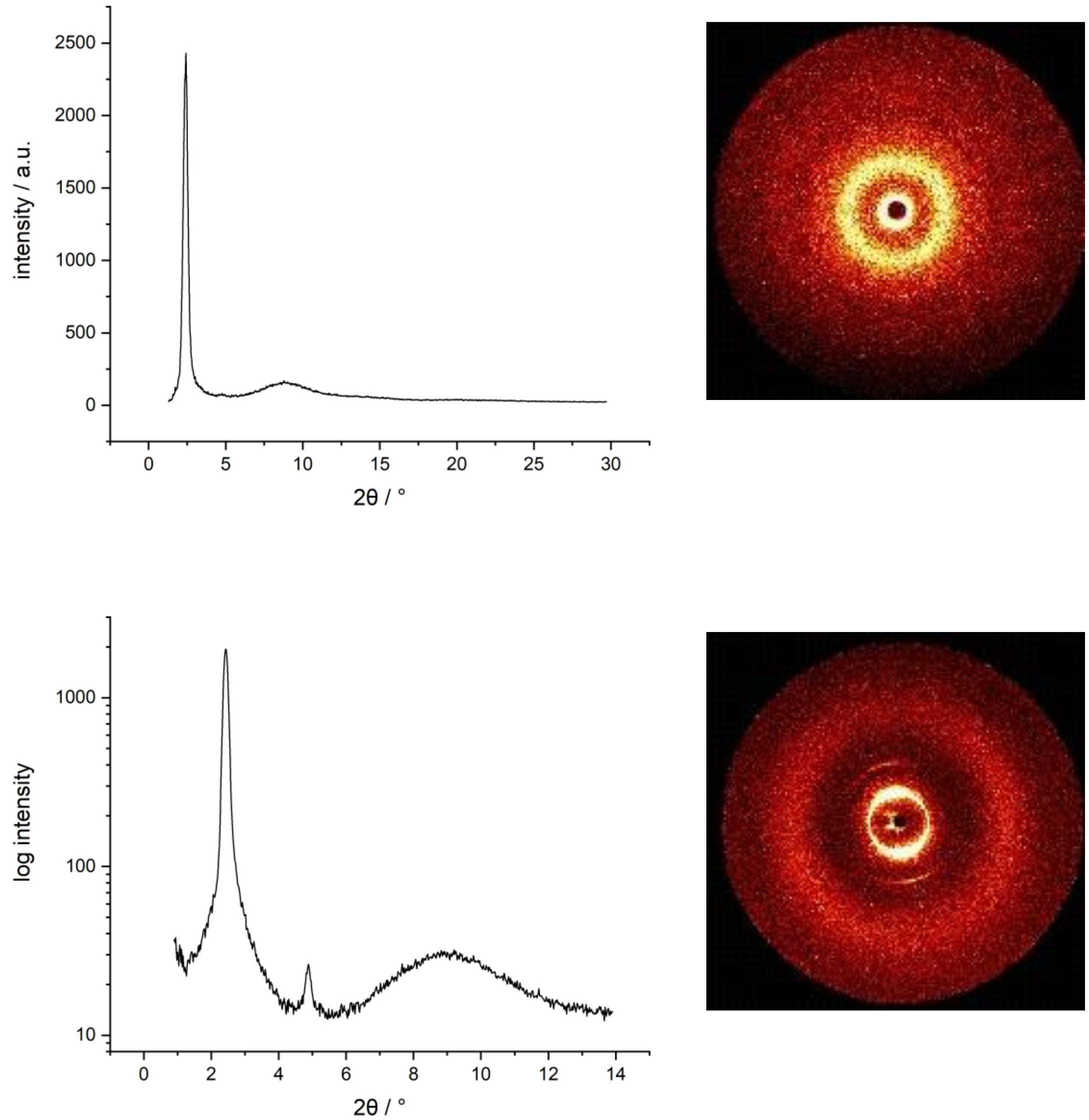

Figure S22: Wide angle (top) and small angle (bottom) X-ray diffractogram of **$[(C_{12})G]_2[Mo_6Cl_8Cl_6]$** at 60 °C and 2D diffraction patterns.

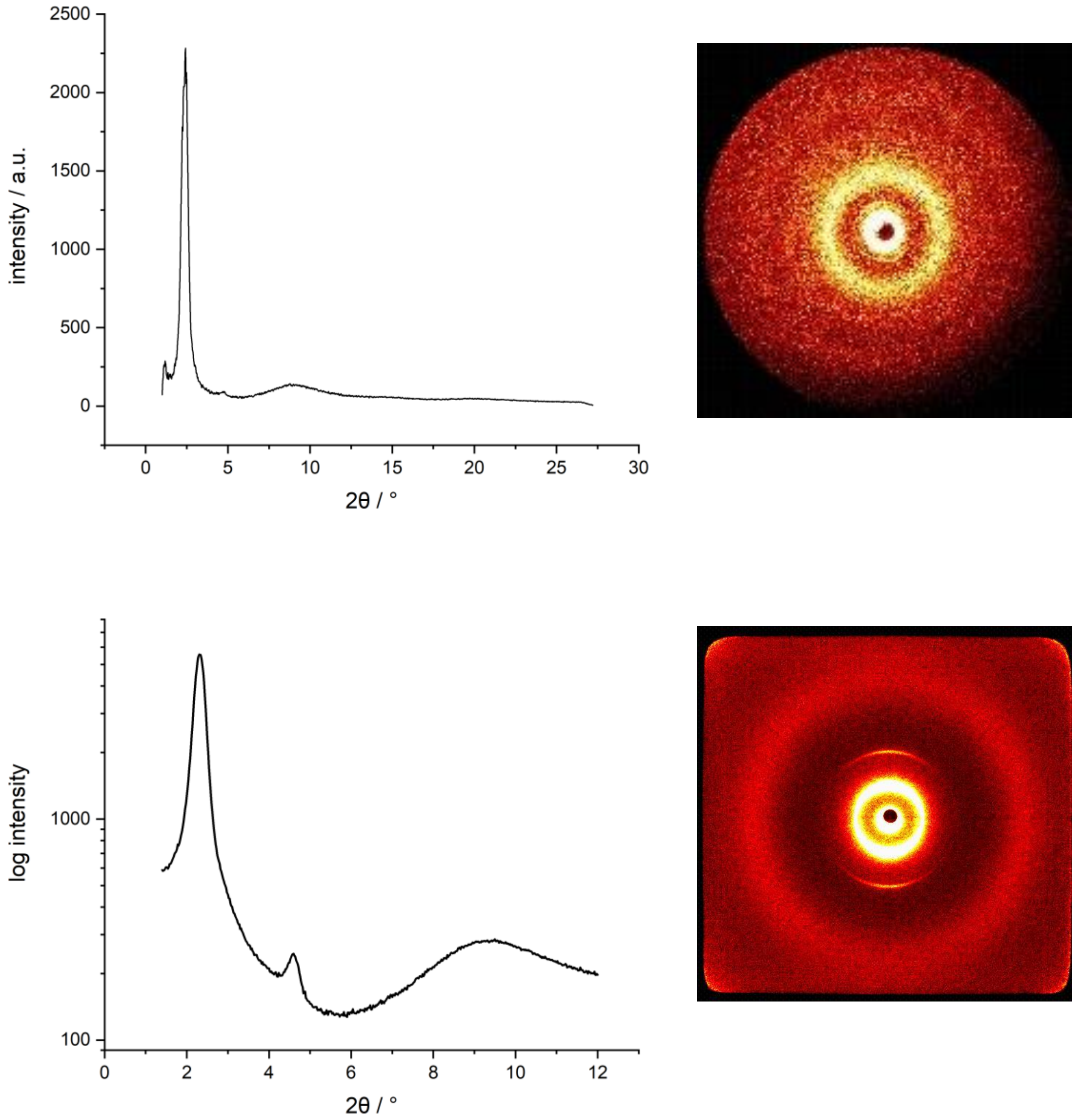

Figure S23: Wide angle (top) and small angle (bottom) X-ray diffractogram of **$[(C_{14})G]_2[Mo_6Cl_8Cl_6]$** at 50 °C and 2D diffraction patterns.

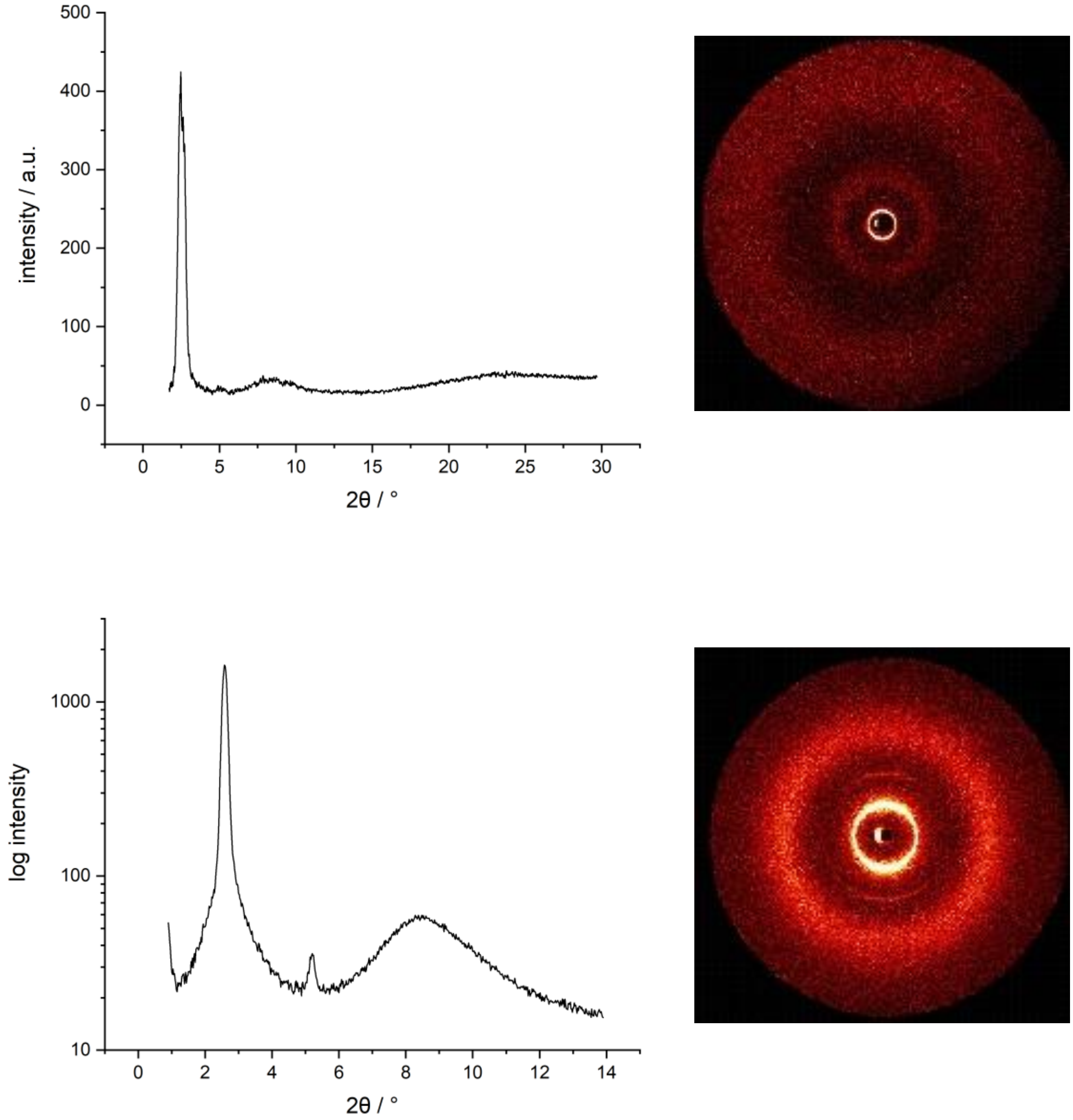

Figure S24: Wide angle (top) and small angle (bottom) X-ray diffractogram of **$[(C_{10})G]_2[Mo_6Br_8Cl_6]$** at 76 °C and 2D diffraction pattern.

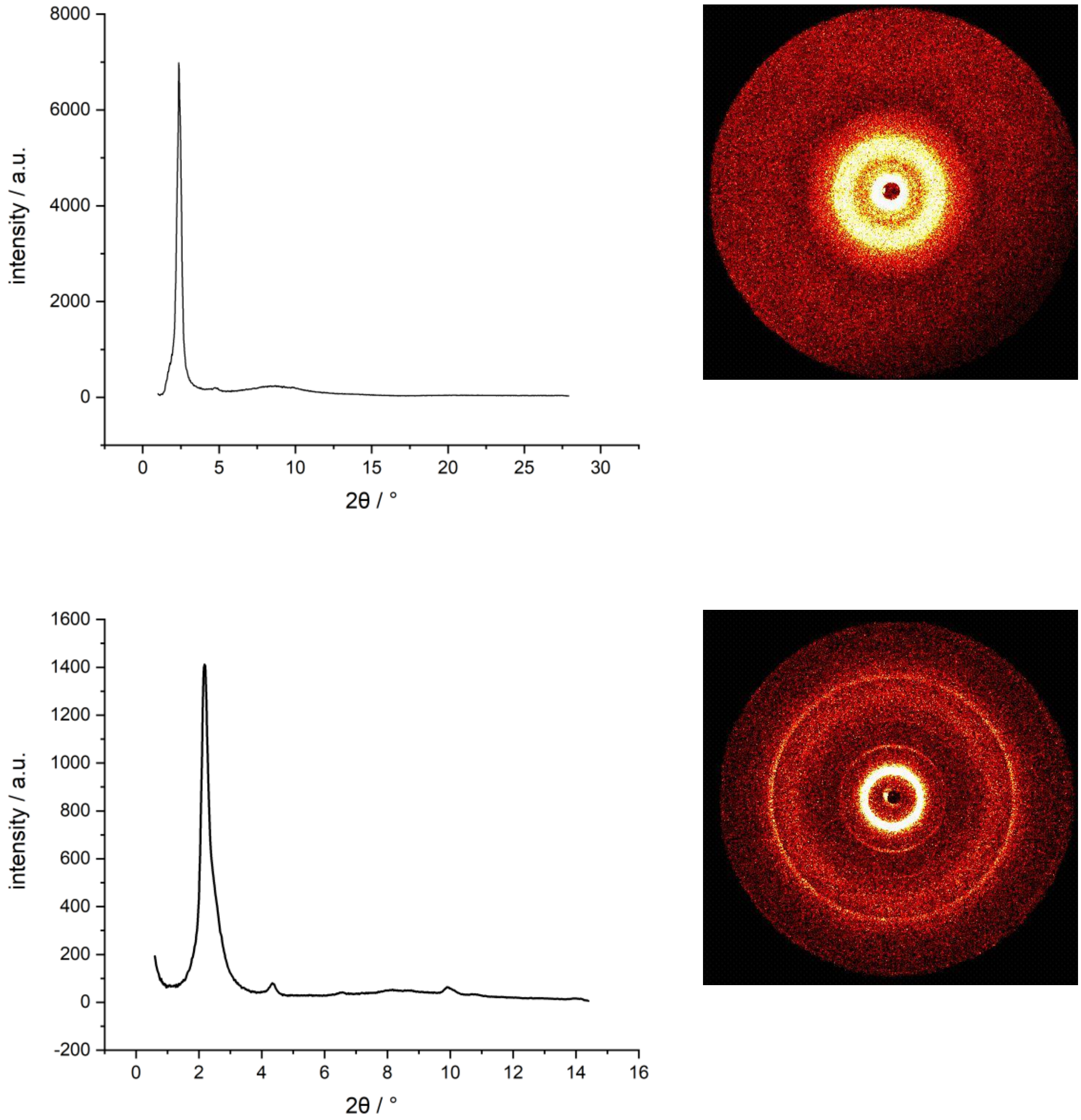

Figure S25: Wide angle (top) and small angle (bottom) X-ray diffractogram of **$[(C_{12})G]_2[Mo_6Br_8Cl_6]$** at 76 °C and 2D diffraction patterns.

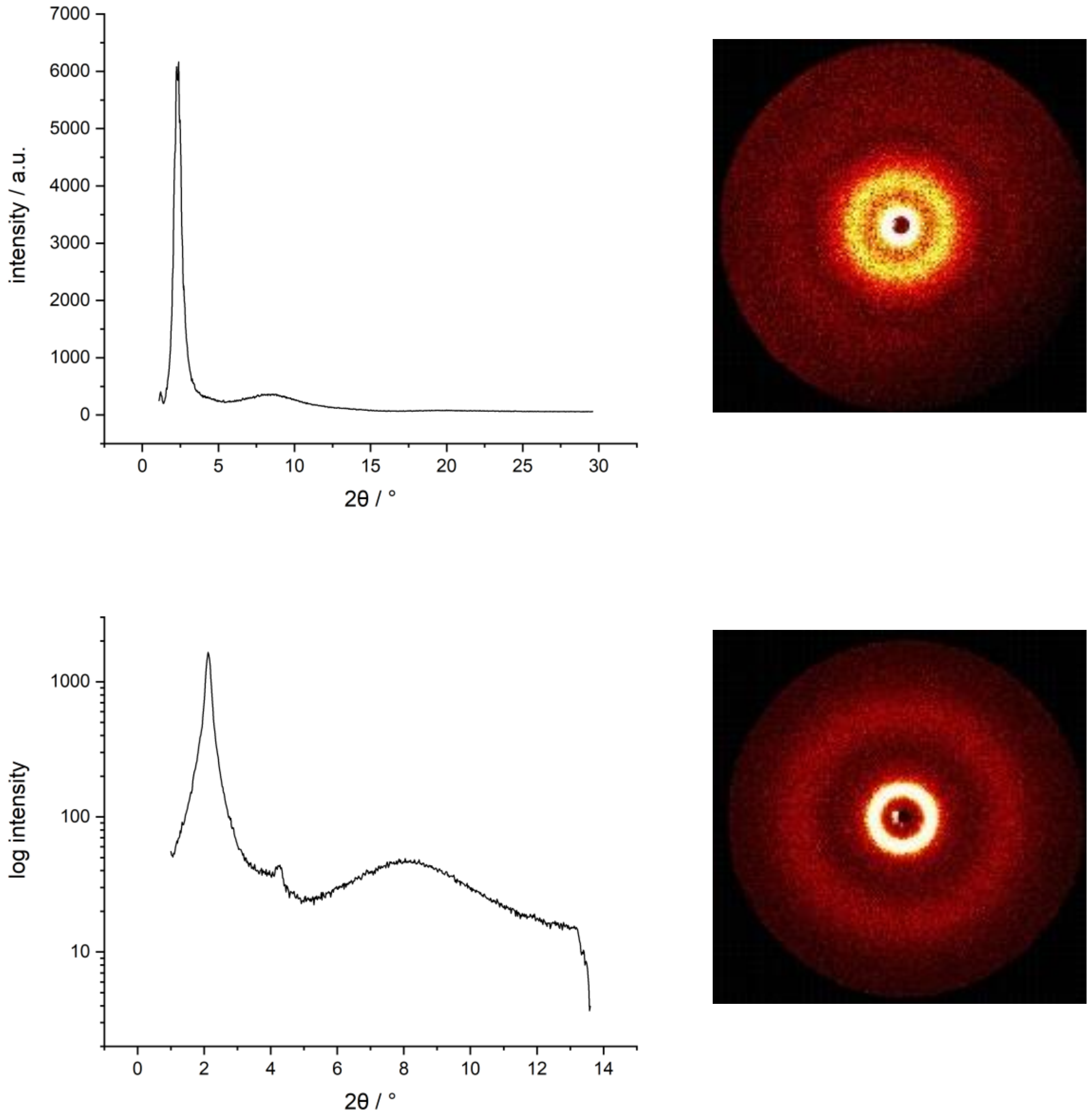

Figure S26: Wide angle (top) and small angle (bottom) X-ray diffractogram of **$[(C_{14})G]_2[Mo_6Br_8Cl_6]$** at 80 °C and 2D diffraction patterns.

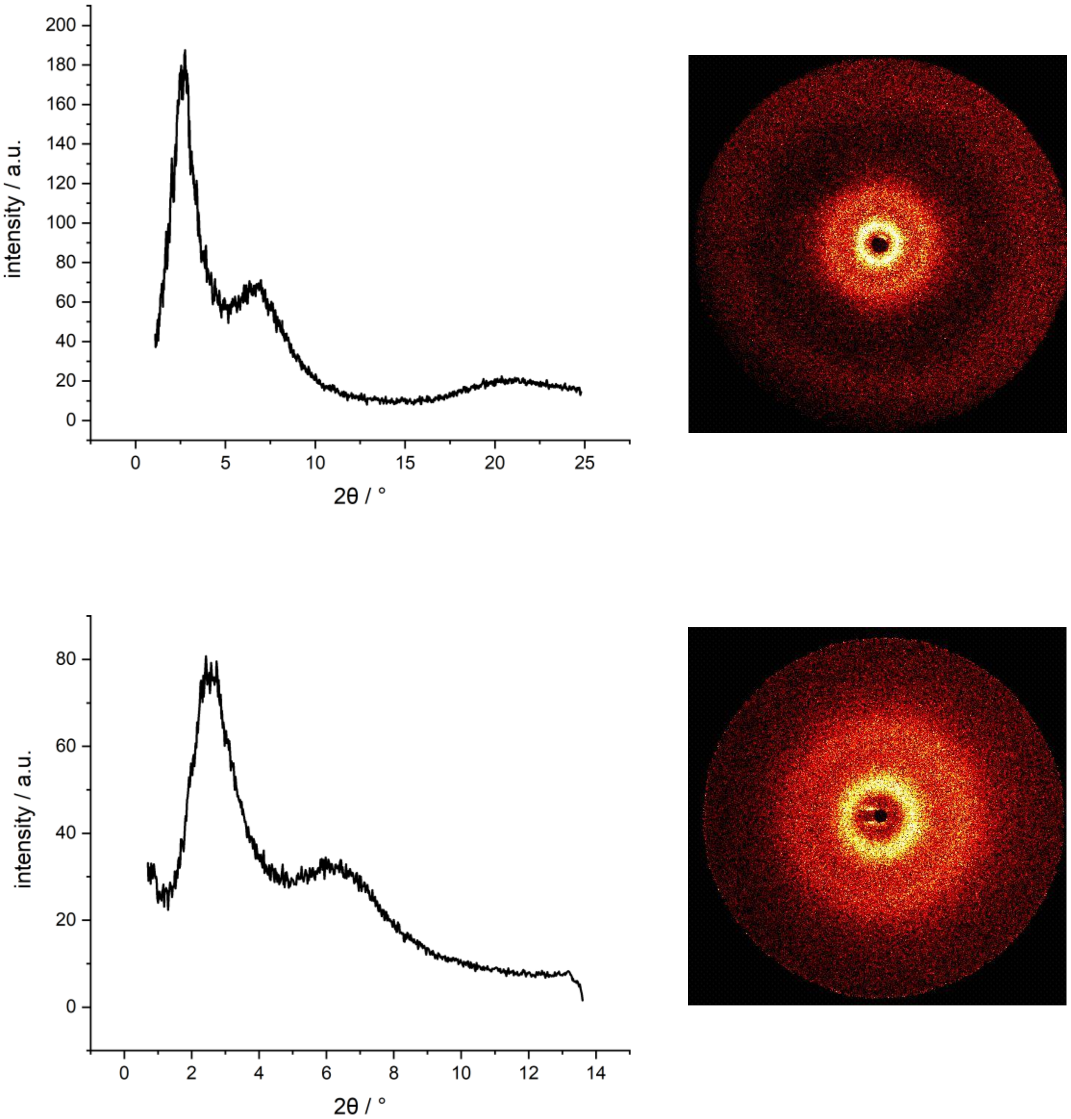

Figure S27: Wide angle (top) and small angle (bottom) X-ray diffractogram of **$[(C_{14})G]_2[Mo_6I_8(C_2F_5CO_2)_6]$** at 68 °C and 2D diffraction patterns.

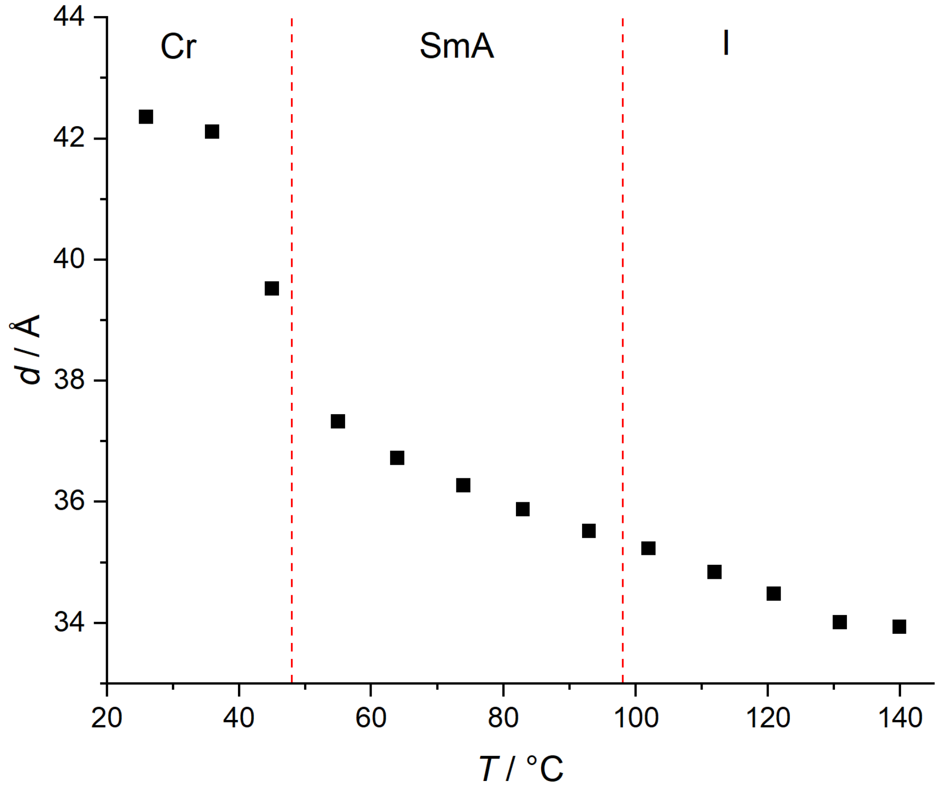

Figure S28: Temperature-dependent intermolecular distance *d* of **$[(C_{12})G]_2[Mo_6Br_8Cl_6]$** determined from the (001) reflex in the diffraction pattern. In the SmA mesophase, *d* represents the layer spacing. Red dashed lines: Phase transitions according to DSC data.

7) Photoluminescence Data

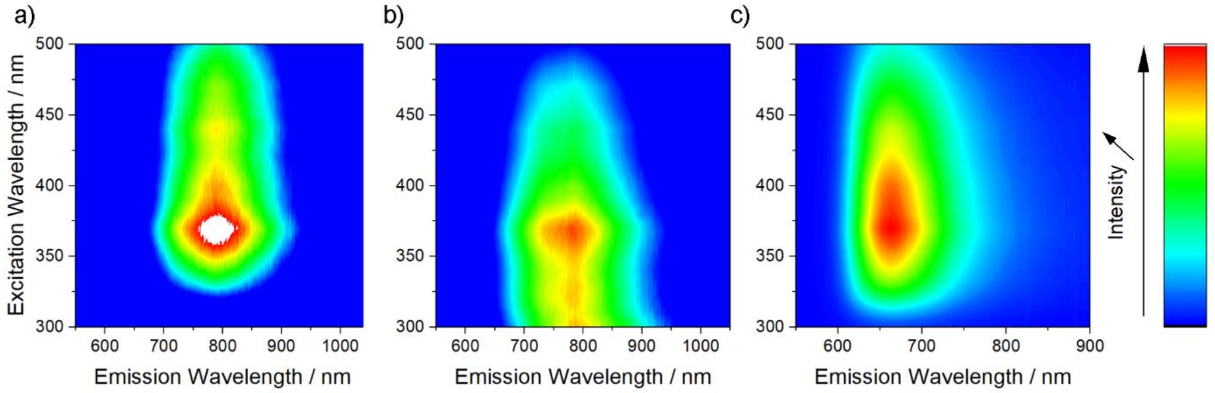

Figure S29: Emission *vs* excitation map for a) $K_2[Mo_6Br_8Cl_6]$, b) $Cs_2[Mo_6Cl_8Cl_6]$ and c) $Cs_2[Mo_6I_8(OCOC_2F_5)_6]$.

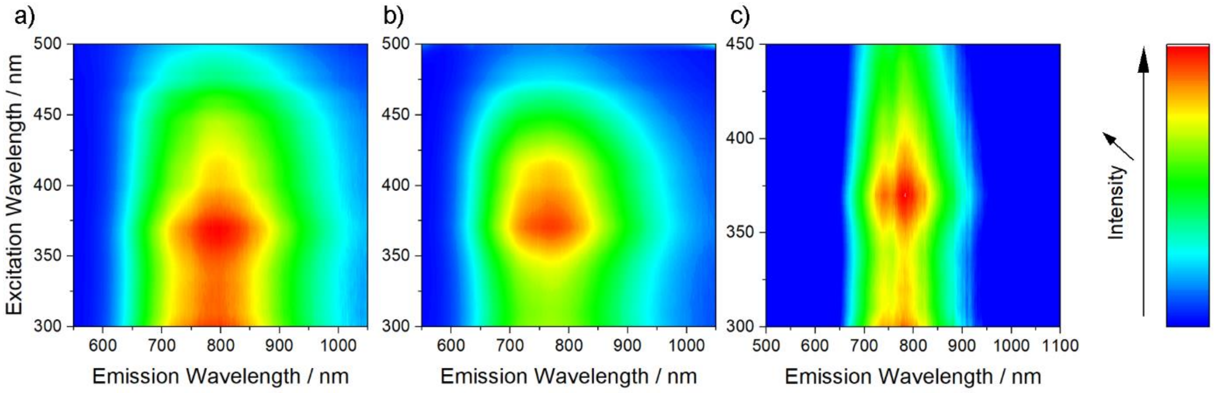

Figure S30: Emission *vs* excitation map for $[Mo_6Br_8Cl_6]^{2-}$ associated with a) **$[(C_{10})G]^+$**, b) **$[(C_{12})G]^+$**, c) **$[(C_{14})G]^+$**.

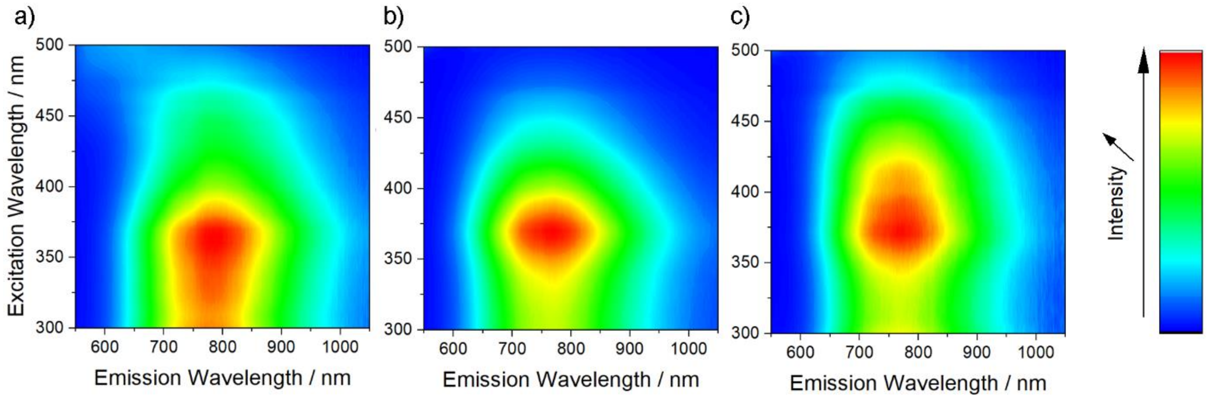

Figure S31: Emission *vs* excitation map for $[Mo_6Cl_8Cl_6]^{2-}$ associated with a) **$[(C_{10})G]^+$**, b) **$[(C_{12})G]^+$**, c) **$[(C_{14})G]^+$**.

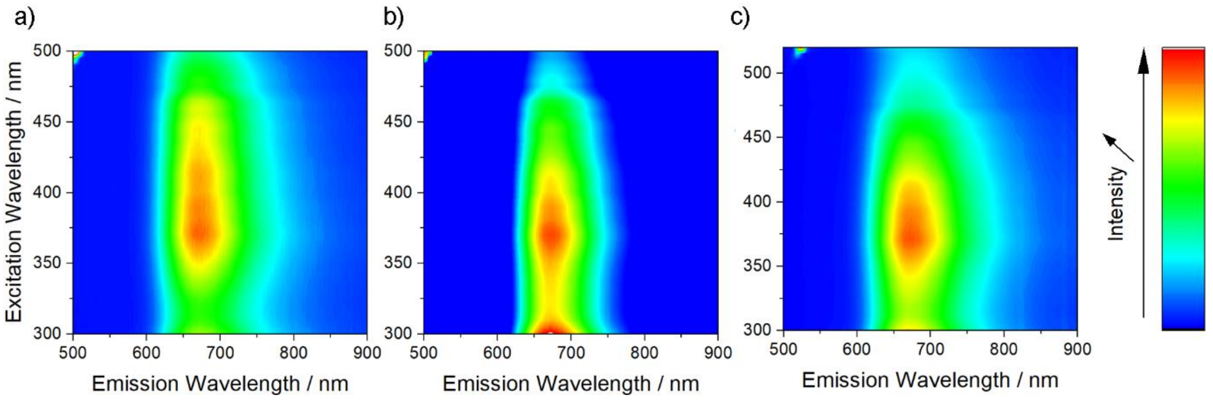

Figure S32: Emission *vs* excitation map for $[Mo_6I_8(OCOC_2F_5)_6]^{2-}$ associated with a) **$[(C_{10})G]^+$**, b) **$[(C_{12})G]^+$**, c) **$[(C_{14})G]^+$**.

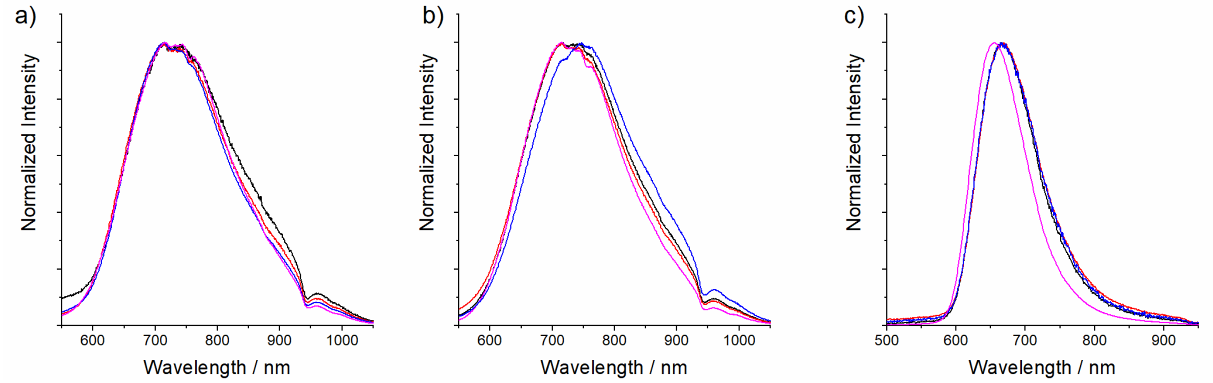

Figure S33: Emission spectra (λ_{exc} = 375 nm) of a) $[Mo_6Br_8Cl_6]^{2-}$, b) $[Mo_6Cl_8Cl_6]^{2-}$ and c) $[Mo_6I_8(OCOC_2F_5)_6]^{2-}$ with **$[(C_{10})G]^+$** (in black), **$[(C_{12})G]^+$** (in red), **$[(C_{14})G]^+$** (in blue) and as an alkali salt (in magenta).

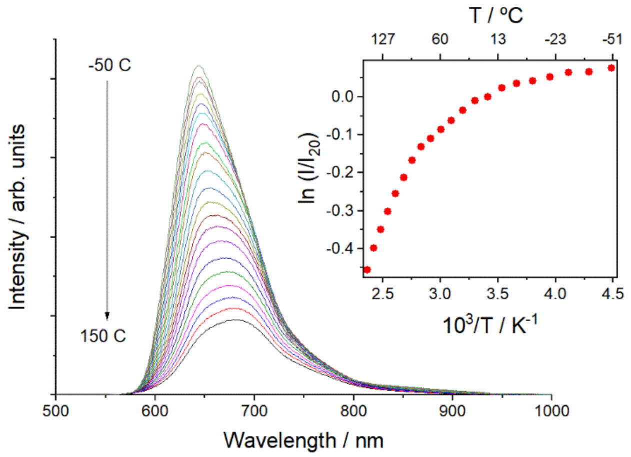

Figure S34: Evolution of the emission signal of **$Cs_2[Mo_6I_8(OCOC_2F_5)_6]$** in powder with temperature.

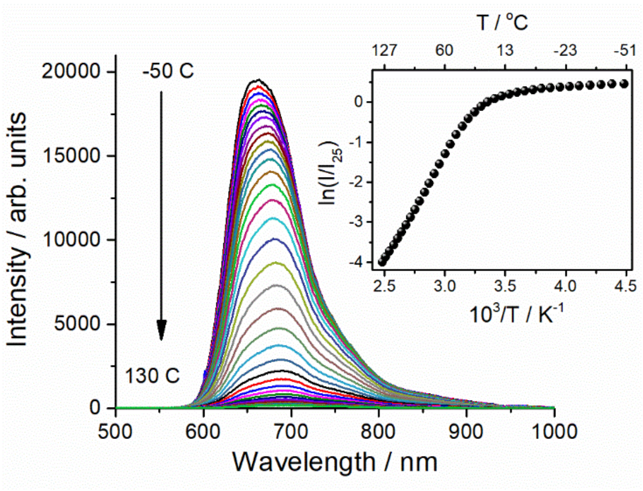

Figure S35: Evolution of the emission signal of **$[(C_{10})G]_2[Mo_6I_8(OCOC_2F_5)_6]$** with temperature.

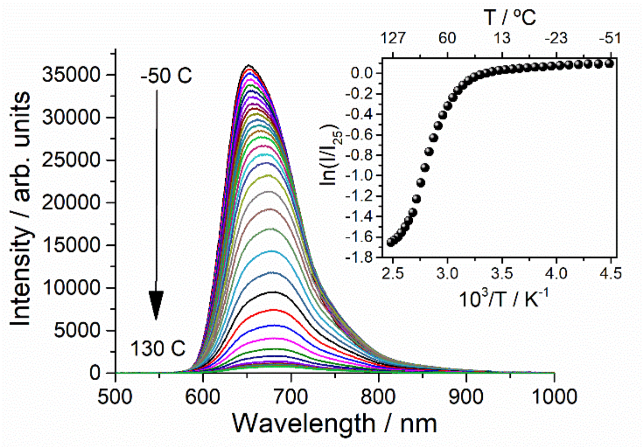

Figure S36: Evolution of the emission signal of **$[(C_{12})G]_2[Mo_6I_8(OCOC_2F_5)_6]$** with temperature.

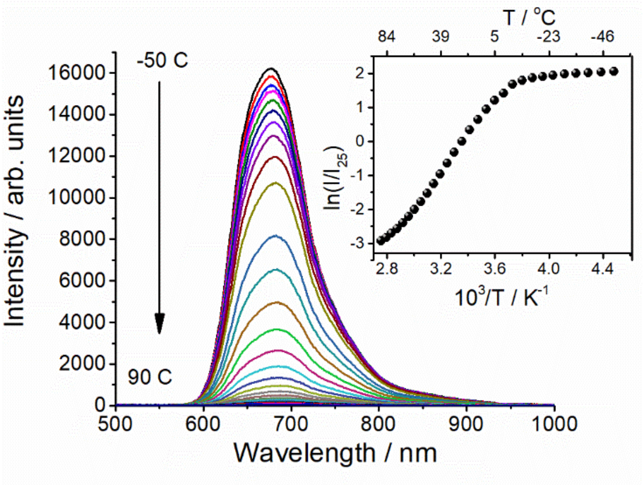

Figure S37: Evolution of the emission signal of **$[(C_{14})G]_2[Mo_6I_8(OCOC_2F_5)_6]$** with temperature.

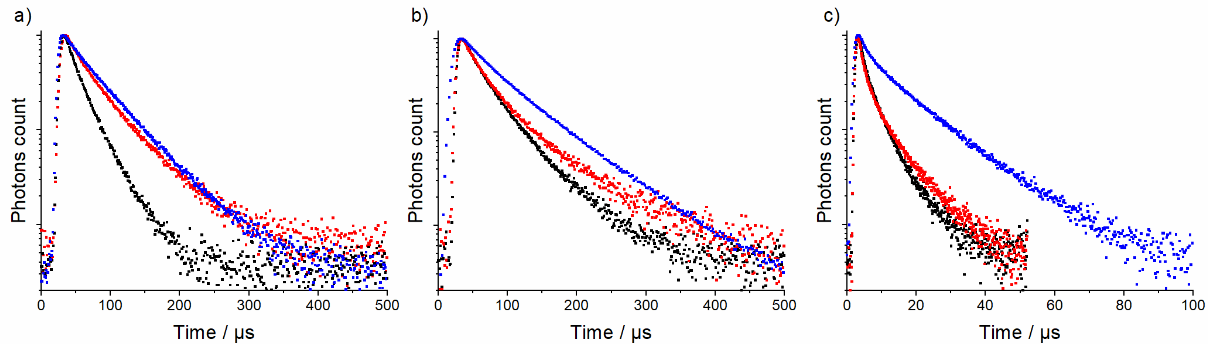

Figure S38: Normalized emission decay profiles (λ_{exc} = 375 nm) of a) $[Mo_6Br_8Cl_6]^{2-}$, b) $[Mo_6Cl_8Cl_6]^{2-}$ and c) $[Mo_6I_8(OCOC_2F_5)_6]^{2-}$ with **$[(C_{10})G]^+$** (in black), **$[(C_{12})G]^+$** (in red), **$[(C_{14})G]^+$** (in blue).

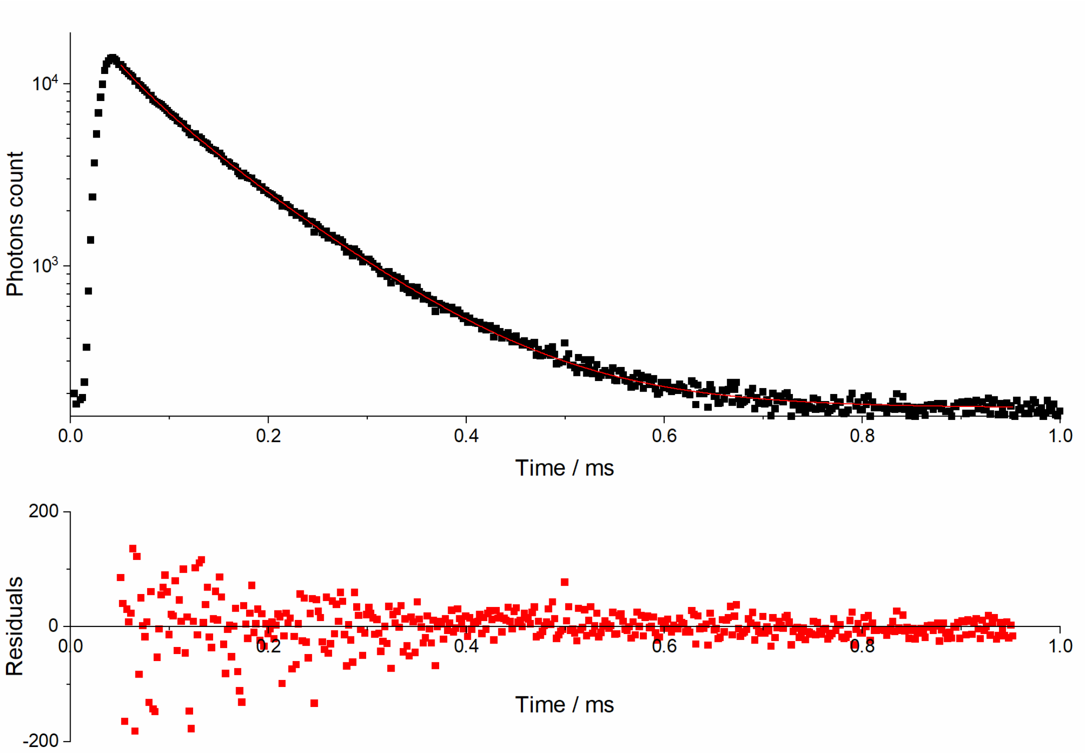

Figure S39: Emission decay profile (black squares), corresponding fitted curve (red line) and fit residuals for $K_2[Mo_6Br_8Cl_6]$.

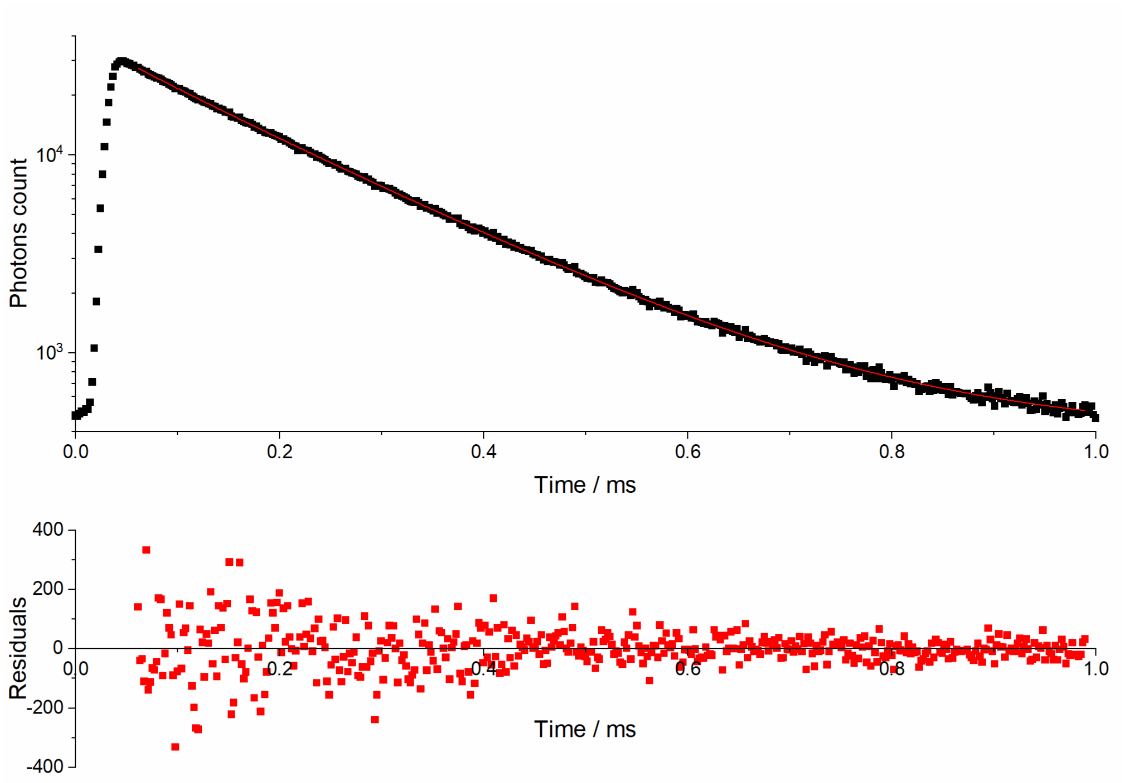

Figure S40: Emission decay profile (black squares), corresponding fitted curve (red line) and fit residuals for $Cs_2[Mo_6Cl_8Cl_6]$.

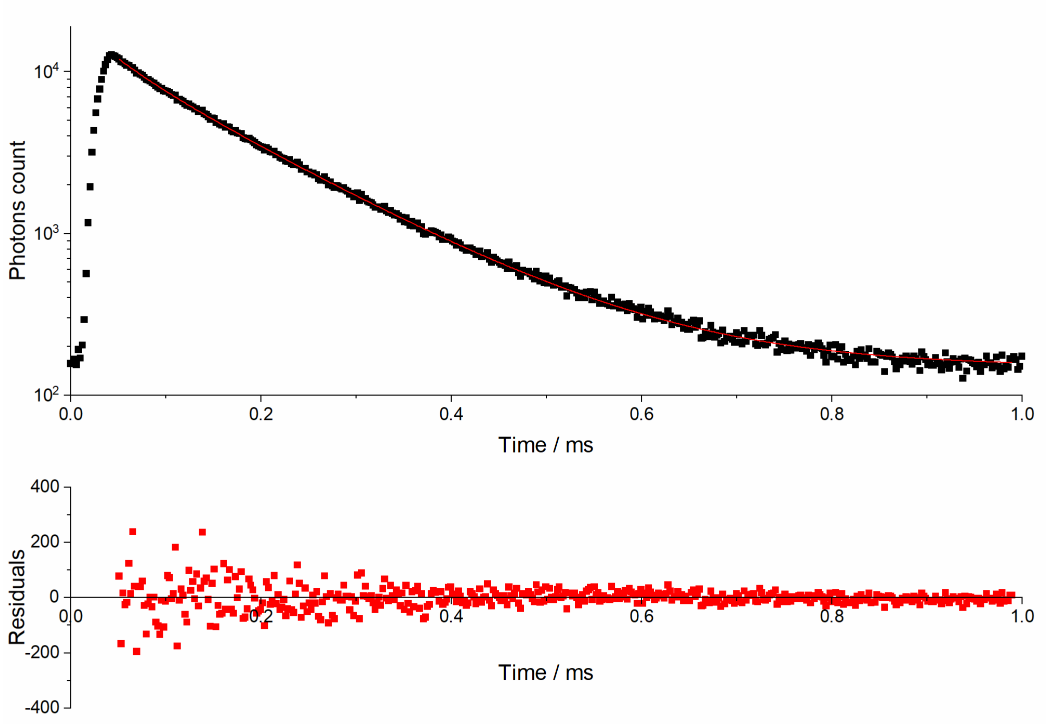

Figure S41: Emission decay profile (black squares), corresponding fitted curve (red line) and fit residuals for $Cs_2[Mo_6I_8(OCOC_2F_5)_6]$.

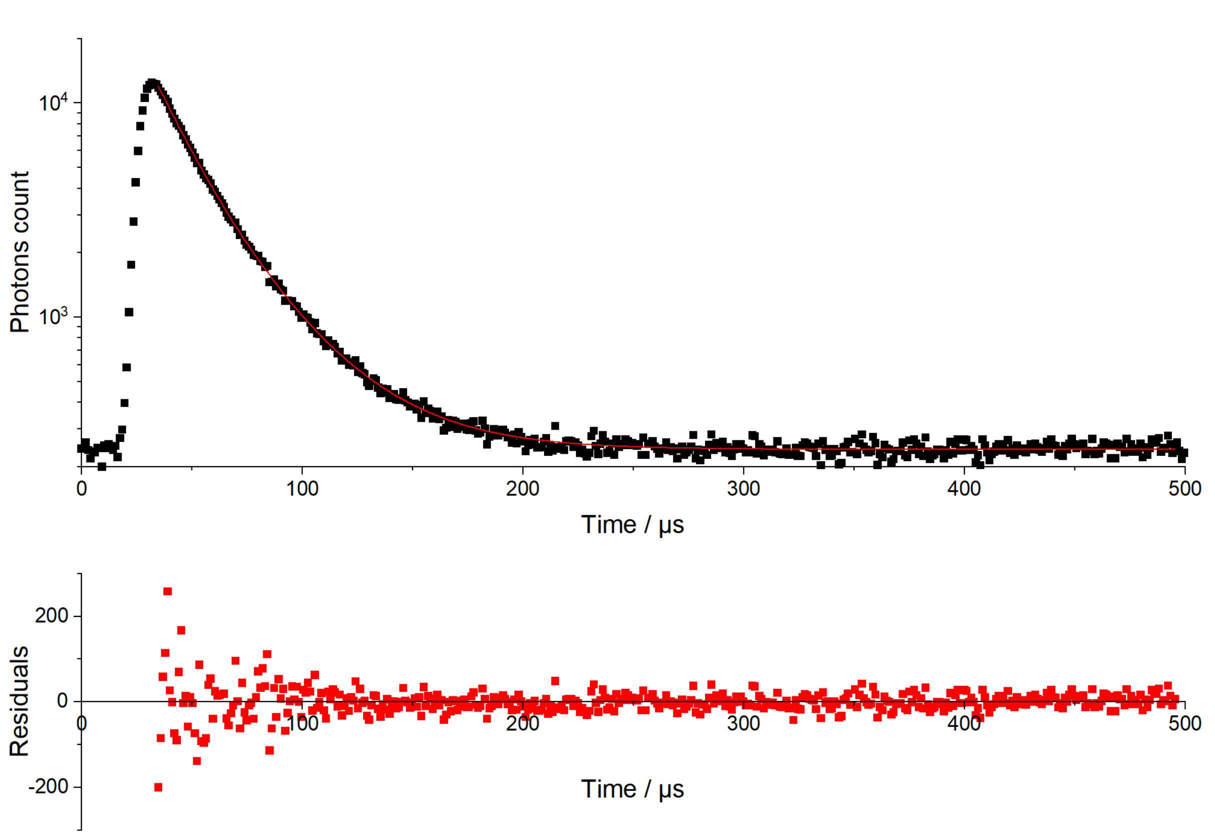

Figure S42: Emission decay profile (black squares), corresponding fitted curve (red line) and fit residuals for **[(C10)G]2[Mo6Br8Cl6]**.

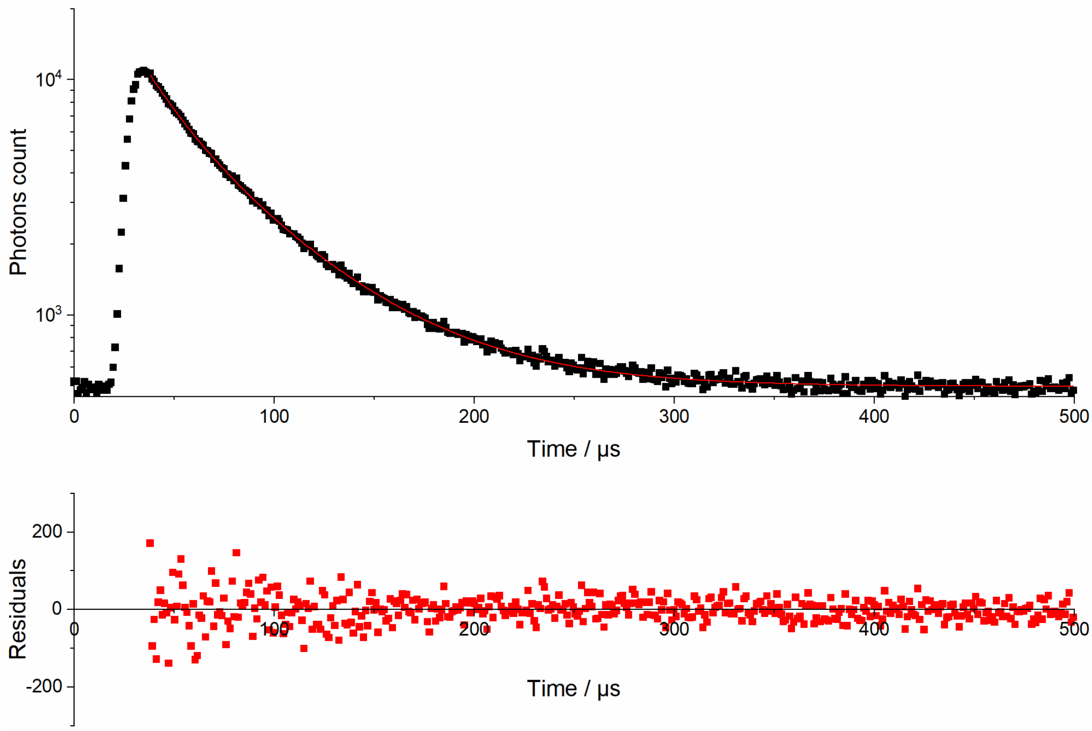

Figure S43: Emission decay profile (black squares), corresponding fitted curve (red line) and fit residuals for **[(C12)G]2[Mo6Br8Cl6]**.

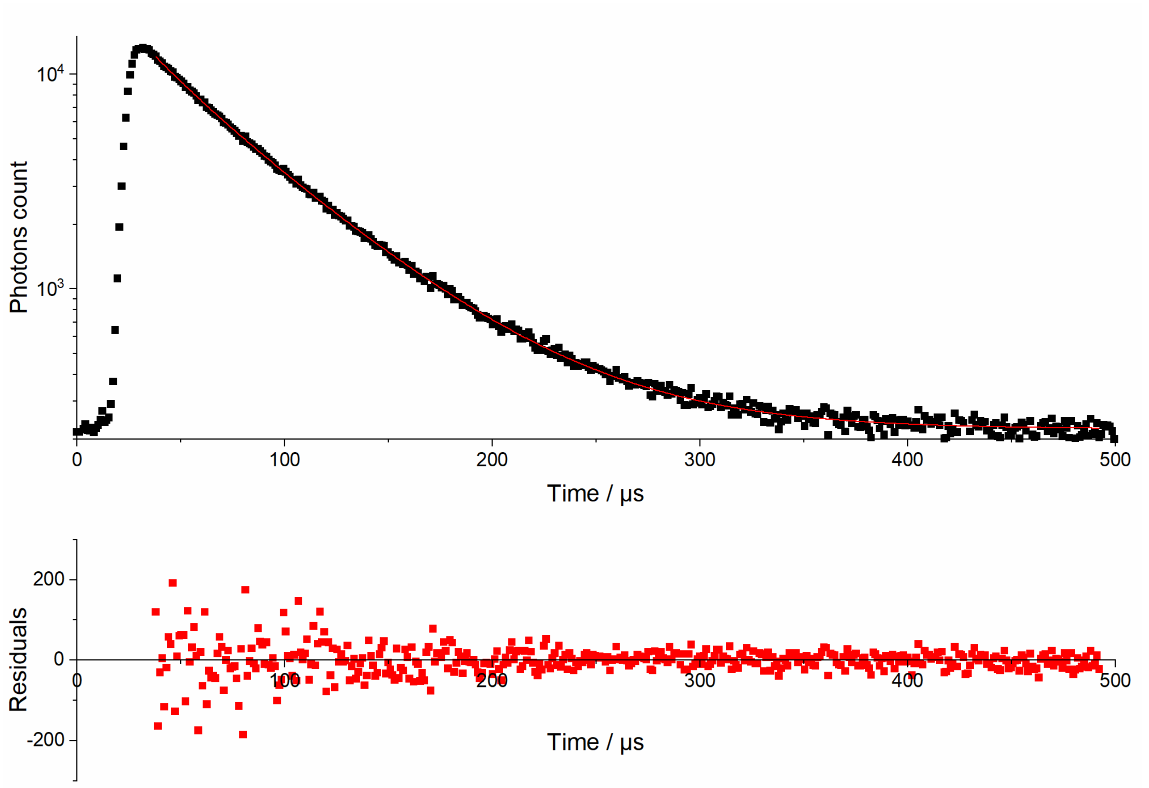

Figure S44: Emission decay profile (black squares), corresponding fitted curve (red line) and fit residuals for **[(C$_{14}$)G]$_2$[Mo$_6$Br$_8$Cl$_6$]**.

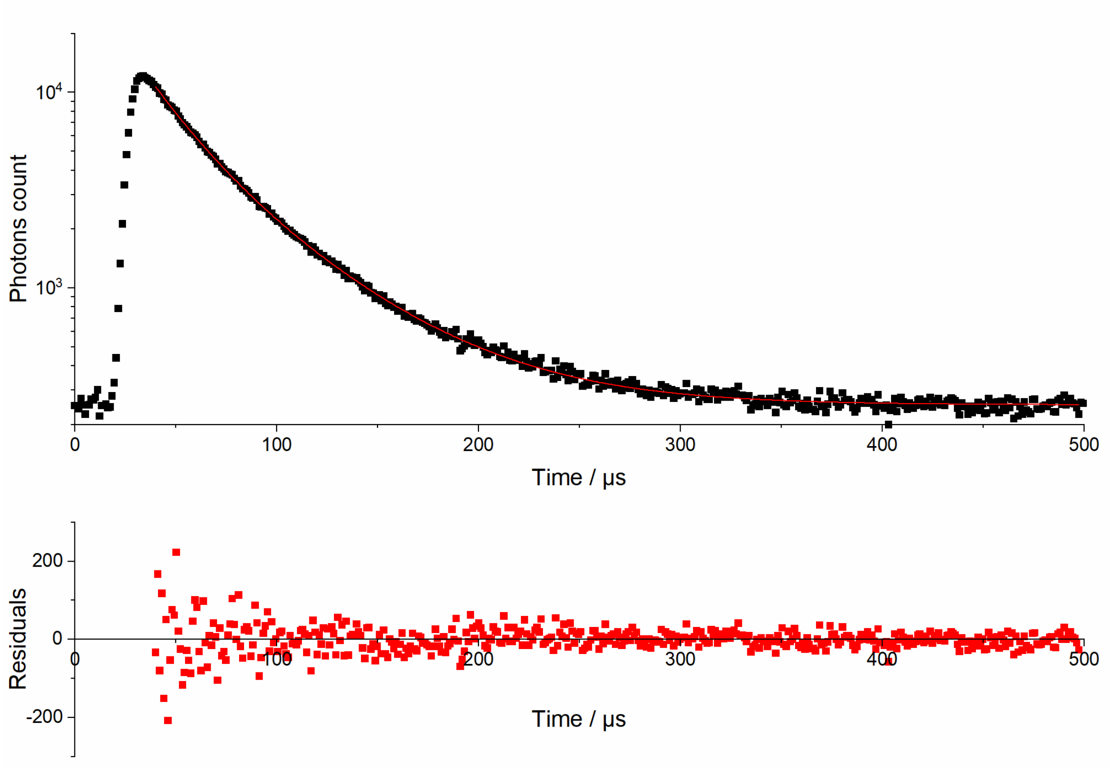

Figure S45: Emission decay profile (black squares), corresponding fitted curve (red line) and fit residuals for **[(C$_{10}$)G]$_2$[Mo$_6$Cl$_8$Cl$_6$]**.

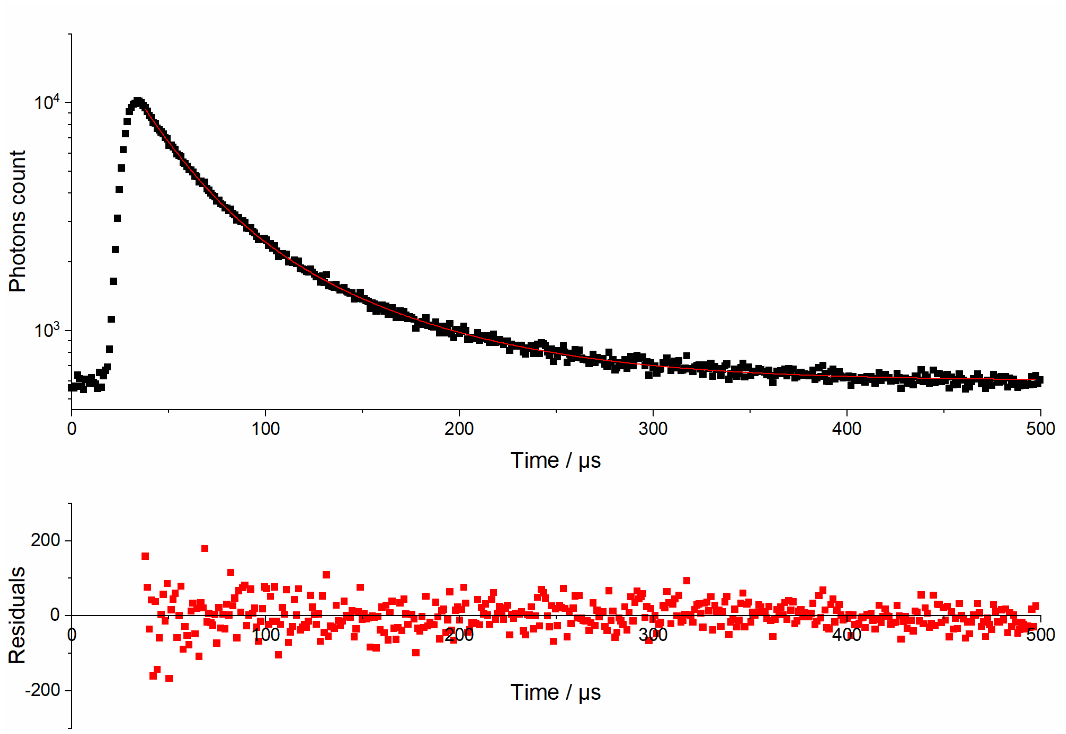

Figure S46: Emission decay profile (black squares), corresponding fitted curve (red line) and fit residuals for **$[(C_{12})G]_2[Mo_6Cl_8Cl_6]$**.

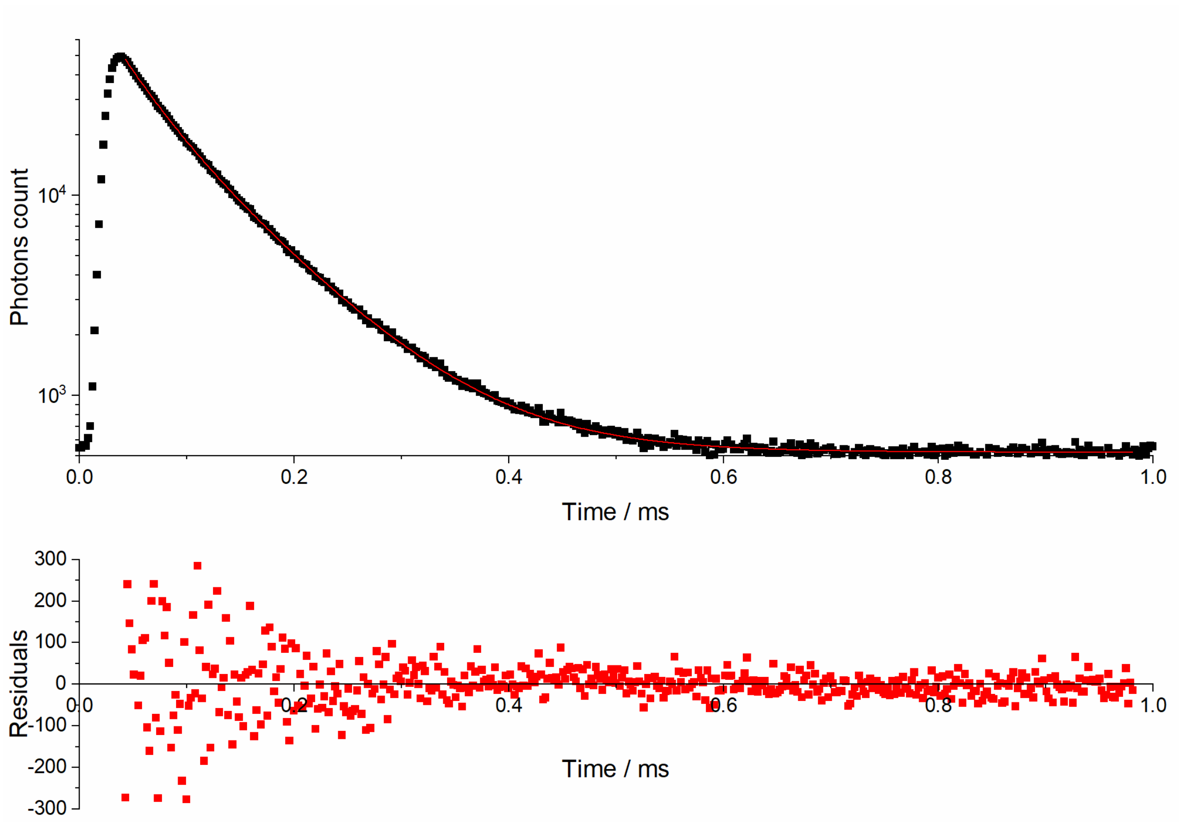

Figure S47: Emission decay profile (black squares), corresponding fitted curve (red line) and fit residuals for **$[(C_{14})G]_2[Mo_6Cl_8Cl_6]$**.

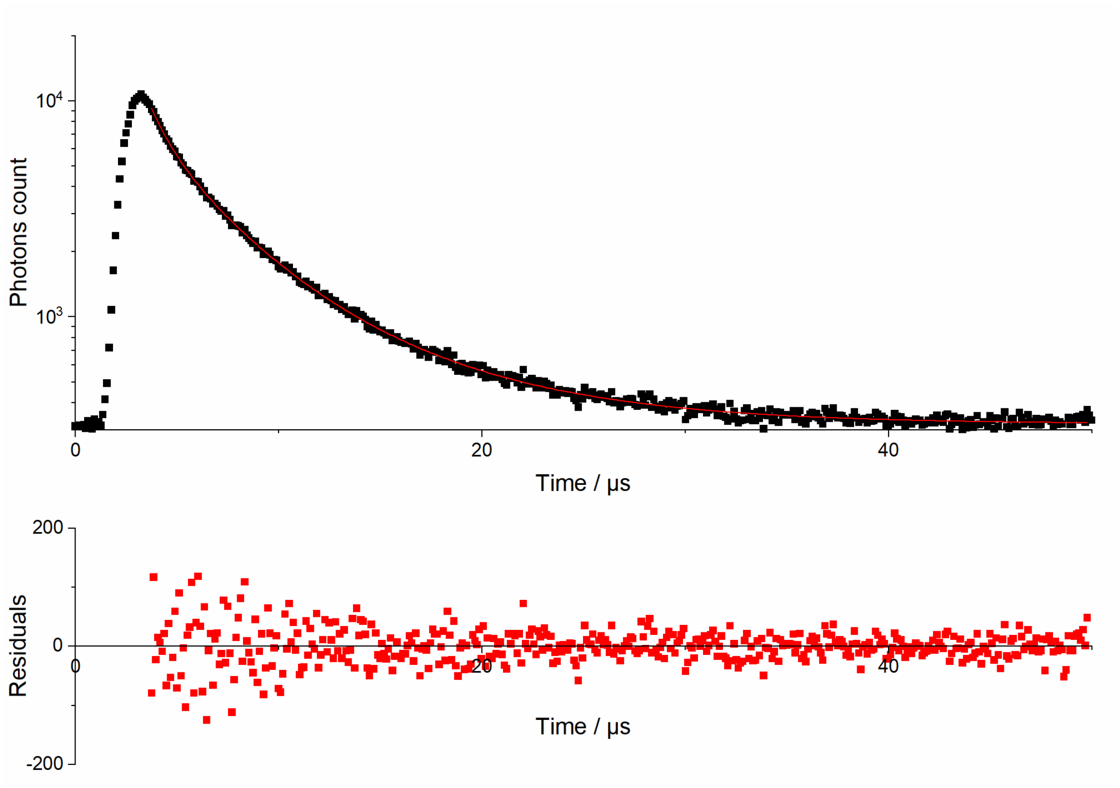

Figure S48: Emission decay profile (black squares), corresponding fitted curve (red line) and fit residuals for **$[(C_{10})G]_2[Mo_6I_8(OCOC_2F_5)_6]$**.

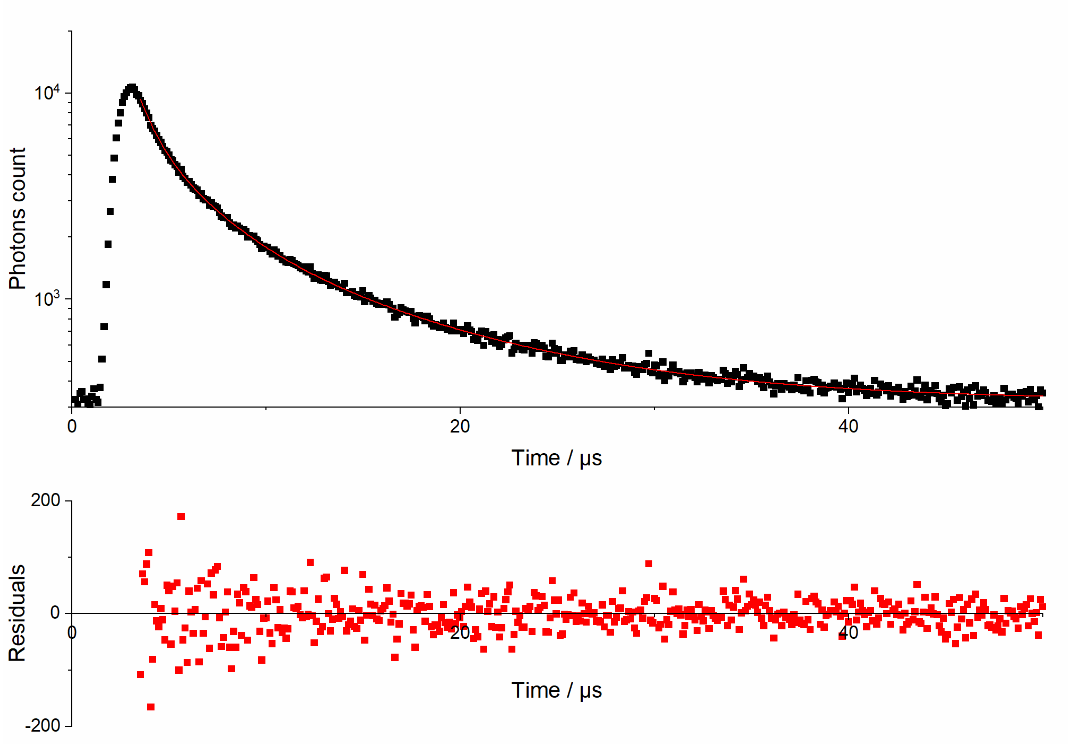

Figure S49: Emission decay profile (black squares), corresponding fitted curve (red line) and fit residuals for **$[(C_{12})G]_2[Mo_6I_8(OCOC_2F_5)_6]$**.

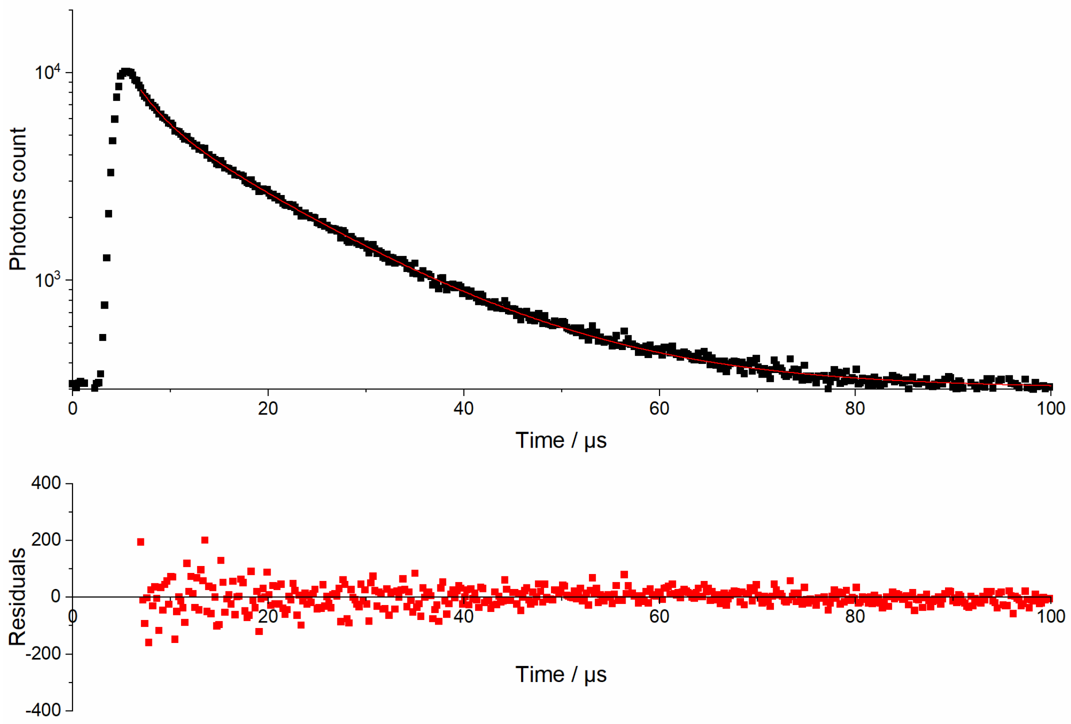

Figure S50: Emission decay profile (black squares), corresponding fitted curve (red line) and fit residuals for **$[(C_{14})G]_2[Mo_6I_8(OCOC_2F_5)_6]$**.

Quenching of the excited state by O_2

To investigate the O_2-dependent quenching of the emission, emission spectra were measured for one cluster hybrid **$[C_{12}G]_2[Mo_6I_8(OCOC_2F_5)]$** under air and under vacuum and the lifetimes were assessed. The emission decay observed under vacuum could be fitted with two components (56 µs (0.48) and 147 µs (0.52)) which gives and average value of 124 µs to be compared with the 6 µs observed in air.

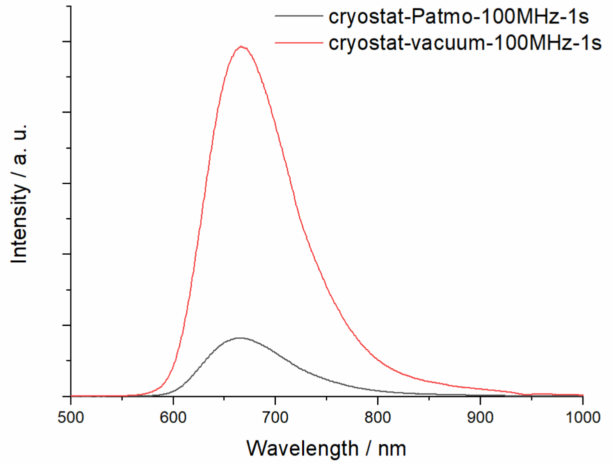

Figure S51: Emission spectra of **$[C_{12}G]_2[Mo_6I_8(OCOC_2F_5)]$** under air (in black) and under vacuum (in red).

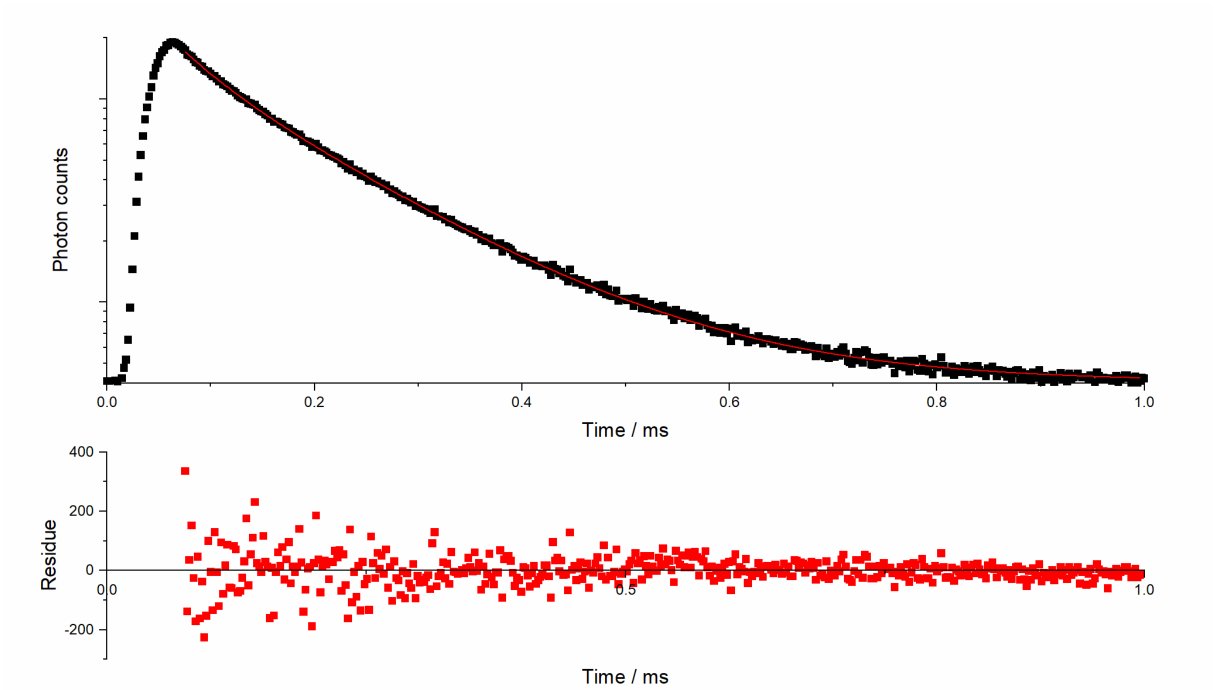

Figure S52: Integrated emission decay profile of **$[C_{12}G]_2[Mo_6I_8(OCOC_2F_5)]$** under vacuum and corresponding fit (in red) with residual.

8) Proposed Packing Models

Columnar Phase

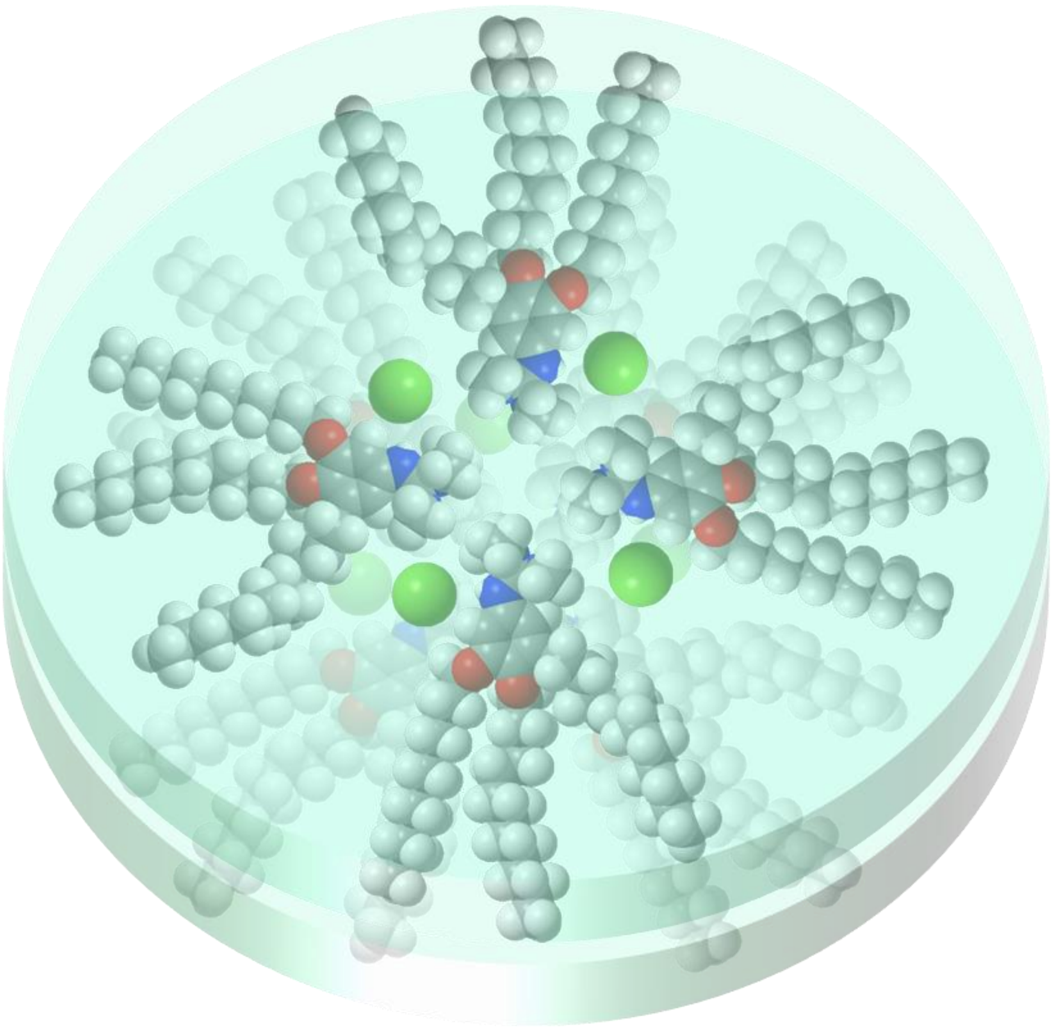

Figure S53: Proposed columnar packing model of **(C_{12})GCl**. Four molecules form a disc which is rotated by 45° to the neighboring discs.

Smectic A Phase

The model set-up in Biovia Materials Studio 2017 R2 started with the ionic mesogen generation (Figure S54, Single Ionic Mesogen). For electrostatic reasons it is not reliable that a negatively charged cluster layer is formed. Therefore, the guanidinium ions have been positioned left and right with respect to a single anionic cluster. - similar to the situation in single crystals (main manuscript Figure 1). The corresponding aliphatic chains, however, point to opposite directions (Figure S54). We decided to optimize a small layer area with 4×4 mesogens. Owing to the diffuse signal corresponding to a cluster distance of 9.7 Å an area size of 38.9×38.9 $Å^2$ has been

chosen. Figure S54 shows such an area. Each cluster is enclosed by four guanidinium ions and the aliphatic tails are pointing alternately up and down. With the cations as spacers the initial distance between the clusters would be much bigger than the experimental value. Therefore, to avoid steric repulsion, half of the mesogens were shifted by 5 Å along the *c* axis. Subsequently, a second layer has been formed and both layers have been placed in a unit cell of $a \times b \times c$ = 38.9×38.9×76.5 Å^3. This has been geometry optimized in the module Forcite using the universal force field, which was working in the presence of the cluster atoms. However, with this force field the aliphatic chains were not optimized satisfactorily. Therefore, we removed the clusters after a partial optimization and optimized the hydrocarbon assembly with the force field COMPASS II to convergence, showing strongly negative van der Waals interactions of the aliphatic chains. Now the clusters were added again and the whole structure geometry optimized with the force field universal. The final structure shown in Figure 2 (main manuscript) delivers a model rationalizing all features found in the SAXS pattern of the shear aligned sample.

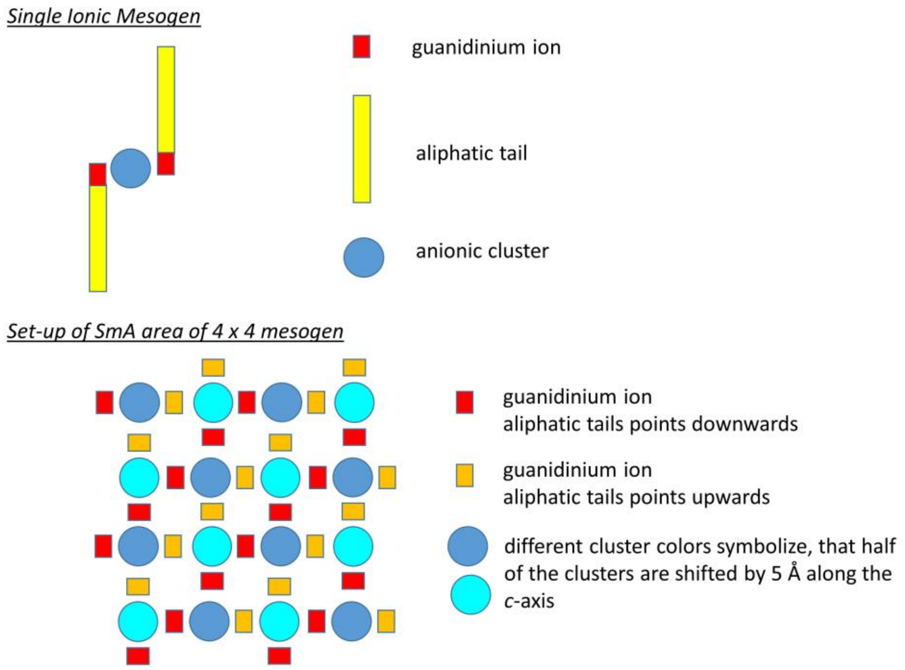

Figure S54: Schematic drawing of the model set-up.

9) NMR Data

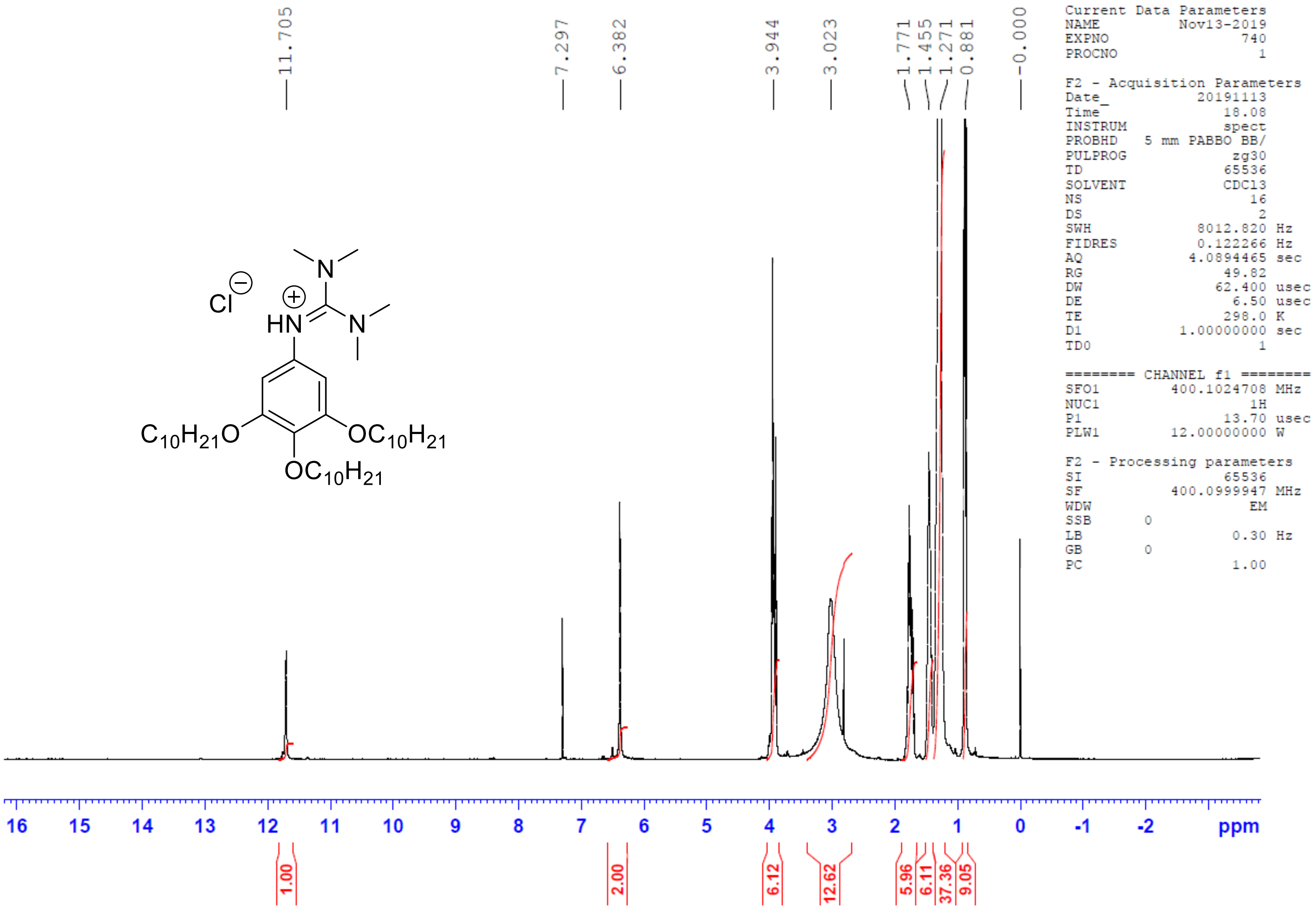

11.705
7.297
6.382
3.944
3.023
1.771
1.455
1.271
0.881
-0.000
Cl
HN
N
N
$C_{10}H_{21}O$
$OC_{10}H_{21}$
$OC_{10}H_{21}$
Current Data Parameters
NAME Nov13-2019
EXPNO 740
PROCNO 1
F2 - Acquisition Parameters
Date_ 20191113
Time 18.08
INSTRUM spect
PROBHD 5 mm PABBO BB/
PULPROG zg30
TD 65536
SOLVENT CDCl3
NS 16
DS 2
SWH 8012.820 Hz
FIDRES 0.122266 Hz
AQ 4.0894465 sec
RG 49.82
DW 62.400 usec
DE 6.50 usec
TE 298.0 K
D1 1.00000000 sec
TD0 1
======== CHANNEL f1 ========
SFO1 400.1024708 MHz
NUC1 1H
P1 13.70 usec
PLW1 12.00000000 W
F2 - Processing parameters
SI 65536
SF 400.0999947 MHz
WDW EM
SSB 0
LB 0.30 Hz
GB 0
PC 1.00
16 15 14 13 12 11 10 9 8 7 6 5 4 3 2 1 0 -1 -2 ppm
1.00
2.00
6.12
12.62
5.96
6.11
37.36
9.05

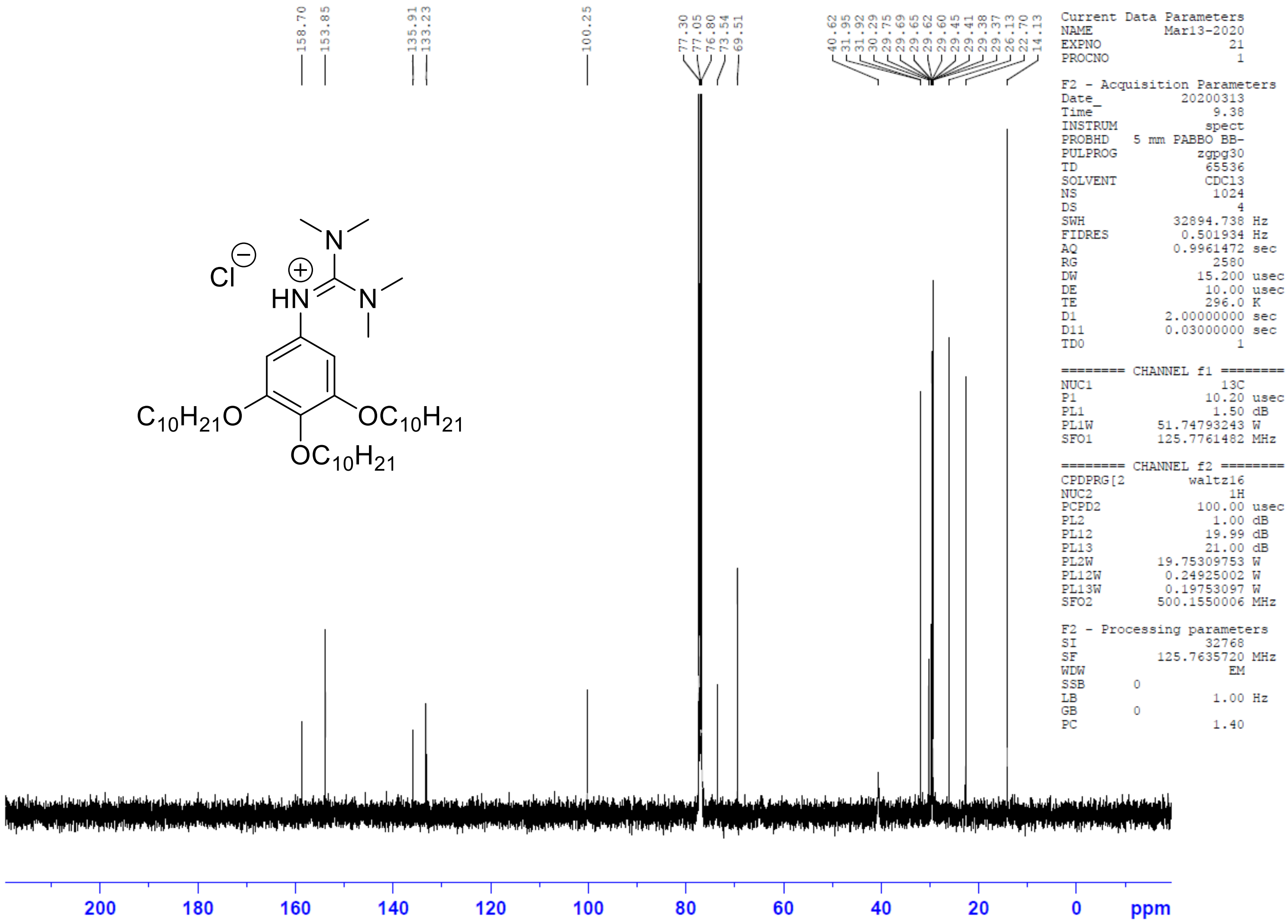


```
Current Data Parameters
NAME             Mar13-2020
EXPNO                    21
PROCNO                    1

F2 - Acquisition Parameters
Date_              20200313
Time                   9.38
INSTRUM               spect
PROBHD     5 mm PABBO BB-
PULPROG              zgpg30
TD                    65536
SOLVENT               CDCl3
NS                     1024
DS                        4
SWH               32894.738 Hz
FIDRES             0.501934 Hz
AQ                0.9961472 sec
RG                     2580
DW                   15.200 usec
DE                    10.00 usec
TE                    296.0 K
D1               2.00000000 sec
D11              0.03000000 sec
TD0                       1

======== CHANNEL f1 ========
NUC1                    13C
P1                    10.20 usec
PL1                    1.50 dB
PL1W            51.74793243 W
SFO1            125.7761482 MHz

======== CHANNEL f2 ========
CPDPRG[2            waltz16
NUC2                     1H
PCPD2                100.00 usec
PL2                    1.00 dB
PL12                  19.99 dB
PL13                  21.00 dB
PL2W            19.75309753 W
PL12W            0.24925002 W
PL13W            0.19753097 W
SFO2            500.1550006 MHz

F2 - Processing parameters
SI                    32768
SF              125.7635720 MHz
WDW                      EM
SSB      0
LB                     1.00 Hz
GB       0
PC                     1.40
```

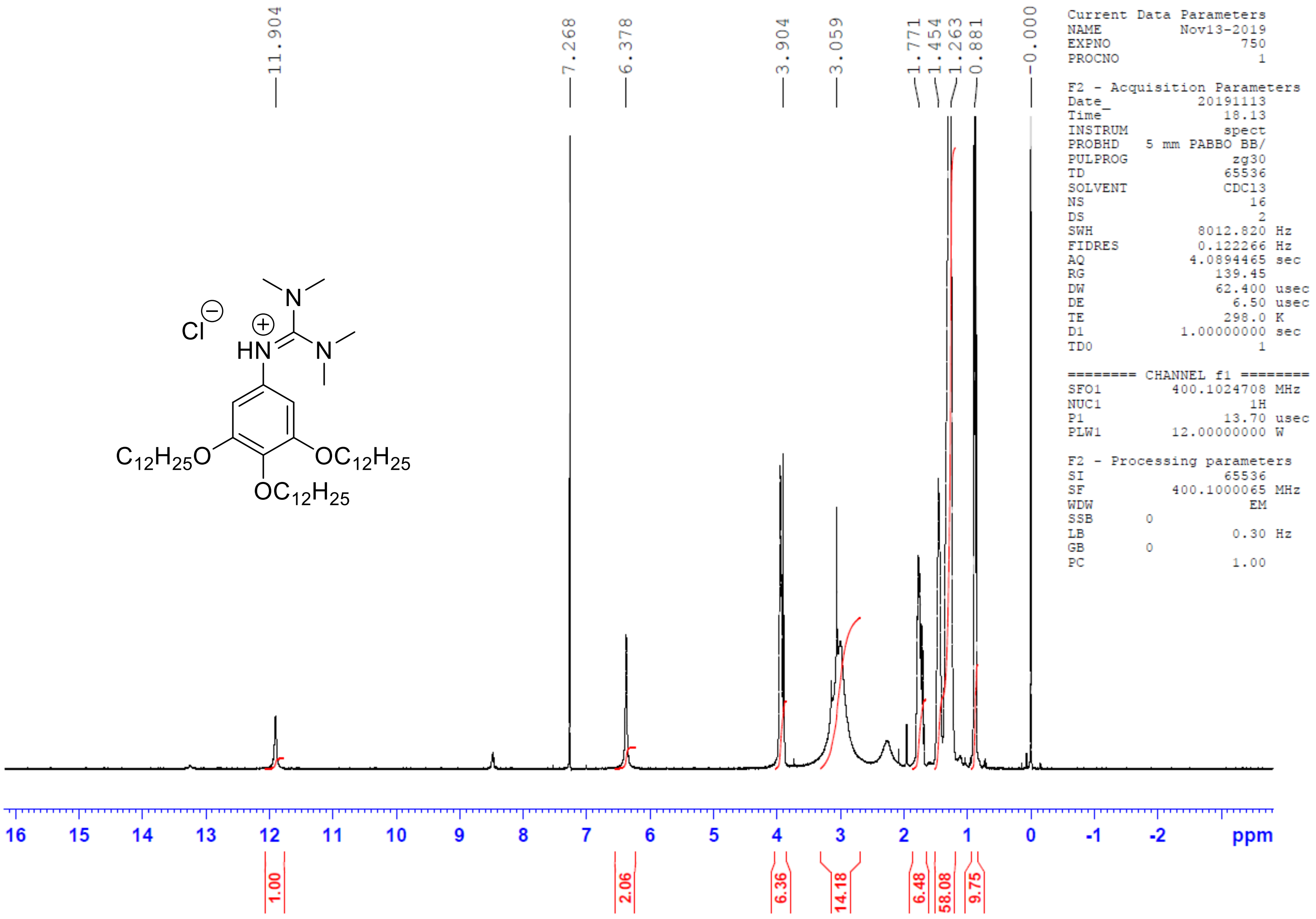
11.904
7.268
6.378
3.904
3.059
1.771
1.454
1.263
0.881
-0.000
Current Data Parameters
NAME Nov13-2019
EXPNO 750
PROCNO 1
F2 - Acquisition Parameters
Date_ 20191113
Time 18.13
INSTRUM spect
PROBHD 5 mm PABBO BB/
PULPROG zg30
TD 65536
SOLVENT CDCl3
NS 16
DS 2
SWH 8012.820 Hz
FIDRES 0.122266 Hz
AQ 4.0894465 sec
RG 139.45
DW 62.400 usec
DE 6.50 usec
TE 298.0 K
D1 1.00000000 sec
TD0 1
======== CHANNEL f1 ========
SFO1 400.1024708 MHz
NUC1 1H
P1 13.70 usec
PLW1 12.00000000 W
F2 - Processing parameters
SI 65536
SF 400.1000065 MHz
WDW EM
SSB 0
LB 0.30 Hz
GB 0
PC 1.00
ppm
1.00
2.06
6.36
14.18
6.48
58.08
9.75

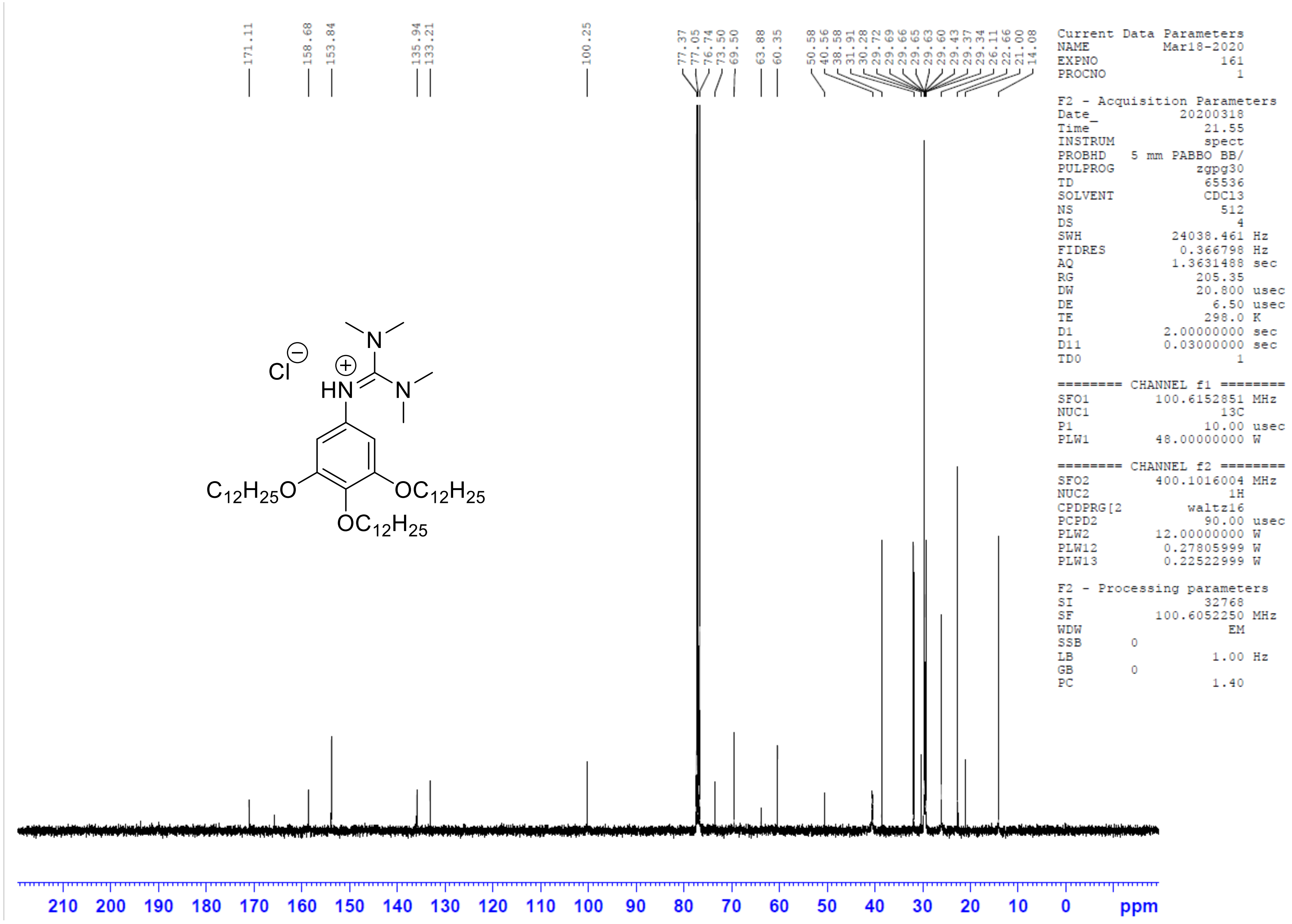

171.11
158.68
153.84
135.94
133.21
100.25
77.37
77.05
76.74
73.50
69.50
63.88
60.35
50.58
40.56
38.58
31.91
30.28
29.72
29.69
29.66
29.65
29.63
29.60
29.43
29.37
29.34
26.11
22.66
21.00
14.08
Current Data Parameters
NAME Mar18-2020
EXPNO 161
PROCNO 1
F2 - Acquisition Parameters
Date_ 20200318
Time 21.55
INSTRUM spect
PROBHD 5 mm PABBO BB/
PULPROG zgpg30
TD 65536
SOLVENT CDCl3
NS 512
DS 4
SWH 24038.461 Hz
FIDRES 0.366798 Hz
AQ 1.3631488 sec
RG 205.35
DW 20.800 usec
DE 6.50 usec
TE 298.0 K
D1 2.00000000 sec
D11 0.03000000 sec
TD0 1
======== CHANNEL f1 ========
SFO1 100.6152851 MHz
NUC1 13C
P1 10.00 usec
PLW1 48.00000000 W
======== CHANNEL f2 ========
SFO2 400.1016004 MHz
NUC2 1H
CPDPRG[2 waltz16
PCPD2 90.00 usec
PLW2 12.00000000 W
PLW12 0.27805999 W
PLW13 0.22522999 W
F2 - Processing parameters
SI 32768
SF 100.6052250 MHz
WDW EM
SSB 0
LB 1.00 Hz
GB 0
PC 1.40
Cl
HN
N
N
C12H25O
OC12H25
OC12H25
210 200 190 180 170 160 150 140 130 120 110 100 90 80 70 60 50 40 30 20 10 0 ppm

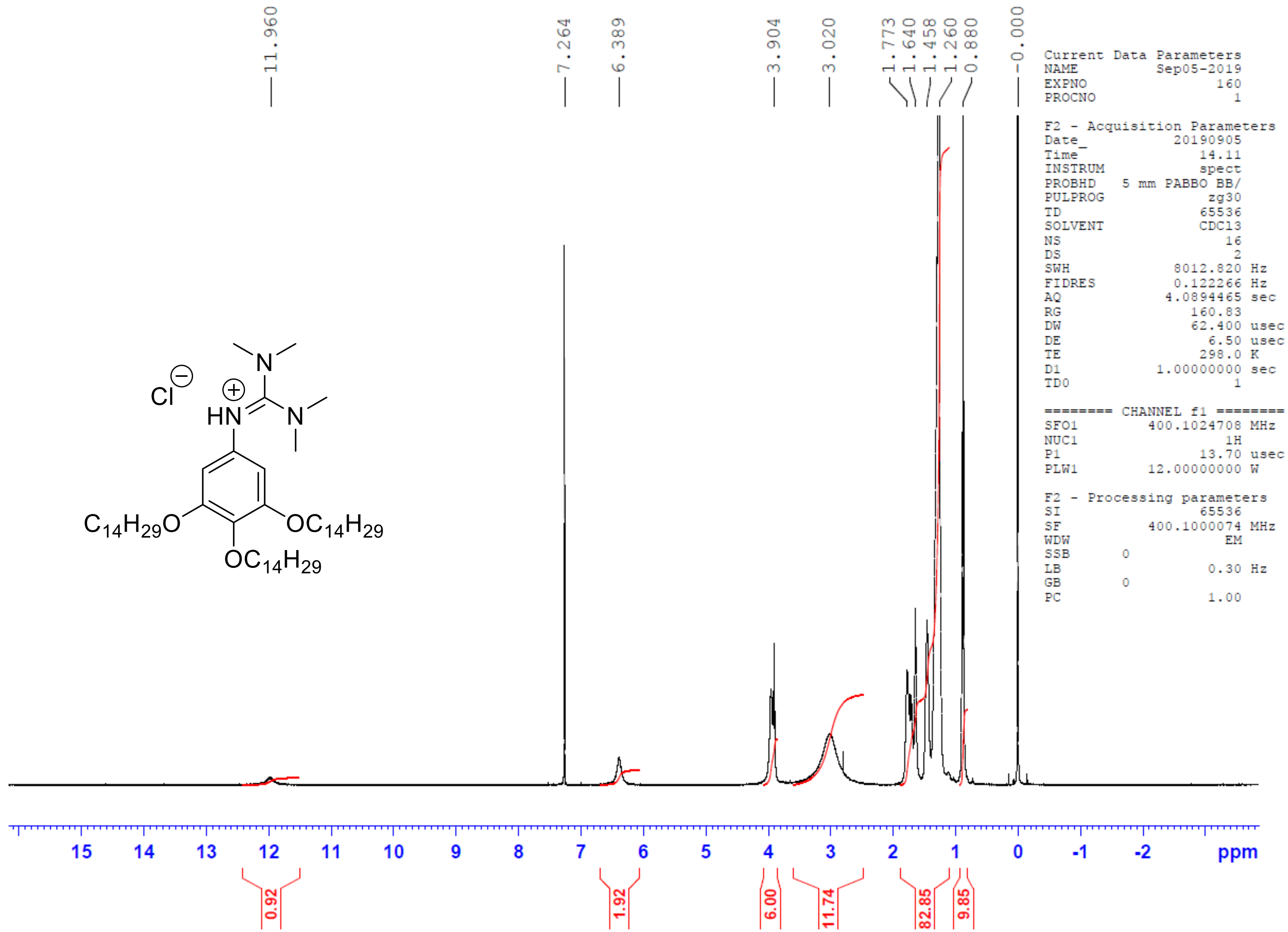
11.960
7.264
6.389
3.904
3.020
1.773
1.640
1.458
1.260
0.880
-0.000
Current Data Parameters
NAME Sep05-2019
EXPNO 160
PROCNO 1
F2 - Acquisition Parameters
Date_ 20190905
Time 14.11
INSTRUM spect
PROBHD 5 mm PABBO BB/
PULPROG zg30
TD 65536
SOLVENT CDCl3
NS 16
DS 2
SWH 8012.820 Hz
FIDRES 0.122266 Hz
AQ 4.0894465 sec
RG 160.83
DW 62.400 usec
DE 6.50 usec
TE 298.0 K
D1 1.00000000 sec
TD0 1
======== CHANNEL f1 ========
SFO1 400.1024708 MHz
NUC1 1H
P1 13.70 usec
PLW1 12.00000000 W
F2 - Processing parameters
SI 65536
SF 400.1000074 MHz
WDW EM
SSB 0
LB 0.30 Hz
GB 0
PC 1.00
Cl
HN
N
N
C14H29O
OC14H29
OC14H29
15 14 13 12 11 10 9 8 7 6 5 4 3 2 1 0 -1 -2 ppm
0.92
1.92
6.00
11.74
82.85
9.85

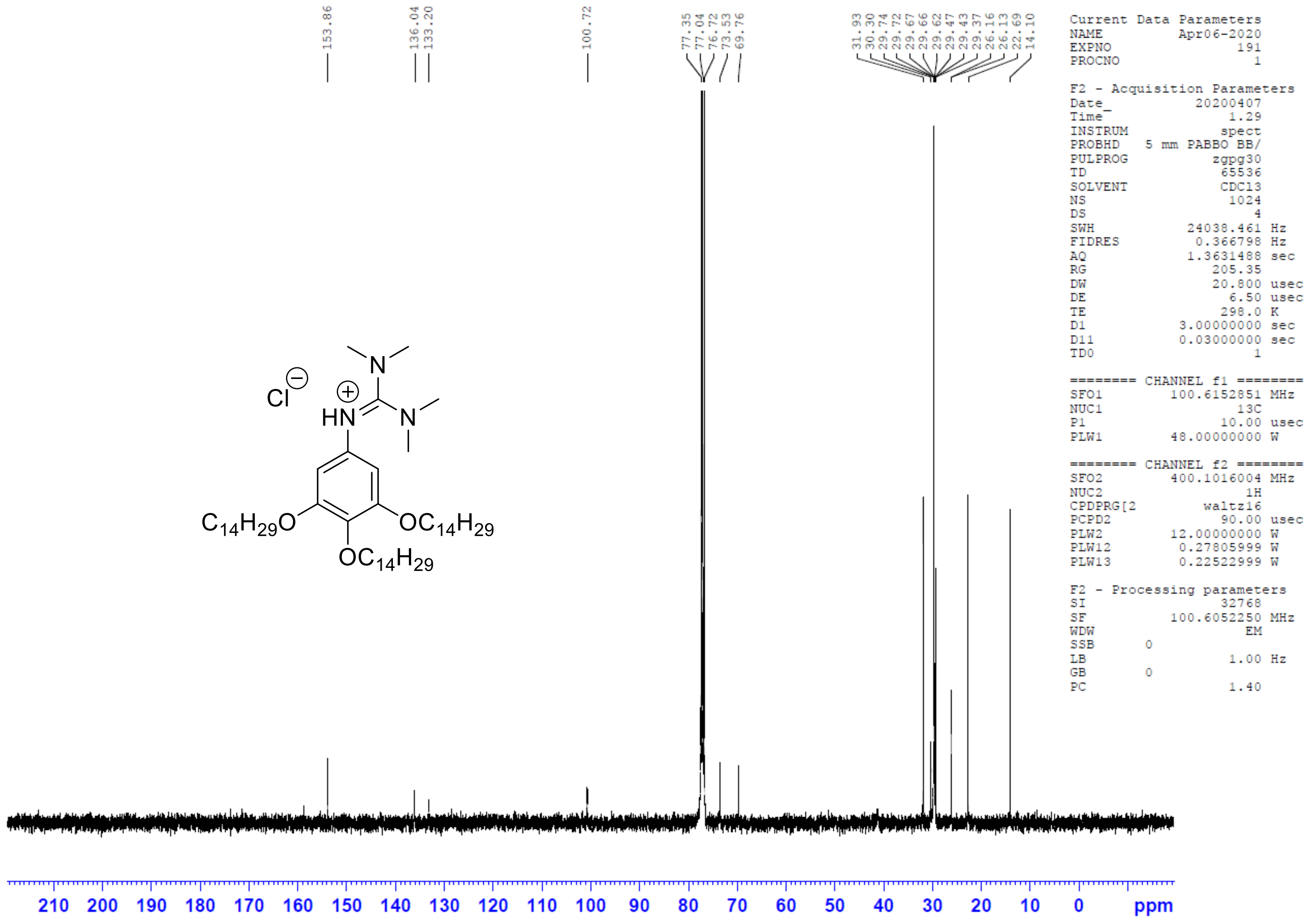
153.86
136.04
133.20
100.72
77.35
77.04
76.72
73.53
69.76
31.93
30.30
29.74
29.72
29.67
29.66
29.62
29.47
29.43
29.37
26.16
26.13
22.69
14.10
Cl
HN
N
N
C14H29O
OC14H29
OC14H29
Current Data Parameters
NAME Apr06-2020
EXPNO 191
PROCNO 1
F2 - Acquisition Parameters
Date_ 20200407
Time 1.29
INSTRUM spect
PROBHD 5 mm PABBO BB/
PULPROG zgpg30
TD 65536
SOLVENT CDCl3
NS 1024
DS 4
SWH 24038.461 Hz
FIDRES 0.366798 Hz
AQ 1.3631488 sec
RG 205.35
DW 20.800 usec
DE 6.50 usec
TE 298.0 K
D1 3.00000000 sec
D11 0.03000000 sec
TD0 1
======== CHANNEL f1 ========
SFO1 100.6152851 MHz
NUC1 13C
P1 10.00 usec
PLW1 48.00000000 W
======== CHANNEL f2 ========
SFO2 400.1016004 MHz
NUC2 1H
CPDPRG[2 waltz16
PCPD2 90.00 usec
PLW2 12.00000000 W
PLW12 0.27805999 W
PLW13 0.22522999 W
F2 - Processing parameters
SI 32768
SF 100.6052250 MHz
WDW EM
SSB 0
LB 1.00 Hz
GB 0
PC 1.40
210 200 190 180 170 160 150 140 130 120 110 100 90 80 70 60 50 40 30 20 10 0 ppm

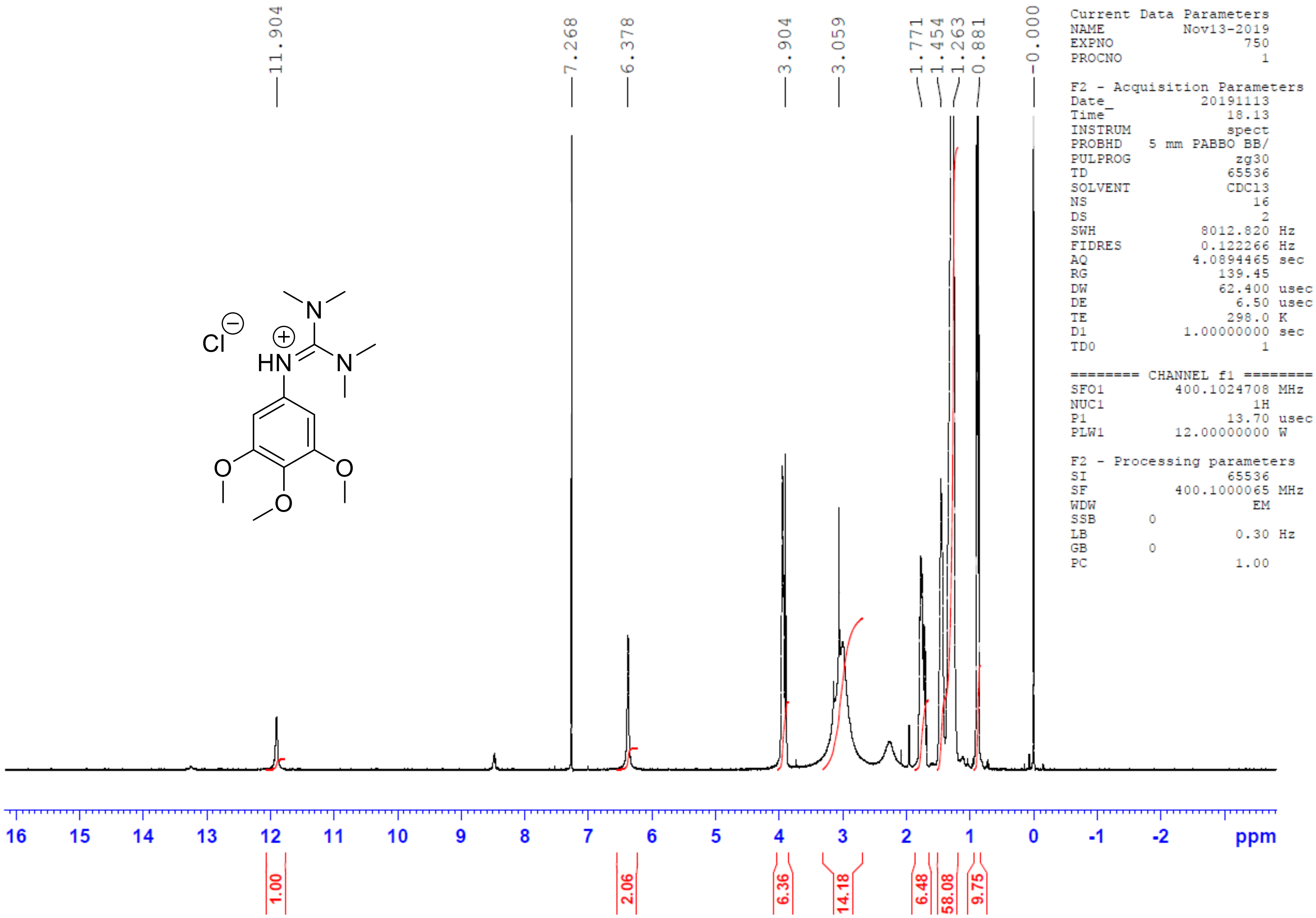


```
Current Data Parameters
NAME          Nov13-2019
EXPNO                750
PROCNO                 1

F2 - Acquisition Parameters
Date_           20191113
Time               18.13
INSTRUM            spect
PROBHD   5 mm PABBO BB/
PULPROG             zg30
TD                 65536
SOLVENT            CDCl3
NS                    16
DS                     2
SWH            8012.820 Hz
FIDRES         0.122266 Hz
AQ            4.0894465 sec
RG                139.45
DW                62.400 usec
DE                  6.50 usec
TE                 298.0 K
D1            1.00000000 sec
TD0                    1

======== CHANNEL f1 ========
SFO1        400.1024708 MHz
NUC1                  1H
P1                 13.70 usec
PLW1         12.00000000 W

F2 - Processing parameters
SI                 65536
SF           400.1000065 MHz
WDW                   EM
SSB      0
LB                  0.30 Hz
GB       0
PC                  1.00
```

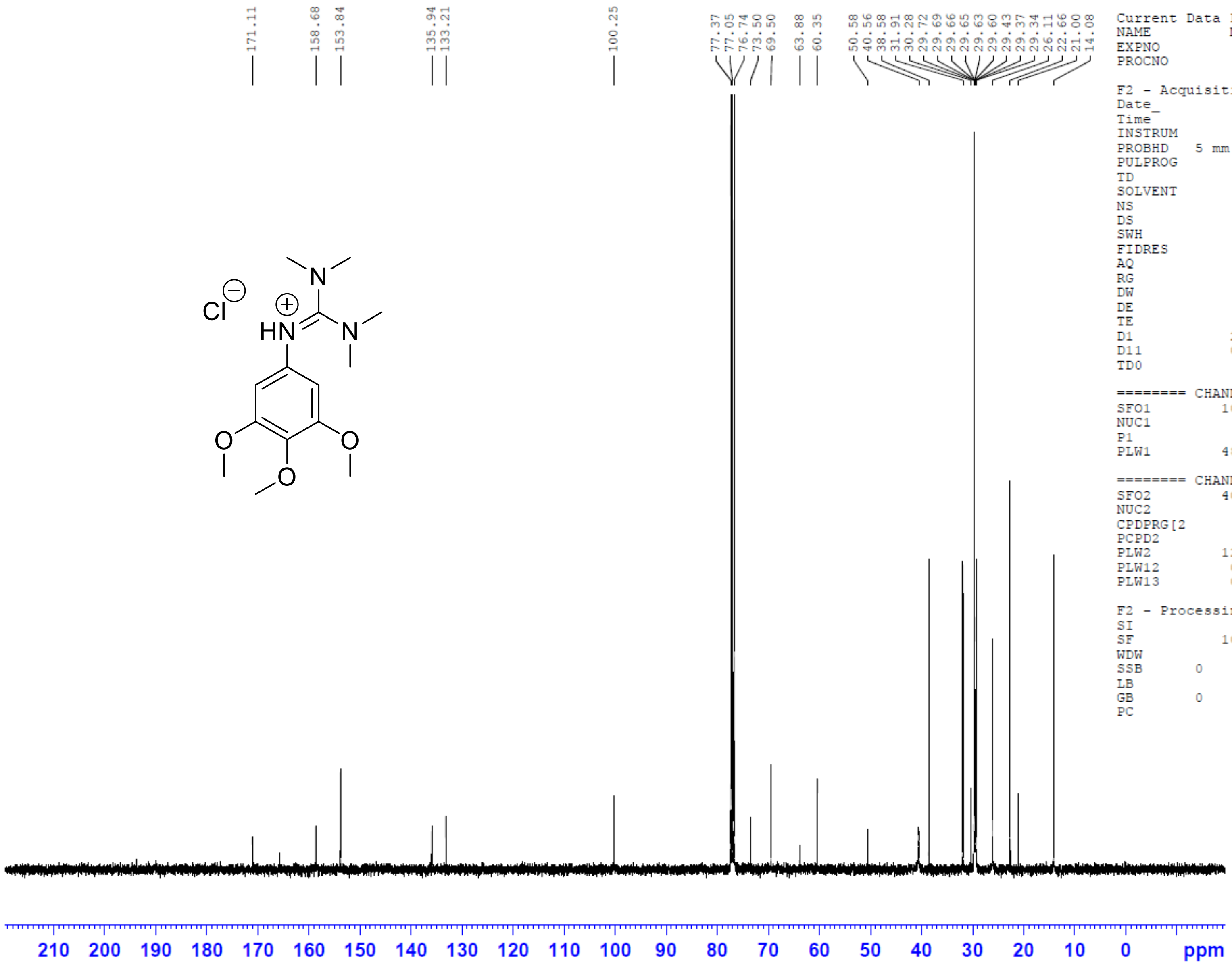

Current Data Parameters

NAME	Mar18-2020
EXPNO	161
PROCNO	1

F2 - Acquisition Parameters

Date_	20200318
Time	21.55
INSTRUM	spect
PROBHD	5 mm PABBO BB/
PULPROG	zgpg30
TD	65536
SOLVENT	CDCl3
NS	512
DS	4
SWH	24038.461 Hz
FIDRES	0.366798 Hz
AQ	1.3631488 sec
RG	205.35
DW	20.800 usec
DE	6.50 usec
TE	298.0 K
D1	2.00000000 sec
D11	0.03000000 sec
TD0	1

======== CHANNEL f1 ========

SFO1	100.6152851 MHz
NUC1	13C
P1	10.00 usec
PLW1	48.00000000 W

======== CHANNEL f2 ========

SFO2	400.1016004 MHz
NUC2	1H
CPDPRG[2	waltz16
PCPD2	90.00 usec
PLW2	12.00000000 W
PLW12	0.27805999 W
PLW13	0.22522999 W

F2 - Processing parameters

SI	32768
SF	100.6052250 MHz
WDW	EM
SSB	0
LB	1.00 Hz
GB	0
PC	1.40

10) References

[1] J. De, I. Bala, S. P. Gupta, U. K. Pandey, S. K. Pal, *J. Am. Chem. Soc.* **2019**, *141*, 47, 18799–18805. (https://doi.org/10.1021/jacs.9b09126)

[2] R. Tapia, G. Torres, J.A. Valderrama, *Synth. Comm.* **1986**, *16* (6), 681–687. (https://doi.org/10.1080/00397918608057740)

[3] V. Percec, M. Peterca, M. J. Sienkowska, M. A. Ilies, E. Aquad, J. Smidrkal, P. A. Heiney, *Angew. Chem. Int. Ed.* **2005**, *44*, 4739 –4745. (https://doi.org/10.1002/anie.200501254)

[4] M. Huang, L. Wang, X. Zhu, Z. Mao, D. Kuang, Y. Wang, *Eur. J. Org. Chem.* **2012**, 4897–4901. (https://doi.org/10.1002/ejoc.201200787)

[5] N. Saito, P. Lemoine, N. Dumait, M. Amela-Cortes, S. Paofai, T. Roisnel, V. Nassif, F. Grasset, Y. Wada, N. Ohashi, S. Cordier, *J. Cluster Science* **2017**, *28*, 773–798. (https://doi.org/10.1007/s10876-016-1133-5)

[6] F. W. Koknat, T. J. Adaway, S. I. Erzerum, S. Syed, *Inorg. Nucl. Chem. Lett.* **1980**, *16*, 307–310. (https://doi.org/10.1016/0020-1650(80)80066-3)

[7] K. Kirakci, S. Cordier, C. Perrin, *Z. Anorg. Allg. Chem.* **2005**, *631*, 411–416. (https://doi.org/10.1002/zaac.200400281)

[8] W. Preetz, D. Bublitz, H. G. von Schnering, J. Saßmannshausen, *Z. Anorg. Allg. Chem.* **1994**, *620*, 234–246. (https://doi.org/10.1002/zaac.19946200207)

[9] M. Amela-Cortes, Y. Molard, S. Paofai, A. Desert, J.-L. Duvail, N. G. Naumov, S. Cordier, *Dalton Trans.* **2016**, *45*, 237–245. (https://doi.org/10.1039/C5DT03734D)
